# Therapeutic Hypothermia in Low-Risk Nonpumped Brain-Dead Kidney Donors: A Randomized Clinical Trial

**DOI:** 10.1001/jamanetworkopen.2023.53785

**Published:** 2024-02-28

**Authors:** Madhukar S. Patel, Juan D. Salcedo-Betancourt, Christina Saunders, Kristine Broglio, Darren Malinoski, Claus U. Niemann

**Affiliations:** 1 Division of Surgical Transplantation, University of Texas Southwestern Medical Center, Dallas; 2 Department of Medicine, University of Texas Southwestern Medical Center, Dallas; 3 Berry Consultants, LLC, Austin, Texas; 4 Oncology Statistical Innovation, AstraZeneca, Gaithersburg, Maryland; 5 Division of Trauma, Critical Care, and Acute Care Surgery, Oregon Health & Science University, Portland; 6 Department of Anesthesia and Perioperative Care, University of California, San Francisco, San Francisco, California; 7 Division of Transplantation, Department of Surgery, University of California San Francisco, San Francisco, California

## Abstract

**Importance:**

Delayed graft function in kidney-transplant recipients is associated with increased financial cost and patient burden. In donors with high Kidney Donor Profile Index whose kidneys are not pumped, therapeutic hypothermia has been shown to confer a protective benefit against delayed graft function.

**Objective:**

To determine whether hypothermia is superior to normothermia in preventing delayed graft function in low-risk nonpumped kidney donors after brain death.

**Design, Setting, and Participants:**

In a multicenter randomized clinical trial, brain-dead kidney donors deemed to be low risk and not requiring machine perfusion per Organ Procurement Organization protocol were prospectively randomized to hypothermia (34.0-35 °C) or normothermia (36.5-37.5 °C) between August 10, 2017, and May 21, 2020, across 4 Organ Procurement Organizations in the US (Arizona, Upper Midwest, Pacific Northwest, and Texas). The final analysis report is dated June 15, 2022, based on the data set received from the United Network for Organ Sharing on June 2, 2021. A total of 509 donors (normothermia: n = 245 and hypothermia: n = 236; 1017 kidneys) met inclusion criteria over the study period.

**Intervention:**

Donor hypothermia (34.0-35.0 °C) or normothermia (36.5-37.5 °C).

**Main Outcomes and Measures:**

The primary outcome was delayed graft function in the kidney recipients, defined as the need for dialysis within the first week following kidney transplant. The primary analysis follows the intent-to-treat principle.

**Results:**

A total of 934 kidneys were transplanted from 481 donors, of which 474 were randomized to the normothermia group and 460 to the hypothermia group. Donor characteristics were similar between the groups, with overall mean (SD) donor age 34.2 (11.1) years, and the mean donor creatinine level at enrollment of 1.03 (0.53) mg/dL. There was a predominance of Standard Criteria Donors (98% in each treatment arm) with similar low mean (SD) Kidney Donor Profile Index (normothermia: 28.99 [20.46] vs hypothermia: 28.32 [21.9]). Cold ischemia time was similar in the normothermia and hypothermia groups (15.99 [7.9] vs 15.45 [7.63] hours). Delayed graft function developed in 87 of the recipients (18%) in the normothermia group vs 79 (17%) in the hypothermia group (adjusted odds ratio, 0.92; 95% CI, 0.64-1.33; *P* = .66).

**Conclusions and Relevance:**

The findings of this study suggest that, in low-risk non-pumped kidneys from brain-dead kidney donors, therapeutic hypothermia compared with normothermia does not appear to prevent delayed graft function in kidney transplant recipients.

**Trial Registration:**

ClinicalTrials.gov Identifier: NCT02525510

## Key Points

**Question:**

In lower-risk nonpumped brain-dead kidney donors, is donor hypothermia superior to normothermia in preventing delayed graft function?

**Findings:**

In this randomized clinical trial that included 509 kidney donors, delayed graft function developed in 87 cases (18%) in the normothermia group vs 79 cases (17%) in the hypothermia group, a nonsignificant difference.

**Meaning:**

The findings of this study suggest that, in low-risk nonpumped kidneys from brain-dead kidney donors, therapeutic hypothermia compared with normothermia does not appear to prevent delayed graft function in kidney transplant recipients.

## Teaser

This randomized clinical trial examines the development of delayed graft function in recipients of kidney transplant with use of hypothermia vs normothermia in nonpumped kidneys from donors after brain death.

## Introduction

Delayed graft function (DGF), defined as the need for dialysis within the first week following kidney transplant,^1^ is a common complication occurring in 25% to 31% of deceased donor transplant recipients in the US.^2,3^ Historically, it has been associated with poorer clinical outcomes, including increased risk of acute rejection and shorter graft survival,^4,5^ as well as increased costs and length of hospital stay.^6^ Major risk factors for DGF include those associated with a higher risk allograft.^7,8^ Specifically, in the assessment of a deceased donor kidney quality, traditional risk factors included history of hypertension, donor age older than 60 years, terminal serum creatinine level greater than 1.5 mg/dL (to convert to micromoles per liter, multiply by 88.4), and/or death from a cerebrovascular accident, based on which a kidney was deemed lower risk (Standard Criteria Donor [SCD]) or higher risk (Extended Criteria Donor [ECD]). More recently, the Kidney Donor Profile Index (KDPI) has been used as it combines 10 donor demographic and clinical parameters to yield a numeric quantification of deceased donor kidney quality relative to other grafts. In addition to these donor parameters, the presence of human leukocyte antigen antibodies and prolonged cold ischemia time increase the risk of DGF.^7,8^

The pathophysiologic factors underlying DGF are thought to be caused by acute tubular necrosis from ischemia reperfusion injury.^9,10^ During organ procurement, blood flow deprivation and ischemia trigger a series of biochemical cascades leading to ischemia reperfusion injury.^11,12^ Cold exposure reduces cellular metabolism and tissue oxygen demands, and tailored hypothermia has been shown to mitigate ischemia-induced tissue injury when used as a method to prevent ischemia reperfusion injury.^12^ Ex vivo organ cooling (either static cold storage or hypothermic machine perfusion) has been traditionally considered the cornerstone organ preservation strategy to mitigate hypoxic injury.^13,14^ More recently, deceased donor therapeutic hypothermia has been proposed as a preventive strategy against the development of DGF.^15^

In 2015, Niemann et al^15^ reported that therapeutic hypothermia (34-35 °C) compared with normothermia (36.5-37.5 °C) in brain dead kidney donors improved kidney allograft outcomes with a significantly lower rate of DGF among kidney transplant recipients assigned to hypothermia vs normothermia (28% vs 39%, odds ratio [OR], 0.62; 95% CI, 0.43-0.92; *P* = .02). This benefit was particularly prominent in kidney grafts from higher risk ECDs. Due to the early termination of the trial at 49% enrollment, further subgroup analyses were limited, leaving unanswered the question of a potential benefit of donor-therapeutic hypothermia in lower-risk SCDs.

More recently, Malinoski et al^16^ reported the results of a follow-up prospective randomized trial in which only pump-eligible donors were enrolled, assessing the role of ex situ hypothermic machine perfusion of the kidney allograft in the context of mild therapeutic hypothermia of the brain-dead donor during organ donor management. Pump eligibility was determined via Organ Procurement Organization (OPO) criteria, whereby some OPOs pumped kidneys with inferior organ quality (ECD status or high KDPI) or anticipated long cold ischemia time. Non–pump-eligible organs were excluded from this study as they were preserved via static cold storage. This trial found that, among brain-dead organ donors, therapeutic hypothermia was inferior to ex situ kidney hypothermic machine perfusion in reducing DGF after kidney transplant, with DGF occurring in 30% of patients in the hypothermia group vs 19% in the machine-perfusion group (adjusted risk ratio of DGF, 1.72; 95% CI, 1.35-2.17). Additionally, the combination of hypothermia and machine perfusion did not provide added protection.

The present study aimed to determine whether hypothermia is superior to normothermia in preventing DGF in kidneys that did not receive ex situ hypothermic machine perfusion per OPO protocol, as these donors were most often of lowest risk.

## Methods

### Study Design

A pragmatic (designed to evaluate interventions in routine practice conditions), adaptive, multisite prospective randomized clinical trial was conducted between August 10, 2017, and May 21, 2020, across 7 states (Arizona, Colorado, Minnesota, North Dakota, Oregon, South Dakota, and Texas) and 6 OPOs. In this overarching trial,^16^ there were 2 donor populations (pump eligible and non–pump eligible) and enrollment was based on each OPO’s current pumping criteria. This study used data from the Organ Procurement and Transplantation Network (OPTN). The OPTN data system includes data on all donor, wait-listed candidates, and transplant recipients in the US, submitted by the members of the OPTN. The Health Resources and Services Administration (HRSA) of the US Department of Health and Human Services provides oversight to the activities of the OPTN contractor. The first study focused on the pump-eligible donor population and aimed to determine the relative effect of targeted mild hypothermia in the donor vs ex vivo machine perfusion of kidney grafts on kidney recipient DGF. As mentioned, pump eligibility was determined via OPO criteria, whereby some OPOs pumped kidneys with inferior organ quality (ECD status or high KDPI) or anticipated long cold ischemia time. There were 2 OPOs that intended to pump all kidneys, resulting in 4 OPOs included in the present report. The primary results of the population of pump-eligible donors were recently reported.^16^The study was evaluated by the University of California, San Francisco, Institutional Review Board. It was determined that the enrollment of brain-dead organ donors did not constitute human participants research, and a waiver of informed consent for research on kidney recipients was granted. This report follows the Consolidated Standards of Reporting Trials (CONSORT) reporting guideline for randomized clinical trials.

**Figure 1.**
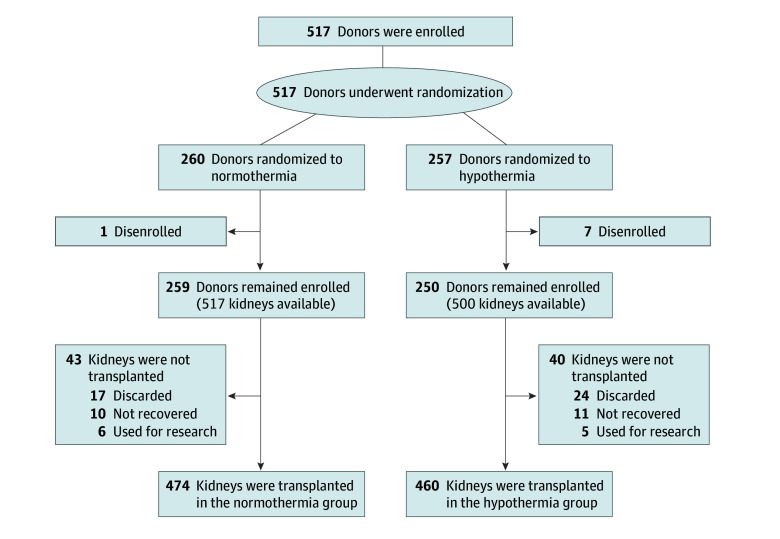
Non–Pump-Eligible Donors

The present study represents the second population of the overarching trial: low-risk non–pump-eligible donors from participating OPOs. In this randomized study, the aim was to determine whether mild hypothermia was superior to normothermia (machine perfusion was not part of the planned interventions). The maximum sample size of 1400 donors for the present study was determined via simulation. The adaptive design report of the trial protocol (Supplement 1) details that a maximum of 1400 donors provided 90% power to detect an absolute improvement of 7.5% in DGF rates for hypothermia vs normothermia and 80% power to detect an absolute improvement of 6% in DGF rates for hypothermia vs normothermia. Due to funding and operational reasons, the trial was stopped before an interim analysis was conducted in the non–pump-eligible population. At the time the overarching trial was stopped, 517 non–pump-eligible donors were enrolled (Figure 1), leading to a total enrollment of 1427 in the overarching trial (including 910 donors from the previously published pump-eligible population), as listed on ClinicalTrials.gov.

### Study Participants

All brain-dead kidney donors aged 18 years and older with a written documented research authorization were considered for enrollment. Once the declaration of death according to neurologic criteria was determined, research authorization was obtained from next of kin. All donors whose kidneys did not require machine perfusion were assigned to donor therapeutic hypothermia (34-35 °C) or normothermia (36.5-37.5 °C) in a 1:1 ratio using computer-generated block randomization (randomize.net). Randomization to hypothermia vs normothermia occurred at the donor level (unit of randomization), and the outcome of DGF was analyzed at the kidney level (unit of analysis). Block randomization was used to ensure balance across all location sites, randomization was stratified by location site/OPO, ECD/SCD status, and prior exposure to hypothermia before brain death. Brain-dead kidney donors were clinically managed by each OPO per their local donor management guidelines, which were in line with the Donor Management Goals. Temperature management followed a trial protocol that was previously reported.^15^ Criteria that made donors pump-eligible, and therefore excluded from the present study, are detailed in Supplement 1.

Data from the trial were merged with routinely obtained Organ Procurement and Transplantation Network data, which allowed for linkage of donor information, graft preservation, and outcome data, as well as recipient risk factor data, which were all deidentified with respect to the recipient and linked to each donor’s sequential OPTN donor identification number. Donor data included age; gender; body mass index; type (ECD vs SCD); KDPI, as determined by the Scientific Registry of Transplant Recipients before hypothermia (ie, before the declaration of death according to neurologic criteria); creatinine level and estimated glomerular filtration rate at enrollment and before procurement; and OPO. Recipient data included age, gender, body mass index, cold ischemic time, hepatitis C virus serologies, human leukocyte antigen mismatches, panel-reactive antibody, donor location (placement within or outside of donor-specific antibodies), time receiving kidney replacement therapy, history of transplant, and occurrence of DGF, which was defined as the need for dialysis within the first week following kidney transplant. Donor and recipient data were linked. The last data of recipient follow-up for 1-year function was June 14, 2021, and the final analysis report is dated June 15, 2022.

### Outcome Measures

The primary end point was DGF, as determined and reported by the center at which the organ was transplanted. The purpose of this randomized trial was to determine whether hypothermia was superior to normothermia in preventing DGF in low-risk nonpumped kidneys from brain-dead donors.

### Statistical Analysis

The primary analysis follows the intent-to-treat principle, meaning that kidneys were analyzed according to their randomized assignment regardless of the treatment received. The primary analysis population was defined as all transplanted kidneys with known DGF outcomes. The primary analysis model is a generalized estimating equation logistic model that accounts for possible correlation between kidneys within a donor by using a compound symmetric correlation structure. Thus, although randomization happened at the donor level, the treatment effect on DGF was analyzed at the kidney level in the generalized estimating equation model. The model included terms for the randomized treatment group (hypothermia vs normothermia), as well as adjustment for the following protocol-prespecified covariates: OPO, donor type (ECD vs SCD), donor creatinine level at enrollment, donor age, and kidney cold ischemic time. Treatment effects are reported in terms of model estimated odds ratio (OR) comparing the randomized treatment groups and the corresponding 95% CI and 2-sided *P* value. The trial design controlled for type 1 error at 5%. A statement of superiority for hypothermia vs normothermia would have been made if the observed *P* value (*P* = .66) had been <.05. Equivalently, because ORs less than 1 represent favorable treatment effects (reduced DGF rates), if the upper limit of the 95% CI for the OR of DGF comparing hypothermia with normothermia had been below 1, the trial would have claimed superiority of hypothermia vs normothermia. The outcome is modeled as either DGF = 1 or DGF = 0. Thus, ORs less than 1 indicate favorable treatment effects, ORs of 1 represent the null effect, and ORs greater than 1 are unfavorable treatment effects. Continuous variables are summarized as mean (SD). Factor variables are summarized as count (percentage). The referent donor type is SCD, and the referent OPO is OPO 34 (numbering of deidentified OPOs was arbitrary). Data analysis was performed using R software, version 4.1.0 (R Foundation for Statistical Computing).

## Results

During the study period, 517 brain-dead organ donors (1033 kidneys) did not require machine perfusion per OPO protocol (ie, were non–pump eligible). Of these, 8 donors were disenrolled (due to meeting exclusion criteria, request by the donor’s family or the accepting organ center, or decision by the principal investigator), leaving 509 donors (1017 kidneys) randomized. Of the 1017 kidneys enrolled, 934 kidneys from 481 donors were transplanted with known DGF outcomes (reasons for nontransplant in the remaining 83 included 51 discarded, 21 not recovered, and 11 research). There were 474 kidneys randomized to the normothermia group and 460 to the hypothermia group (Figure 1). Mean (SD) donor age was 34.2 (11.1) years, and donor creatinine level at enrollment was 1.03 (0.53) mg/dL The non–pump-eligible population of the trial was stopped early due to operational reasons (a slower than anticipated enrollment in the non–pump-eligible arm and the grant funding period ending). As a result, the non–pump-eligible population stopped enrolling before the first interim analysis was conducted (which was planned to be conducted when 600 nonpump donors had known DGF outcomes). Thus, the sample size for the present analysis is much lower than the planned maximum of 1400 donors.

**Figure 2.**
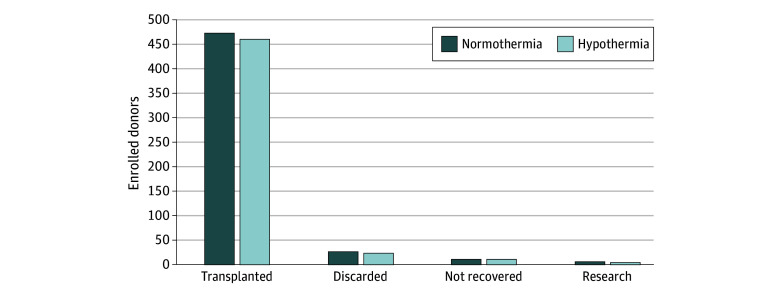
Proportion of Kidneys by Treatment Arm (Enrolled Donors)

Donor characteristics are presented in Table 1 and were similar between groups. These were notable for a predominance of SCD donors (98% in both treatment arms) and similar KDPIs. The proportion of kidneys (by treatment arm) that were transplanted, discarded, not recovered, and used for research is presented in Figure 2. Recipient characteristics are presented in Table 2. Cold ischemia time was similar in the normothermia vs hypothermia group (16 [7.8] vs 15.5 [7.6] hours) as was the nonadjusted rate of DGF (18% vs 17%; OR, 0.91; 95% CI, 0.63-1.31; *P* = .61). When adjusted for prespecified covariates (creatinine level at enrollment, donor age, donor type, cold ischemic time, and OPO), the OR for DGF in the hypothermia vs normothermia group was 0.92 (95% CI, 0.64-1.33; *P* = .66). The results of the primary efficacy analysis are presented in Table 3.

**Table 1.** Characteristics of Donors in the Analysis Population

Characteristic	Overall (N = 481)	Normothermia (n = 245)	Hypothermia (n = 236)
Age, mean (SD), y	34.22 (11.11)	33.92 (10.6)	34.52 (11.62)
Sex, No. (%)			
Female	176 (37)	90 (37)	86 (36)
Male	305 (63)	155 (63)	150 (64)
Height, mean (SD), cm	172.75 (9.75)	172.83 (9.81)	172.67 (9.71)
Weight, mean (SD), kg	82.71 (20.6)	81.19 (20.82)	84.29 (20.28)
BMI, mean (SD)	27.69 (6.5)	27.15 (6.61)	28.24 (6.36)
SCD, No. (%)	472 (98)	240 (98)	232 (98)
ECD, No. (%)	9 (2)	5 (2)	4 (2)
Kidney Donor Profile Index, mean (SD)	28.66 (21.16)	28.99 (20.46)	28.32 (21.9)
Prior hypothermia, No. (%)			
No	385 (80)	202 (82)	183 (78)
Yes	96 (20)	43 (18)	53 (22)
Creatinine at enrollment, mean (SD), mg/dL	1.03 (0.53)	1.07 (0.6)	0.99 (0.43)
Creatinine before procurement, mean (SD), mg/dL	0.96 (0.72)	1.06 (0.87)	0.86 (0.49)
Enrollment eGFR, mean (SD), mL/min/1.73 m^2^	98.82 (32.77)	96.45 (31.51)	101.26 (33.92)
eGFR before surgery, mean (SD), mL/min/1.73 m^2^	107.4 (33.82)	101.19 (32.51)	113.78 (34.03)
OPO, No. (%)			
34	247 (51)	129 (53)	118 (50)
2	102 (21)	51 (21)	51 (22)
29	76 (16)	36 (15)	40 (17)
40	56 (12)	29 (12)	27 (11)

Abbreviations: BMI, body mass index (calculated as weight in kilograms divided by height in meters squared); ECD, Extended Criteria Donor; eGFR, estimated glomerular filtration rate; OPO, Organ Procurement Organization; SCD, Standard Criteria Donor.

SI conversion: To convert creatinine to micromoles per liter, multiply by 88.4.

**Table 2.** Characteristics of Kidney Transplant Recipients

Characteristic	Overall (N = 934)	Normothermia (n = 474)	Hypothermia (n = 460)
Age, mean (SD), y	47.72 (15.33)	47.32 (15.62)	48.13 (15.03)
Sex, No. (%)			
Female	392 (42)	201 (42)	191 (42)
Male	542 (58)	273 (58)	269 (58)
BMI, mean (SD)	28.03 (9.25)	27.79 (7.61)	28.27 (10.69)
Kidney cold ischemic time, mean (SD), h	15.73 (7.77)	15.99 (7.9)	15.45 (7.63)
No DGF	768 (82)	387 (82)	381 (83)
DGF	166 (18)	87 (18)	79 (17)
Height, mean (SD), cm	167.94 (14.73)	167.63 (15.22)	168.25 (14.21)
Weight, mean (SD), kg	79.36 (20.28)	78.66 (19.69)	80.09 (20.87)
Donor-recipient weight ratio, mean (SD)	1.15 (0.67)	1.14 (0.73)	1.16 (0.61)
HCV serostatus, No. (%)			
Positive	36 (4)	18 (4)	18 (4)
Negative	885 (95)	451 (95)	434 (94)
Not done	13 (1)	5 (1)	8 (2)
HLA mismatches, mean (SD)	4.1 (1.53)	4 (1.62)	4.2 (1.43)
PRA, mean (SD), %	26.78 (37.9)	26.14 (37.76)	27.44 (38.08)
PRA≤80%, No. (%)	764 (82)	389 (82)	375 (82)
PRA>80%, No. (%)	170 (18)	85 (18)	85 (18)
Not placed outside original DSA, No. (%)	710 (76)	350 (74)	360 (78)
Placed outside original DSA, No. (%)	224 (24)	124 (26)	100 (22)
Time receiving kidney replacement therapy, mean (SD), d	1489.63 (1217.78)	1487.43 (1213.72)	1491.85 (1223.42)
No prior kidney transplant, No. (%)	820 (88)	414 (87)	406 (88)
Prior kidney transplant, No. (%)	114 (12)	60 (13)	54 (12)

Abbreviations: BMI, body mass index (calculated as weight in kilograms divided by height in meters squared); DGF, delayed graft function; DSA, donor-specific antibodies; HCV, hepatitis C virus; HLA, human leukocyte antigen; PRA, panel-reactive antibody.

**Table 3.** Results of the Primary Analysis of DGF^a^

Variable	AOR for DGF (95% CI)	*P* value
Hypothermia vs normothermia	0.92 (0.64-1.33)	.66
Enrollment creatinine, mg/dL	1.69 (1.19-2.4)	.004
Donor age, y	1.03 (1.02-1.05)	<.001
Donor type: ECD vs SCD	0.11 (0.03-0.45)	.002
Kidney cold ischemia time, h	1.03 (1.01-1.06)	.003
OPO 2 vs 34	2.59 (1.68-3.99)	<.001
OPO 29 vs 34	0.64 (0.33-1.26)	.20
OPO 40 vs 34	0.79 (0.41-1.49)	.46

Abbreviations: AOR, adjusted odds ratio; DGF, delayed graft function; ECD, Extended Criteria Donor; OPO, Organ Procurement Organization; SCD, Standard Criteria Donor.

SI conversion: To convert creatinine to micromoles per liter, multiply by 88.4.

^a^ A generalized estimating equation logistic regression model of DGF in kidney transplant recipients with adjustment for prespecified covariates. The model is parameterized so that AOR less than 1 represents favorable treatment effects (reduced rates of DGF), AOR of 1 indicates the null effect, and AOR greater than 1 represents unfavorable treatment effects (increased rates of DGF). The treatment effect of hypothermia vs normothermia was not significant (*P* = .66 > .05).

## Discussion

Targeted therapeutic hypothermia is an established neuroprotective intervention that entails inducing temperatures between 32 and 35 °C in select patients with stroke or cardiac arrest.^17,18,19^ There has been increasing interest in the use of therapeutic hypothermia during the management of brain-dead donors given initial trial data suggesting decreased DGF in kidney transplant recipients of these organs.^15^ In the present study, the lowest-risk group of donors—those who did not qualify for ex situ hypothermic machine perfusion of kidneys per OPO protocol—were randomized to normothermia vs hypothermia and delayed graft function was found to be similar in both groups (18% vs 17%).

In 2015, Niemann et al^15^ conducted a randomized clinical trial in 2 organ donation service areas to assess the safety and potential benefit of donor hypothermia on outcomes in kidney transplant recipients. On prespecified subgroup analyses assessing the impact of therapeutic hypothermia of ECDs vs SCD, this effect was most notable in the highest-risk ECD group (DGF 31% in the hypothermia vs 56% in the normothermia arm; adjusted OR of DGF in this subgroup, 0.31; 95% CI, 0.15-0.68; *P* = .003). Furthermore, although kidney grafts from SCDs with therapeutic hypothermia had lower DGF than the normothermia group (27% vs 34%), this difference was not statistically significant (adjusted OR, 0.71; 95% CI, 0.45-1.13; *P* = .15). Given early termination of the trial due to overall efficacy on interim analysis, it remained unknown whether this finding in lower-risk donors was potentially attributable to the study being underpowered to assess effect in this subgroup.

More recently, a follow-up prospective randomized trial compared the efficacy of 3 strategies in reducing DGF in pump-eligible donors: donor therapeutic hypothermia (34-35 °C), ex situ hypothermic machine perfusion of the kidney, or their combination.^16^ In this trial, only pump-eligible donors were analyzed, and therapeutic hypothermia was found to be inferior to machine perfusion in reducing DGF after kidney transplant (adjusted risk ratio of DGF, 1.72; 95% CI, 1.35-2.17). In the present report, we thus assessed outcomes of the low-risk nonpumped kidneys from brain-dead donors to assess whether there is the benefit of therapeutic hypothermia in this lower-risk group of donors. We expected to see a similar kidney protective pattern of reduction in the rates of DGF in the hypothermia group. However, we found no statistically significant differences in the rates of DGF on univariate and adjusted analyses. The low-risk nonpumped group was predominantly composed of SCDs (472 [98%]), which by definition represented a younger and healthier subset of donors. Thus, despite its potential benefit in ECDs that are unable to undergo machine perfusion, therapeutic hypothermia does not appear to confer a similar benefit in reducing DGF in nonpumped donors.

### Limitations

This study has some limitations. Specifically, there are currently no standardized pump-eligibility criteria, and therefore criteria were determined locally by each OPO. Two of the 6 OPOs in the overarching trial (including pump-eligible) pumped all organs and therefore did not contribute data to the current non–pump-eligible analysis. Although pump-eligible donors tended to be those with ECD status, high KDPI, or long anticipated cold ischemic time, exact criteria differed across OPOs. However, this was a pragmatic clinical trial intended to generate evidence in the clinical practice setting, and thus was not specifically designed to assess rules of pumping agreed upon by the OPO and transplant centers. Next, cold ischemia time could not be controlled in this type of trial and always will be a confounding covariate. However, the generalized estimating equation logistic model accounts for this by adjusting for OPO, cold ischemia time, and other covariates, but some heterogeneity may still exist. Lastly, a previous trial^15^ reported a difference of DGF rates in SCD donors in the normothermia vs hypothermia groups (27.3% vs 33.6%), although this difference did not reach statistical significance. Although in this follow-up study we did not reach the original planned enrollment target of 1400 donors, our results do not show that a difference in DGF would have been observed in the low-risk non–pump-eligible donor population between the normothermia vs hypothermia rates of DGF (18% vs 17%), which was predominantly composed of SCD donors (472 [98%]).

## Conclusion

The results of this randomized clinical trial suggest that, for OPOs that selectively use ex situ hypothermic machine perfusion for preservation of kidneys from donors after brain death, the use of therapeutic hypothermia during deceased donor management is likely not necessary, as pumping is sufficient for pump-eligible organs^16^ and hypothermia does not add benefit for the lower risk non–pump-eligible group. In contrast, for OPOs that do not have a pumping infrastructure (either in the US or elsewhere worldwide), therapeutic hypothermia of the deceased donor should be considered in donors with high KDPI, as it confers a protective benefit against DGF.^15^

## Notes

Supplement 1.
Trial Protocol

Supplement 2.
Data Sharing Statement
