# Supplementary material for: Therapeutic Hypothermia in Low-Risk Nonpumped Brain-Dead Kidney Donors: A Randomized Clinical Trial
Source: JAMA Netw Open. 2024 Feb 28;7(2):e2353785. doi: 10.1001/jamanetworkopen.2023.53785 (PMC10902731; doi:10.1001/jamanetworkopen.2023.53785)
Supplement: Supplement 1. — Trial Protocol [file jamanetwopen-e2353785-s001.pdf]

Protocol Packet JNO23-8282 – Therapeutic Hypothermia in Low-Risk Non-Pumped Brain-Dead Kidney Donors

This supplement contains the following items:

1. **Protocol**
   Original Protocol(July 26, 2017):Pages 2-45
   Final Protocol (May 19, 2020):Pages 46-93
   Summary of Changes: Pages 94-95

2. **Statistical Analysis Plan**
   Original Statistical Analysis Plan (July 20, 2017): Pages 16-25
   Final Statistical Analysis Plan (February 25, 2020): Pages 96-99
   Summary of Changes: Page 100

DONOR MANAGEMENT RESEARCH INITIATIVE

A Randomized Trial of Mild Hypothermia and Machine Perfusion in Deceased Organ Donors for Protection against Delayed Graft Function in Kidney Transplant Recipients

Principal Investigators
Darren Malinoski, MD, Oregon Health and Science University, 3181 SW Sam Jackson Park Rd. Portland, OR 97239
malinosk@ohsu.edu

Claus Niemann, MD, University of California San Francisco, 500 Parnassus MUE 4th Floor Box 0648, San Francisco, Ca 94143-0648, USA
Claus.Niemann@ucsf.edu

Study and Data Coordinating Center
UNOS Business Services
700 N. 4th St, Richmond, Virginia. 23219

Trial Manager
Alex Garza

Data Analytics Manager
Sarah Taranto

Data Quality
Brooke Chenault

Research Scientist
Robert Carrico

Administrative Oversight
Ryan Ehrensberger

Trials Design and Statistics

Kristine Broglio, M.S.
Berry Consultants, 4301 Westbank Dr #140B, Austin,TX 78746
kristine@berryconsultants.net

Chair, Independent Data Safety and Monitoring Board

Roger J. Lewis, MD, PhD, Department of Emergency Medicine Harbor-UCLA Medical Center, Bldg D9, 1000 W Carson St, Torrance, CA 90509 Torrance, CA, USA

Education

Sharon Swain, RN, MSN, Clinical Nurse Educator, Donor Network West

Consultant

Jenna Graciano, MBA, Transplant Quality Manager, St. Vincent Medical Center – Multi-Organ Transplant Center. 2200 West Third Street #500, Los Angeles, CA 90057, USA. jgraciano@me.com

Trial Coordination Team

Darren Malinoski, MD and Claus Niemann, MD, Alex Garza, BS, Jenna Graciano, MBA, Sharon Swain, RN, MSN, Tahnee Groat, MPH, and one representative from each participating OPO

Organ Procurement Organization Sites

Southwest Transplant Alliance, 5489 Blair Rd, Dallas, TX 75231

Site PI: Jeffrey Reese, MD

Site Study Coordinator: Ryan Mulcahy

Life Gift of Houston, 2510 Westridge St, Houston, TX 77054

Site PI: TBD

Site Study Coordinator: TBD

Additional sites to be determined

Funding

This trial is supported by a grant from the Laura and John Arnold Foundation, Houston, Texas.

Abbreviations:

DGF – Delayed Graft Function
DMG – Donor Management Goals
DNDD – Donor after Neurologic Determination of Death
DSA – Donation Service Area
DSMB – Data Safety Monitoring Board
ECD – Expanded Criteria Donor
MP – Machine Perfusion
OPO – Organ Procurement Organization
RCT – Randomized Controlled Trial

Table of Contents

Section	Topic	Pages
I.	Cover Pages	2-3
II.	Abbreviations	4
III.	Table of Contents	5
IV.	Randomized Control Trial Summary	6-7
V.	Protocol	8-39
	a. Background	8-9
	b. Approach	10-12
	c. Targeted Temperature Management	13-14
	d. Target Population and Setting	15
	e. Adaptive Trial Design Report/Statistical Analysis	16-25
	f. Other General Statistical Considerations	26-27
	g. Education	28-35
	h. Communication	36
	i. Data Collection	37-38
	j. Trial Timeline	39
VI.	Appendix A. Full Study Algorithm- Data Collection Sheet	40-42
VII.	Appendix B. IRB Outcome Letter	43-44
VIII.	Acknowledgments	45

Randomized Control Trial Summary - Page 1

- **"A Randomized Trial of Mild Hypothermia and Machine Perfusion in Deceased Organ Donors for Protection against Delayed Graft Function in Kidney Transplant Recipients"**
- **The Regents of University of California, San Francisco, and Oregon Health & Science University**
- **PI: Darren Malinoski, MD, FACS - malinosk@ohsu.edu, (503) 701-7628**
- **PI: Claus Niemann, MD - Claus.Niemann@ucsf.edu, (415) 502-2162**

BACKGROUND: In the initial Mild Hypothermia Randomized Control Trial (RCT), in collaboration with the UNOS Region 5 Donor Management Goals (DMG) Workgroup and Web Portal, the research team was able to conduct a multi-center RCT examining the benefits of mild hypothermia in donors after neurologic determination of death (DNDDs) on the outcomes of kidney transplantation. The trial was stopped early by the DSMB due to a significant positive benefit to kidney transplant recipients, including a 38% reduction in the odds of delayed graft function (DGF, the primary outcome measure of the trial). The results of this study have been published in the *New England Journal of Medicine* (July 2015). This research offers a zero-cost intervention that can substantially increase transplant success as well as the pool of potential donors.

To expand upon the success of the hypothermia study, the team is conducting a new RCT to test whether hypothermia is as effective as machine perfusion (MP) of kidneys from DNDDs. In an RCT conducted by the Eurotransplant International Foundation in 2009 (Moers et al. *NEJM*), the protective effect of MP (OR = 0.57) was similar to that found in our trial (OR = 0.62). However, the cost of MP can be very significant for organ procurement organizations (OPOs) and transplant centers.

MP of kidneys from deceased donors has been increasingly adopted by many centers even though clinical and cost effectiveness studies remain uncertain in the United States. Between 2012 and 2014, out of 31,798 kidneys available for transplant, 11,998 (38%) of them were machine perfused. Over the same three-year period, the number of kidneys pumped annually increased by over 20%.

This is an opportune time to investigate the effectiveness of MP compared to mild hypothermia, as there are enough OPOs currently using MP that if mild hypothermia was found to be a non-inferior intervention, there would be considerable cost savings. Similarly, over 60% of kidneys do not receive machine perfusion and findings that demonstrates a benefit of machine perfusion would likely lead to rapid increase in use. In addition, DGF still occurs in up to 56% of high-risk kidneys despite using one of these protective measures and their combined use may be the best approach moving forward. Either way, a new evidence-based standard will be created that will significantly affect the way kidney transplants are handled.

METHODS: This will be a pragmatic multi-site randomized controlled trial that bases enrollment on each OPO/Donation Service Area's current pumping criteria **(Figure 1**, page 2**).** There will be two main groups of DNDDs, (1) those that are "*pump eligible*" based on current practice (this group typically resembles traditional expanded criteria donors, but is increased in some areas) and (2) those that are lower risk and whose kidneys do not receive MP ("*not pump eligible*"). Kidneys from donors who are considered "pump eligible" currently receive MP based on their increased risk for failure. In this trial, "pump eligible" DNDDs will be randomized to one of three groups (**Figure 2**): (1) normothermia (36.5-37.5 C) plus MP of both kidneys (standard of practice control group), (2) mild hypothermia (34-35 C) plus MP of the left kidney only, and (3) mild hypothermia plus MP of the right kidney only. In this manner, the same number of kidneys will be randomized to each of the three treatment strategies (MP alone, mild hypothermia alone, or MP+hypothermia). **It is important to note that kidneys from "pump eligible"/higher risk DNDDs will still receive one form of protection and possibly two.**

In contrast, "not pump eligible" DNDDs will only be randomized to one of two groups: (1) therapeutic mild hypothermia or (2) normothermia. Being that our first trial was stopped early for efficacy in the overall DNDD population, there was insufficient statistical power to confirm a benefit in standard criteria donors (p=0.1 at stoppage). The purpose of this arm of the trial is to validate the protective effect of hypothermia in a larger sample size of lower-risk / "not pump eligible" donors.

The following objectives will be addressed by the trial:

- Determine the non-inferiority of a hypothermia-only strategy to a standard pump-only strategy in high risk DNDDs
- Evaluate the superiority of a combined hypothermia+MP strategy to both hypothermia or MP alone in high risk DNDDs
- Evaluate the superiority of mild hypothermia versus standard of care normothermia in lower risk, "not pump eligible" DNDDs
- Determine the safety of the hypothermia strategy with respect to the function of "bystander" organs (e.g., heart, lung)

This protocol has been approved by the UNOS Region 5 Research Committee. In addition, the following steps have been or will be taken:

- A National communication was sent via TransplantPro to allow for a two-week period for public comment.
- Only donors whose families and/or advanced directives (donor registry) have authorized research will be included in the study.
- All organ offers from DNDDs enrolled in the study will include a message in the "Donor Highlights" section of DonorNet, a copy of the study summary will be attached to the record, and allocation/transplantation will occur based on standard practice.
- There will not be any interaction between the study team and the transplant recipients and no additional data will be collected.
- Recipient graft function data will be derived from standard UNet forms and obtained from the OPTN in a de-identified format.

- **"A Randomized Trial of Mild Hypothermia and Machine Perfusion in Deceased Organ Donors for Protection against Delayed Graft Function in Kidney Transplant Recipients"**
- **The Regents of University of California, San Francisco, and Oregon Health & Science University**
- **PI: Darren Malinoski, MD, FACS – malinosk@ohsu.edu, (503) 701-7628**
- **PI: Claus Niemann, MD – Claus.Niemann@ucsf.edu, (415) 502-2162**

Figure 1 – Participating OPO "Pump Eligible" Criteria

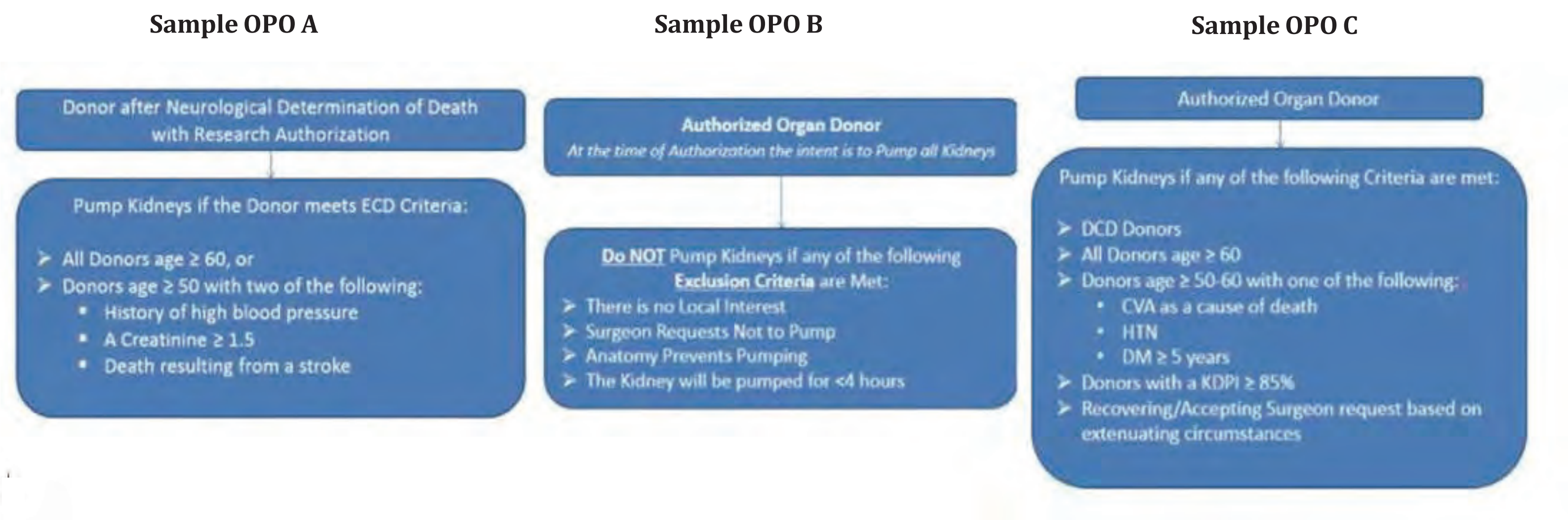

Figure 2 – Randomization Scheme

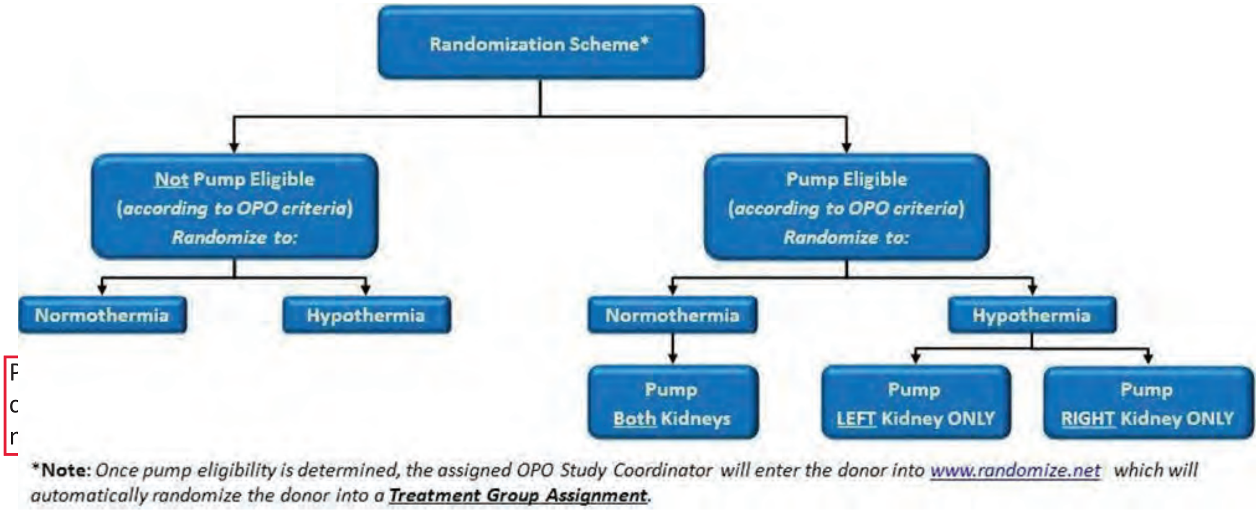

Background

Initial Hypothermia RCT

Utilizing the Donor Management Goal (DMG) Working Group and Web Portal, the research team was able to conduct a multi-center Randomized Control Trial (RCT) examining the benefits of therapeutic mild hypothermia (34° – 35° C) in organ donors after neurologic determination of death (DNDDs) on the outcomes of kidney transplantation. The trial was stopped early due to a significant positive benefit to kidney transplant recipients, including a 38% reduction in the odds of delayed graft function (DGF, the primary outcome measure). The results of this trial have been published in the *New England Journal of Medicine* (N Engl J Med. 2015 Jul 30;373(5):405-14) and are likely to significantly change the standard of care for deceased donor management around the world. In fact, the Executive Director of the Association of Organ Procurement Organizations has already hosted a national webinar to foster adoption of mild hypothermia as a part of standard deceased donor management protocols. This research offers a zero-cost intervention that can substantially increase transplant success and the pool of potential donors for decades to come.

This initial, successful study demonstrates the vast amount of unrealized potential that rigorously conducted donor intervention experiments can have on saving and enhancing lives through improved, evidence-based standards of donor management. In addition to being a scientifically successful study, it was an even bigger logistical accomplishment in that it covered a large geographic area, included all three phases of care through which an organ donor passes (donor hospital, organ procurement organization [OPO], and transplant center), and coordinated communications and oversight at the local, regional, and national levels. The unique collaboration and infrastructure developed to complete this study is poised to expand to additional regions and conduct the rigorous trials needed to advance the science in this field.

New Proposed Study: Hypothermia vs. Machine Perfusion of Kidneys

To expand upon the success of the hypothermia study, the team is prepared to conduct a new RCT to test whether hypothermia is as effective as machine perfusion of kidneys in preventing DGF. Machine perfusion is often used as an alternative to cold storage of kidneys during the period between organ removal and transplantation. With machine perfusion, the kidney is connected to a perfusion device, and a solution is pumped continuously through the vasculature. The cost of machine perfusion can be significant. The capital cost per machine can range from $25,000-$30,000 and each use can cost an additional $4,000-$6,000 in disposables (e.g. reagent solutions and single-use disposable cartridges) per kidney (not including labor costs).

Machine perfusion of kidneys from deceased donors has been rapidly adopted in the United States, despite the fact that clinical and cost effectiveness studies remain uncertain. Between 2012 and 2014, out of 31,798 kidneys available for transplant, 11,998 (38%) of them were machine perfused. Over the same three-year time period, the number of kidneys pumped annually increased by over 20%.

This is a key time to investigate the effectiveness of machine perfusion compared to mild hypothermia, as there are enough OPOs currently using machine perfusion that if mild hypothermia was found to be a non-inferior intervention, there would be considerable cost savings. Similarly, over 60% of kidneys do not receive machine perfusion and findings that demonstrate a benefit of machine perfusion would likely lead to rapid increase in use. Either way, a new evidence-based standard will be created that will significantly affect the way kidney transplants are handled.

Approach

Trial Overview – Randomization Scheme

This will be a pragmatic multi-site randomized controlled trial that bases enrollment on each OPO/Donation Service Area's (DSA) current pumping criteria (**Figure 1**)**.** There will be two main groups of DNDDs, (1) those that are "*pump eligible*" based on current practice (this group typically resembles traditional expanded criteria donors [ECDs], but varies across each DSA) and (2) those that are lower risk and whose kidneys do not currently receive MP ("*not pump eligible*").

Figure 1 – Participating OPO "Pump Eligible" Criteria

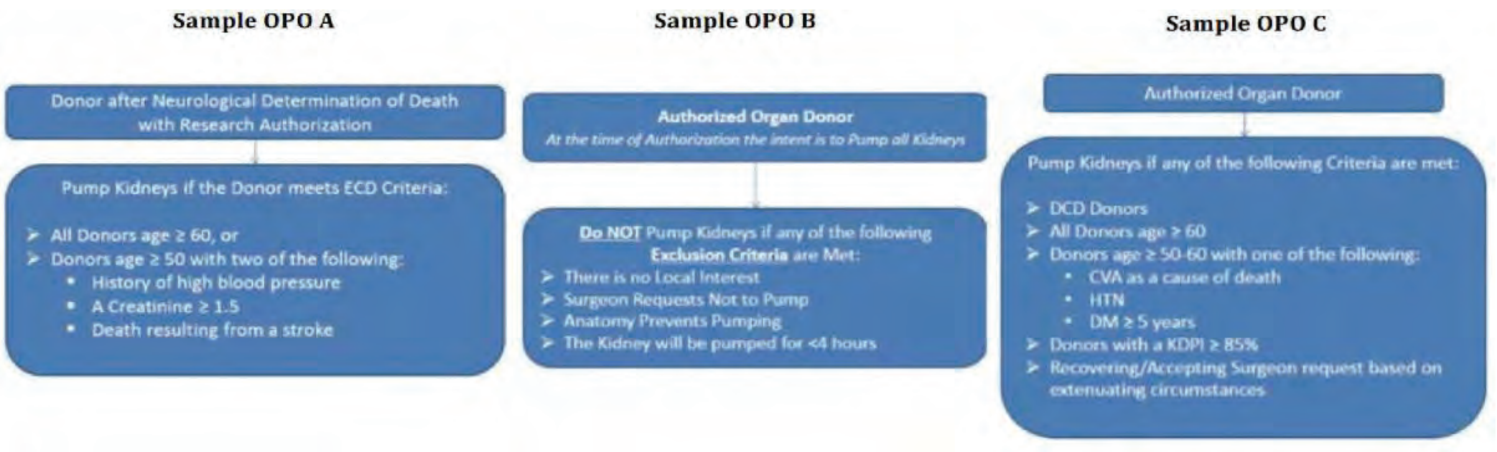

Kidneys from donors who are considered "pump eligible" currently receive MP based on their increased risk for failure. In this trial, "pump eligible" DNDDs will be randomized to one of three groups: (1) normothermia (36.5°-37.5° C) plus MP of both kidneys (standard of practice control group), (2) mild hypothermia (34°-35° C) plus MP of the left kidney only, and (3) mild hypothermia plus MP of the right kidney only (**Figure 2**). In this manner, the same number of kidneys will be randomized to each of the three treatment strategies (MP alone, mild hypothermia alone, or MP + hypothermia). It is important to note that kidneys from "pump eligible"/higher risk DNDDs will still receive one form of protection and possibly two.

In contrast, "not pump eligible" DNDDs will only be randomized to one of two groups: (1)

therapeutic mild hypothermia or (2) normothermia. Being that our first trial was stopped early for efficacy in the overall DNDD population, there was insufficient statistical power to confirm a benefit in standard criteria donors (p=0.1 at the time of trial stoppage). The purpose of this arm of the trial is to validate the protective effect of hypothermia in a larger sample size of lower-risk / “not pump eligible” donors.

Figure 2 – Randomization Scheme

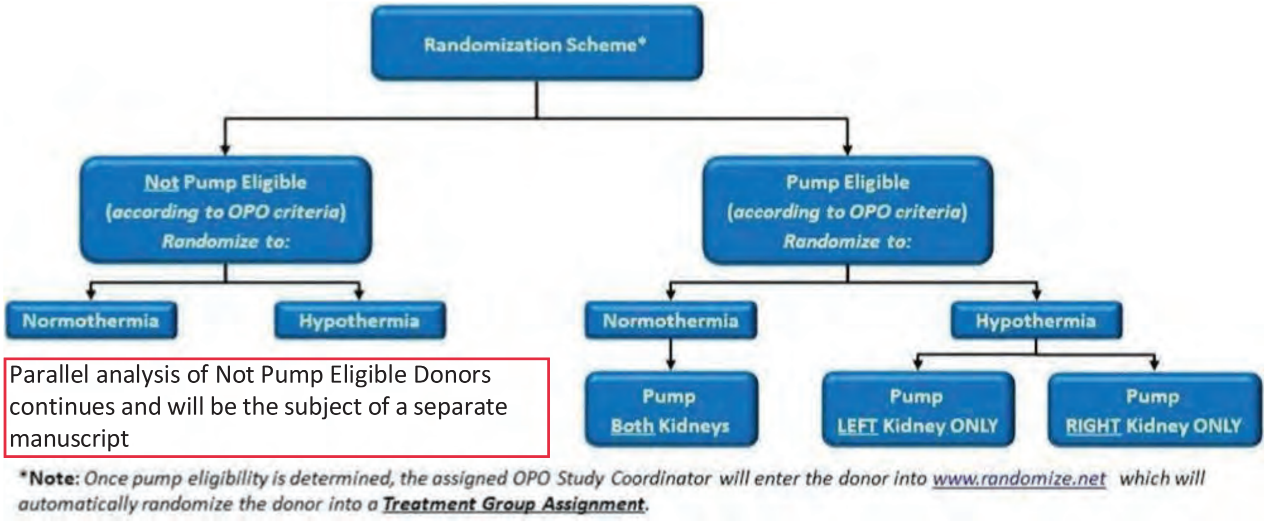

Objective

This randomized clinical trial will evaluate two strategies for improving the post-transplant function of kidneys procured from deceased donors, mild hypothermia applied to the donor before organ procurement and the use of machine perfusion of the kidney after removal from the donor.

The following objectives will be addressed by the trial:

- Determine the non-inferiority of a hypothermia-only strategy to a standard, machine perfusion (MP)-only strategy in high risk, “pump-eligible” DNDDs
- Evaluate the superiority of a combined hypothermia+MP strategy to either hypothermia or MP alone in high risk DNDDs
- Evaluate the superiority of mild hypothermia versus standard of care normothermia in lower risk, "not pump eligible" DNDDs
- Determine the safety of the hypothermia strategy with respect to the function of “bystander” organs (e.g., heart, lung)

Targeted Temperature Management

Mild Hypothermia

Mild hypothermia will be achieved based on the successful protocol used in the previous trial. A detailed description of the protocol is provided below. Importantly, the described protocol is entirely non-invasive, associated with minimal to no cost, and safe when adhered to the protocol. The protocol will only be implemented after DNDDs are deemed stable after a 12-hour initial assessment period. The full study algorithm is depicted in **Appendix A**.

Criteria for initiation of mild hypothermia:

- Organ donors after neurologic determination of death (DNDDs) with research authorization
- There will be no gender or ethnic restrictions
- 18 years of age or older
- Hemodynamic stability – low dose vasopressors and MAP >60 mmHg for more than one hour without an increase in vasopressor dosages. Low-dose is defined as ≤ 1 vasopressor and below the following dosages:
    - Dopamine ≤ 10mcg/kg/min
    - Norepinephrine ≤ 0.2 mcg/kg/min
    - Neosynephrine ≤ 1 mcg/kg/min
    - Epinephrine – any dose is considered out of range
    - Vasopressin – not considered in pressor range
- Corrected coagulopathy
    - INR ≤ 2.5
    - PTT ≤ 3 x normal value
    - Platelet ≥ 50,000
- Corrected electrolytes & maintained per donor management protocol
    - K+ > 3.5 mmol/L
    - Mg++ ≥2.0 mg/dL

Absolute exclusion criteria

- DCD donors
- Dual Kidney Allocation
- Under 18 years of age
- Donors with ESRD (end stage renal disease)
- Dialysis during the terminal hospitalization
- Chronic medical condition precluding general acceptance for transplantation (i.e. cancer, diabetic or hypertensive nephropathy with unacceptable kidney biopsy findings)

Target range 34°-35°C for 12 hours minimum prior to leaving the ICU for organ recovery

1. Attempt to reach target temperature within 4 hours of initiation
2. Non-invasive devices only (invasive methods were not evaluated and should not be utilized)
    3. Cooling (water) blanket
    4. Place under the donor for best effect. When target temperature reached place a blanket or sheet over the top while maintaining the cooling blanket
    5. Precise temperature management system (non-invasive such as Arctic Sun or other similar device)
    6. Bair Hugger or other air device on ambient setting
    7. Ice packs
    8. Ambient

Monitor and document core temperature Q1H by following methods:

9. Bladder temperature probe
10. Rectal temperature probe
11. Pulmonary artery catheter sensor
12. Esophageal temperature probe

Management protocols if procedures or organ recovery requiring travel are initiated (CT scan, angiography, recovery, etc.)

13. If already at target temperature place warm blankets over body and head for transport and attempt to keep covered during procedure
14. Monitor temperature if possible
15. If angiography or other prolonged procedures consider a warming device under the donor, place Bair Hugger (or other air warming device) over lower extremities and warm blanket over head in attempt to maintain target temperature

Specific intra-operative management goals in the hypothermia group
(Goal is to keep temp above 34°C prior to cross-clamp to prevent complications such as arrhythmias)

16. Monitor temp in OR
17. Warming blanket under donor as exposure can lead to more heat loss than desired(turned up to ~37ºC)
18. Bair Hugger on lower extremities (turned up to highest setting)
19. Warm blanket covering head
20. Increase room temperature

Target Population and Setting

Target Population

All DNDDs who have research authorization and meet the inclusion criteria are eligible. Authorization for research will be obtained by a procurement coordinator from each OPO if it is not already present within an advanced directive / state donor registry.

Setting

The proposal and the intervention will be implemented in all organ donor hospitals that are covered by participating OPOs. After declaration of death according to neurologic criteria and obtaining authorization for organ donation, care is transferred to the procurement coordinator. After a 12-hour assessment period, the intervention will be activated based on the inclusion criteria and randomization scheme. Point persons from each OPO are responsible for identifying eligible DNDDs, compliance with the authorization process, as well as implementation and execution of the protocol. In addition, the OPO point person will be responsible for being available for protocol questions that may arise from other OPO Coordinators within their own organization. This approach was highly successful in the previous trial.

Berry Consultants
Statistical Innovation

Adaptive Design Report for the *Randomized Trial of Mild Hypothermia and Machine Perfusion in Deceased Organ Donors for Protection against Delayed Graft Function in Kidney Transplant Recipients*

Submitted in support of the Donor Management Research Initiative
July 20, 2017

1.0 Introduction

This document describes the statistical clinical trial designs for the evaluation of interventions for the prevention of delayed graft function (DGF) in kidney transplant recipients. Two deceased organ donor populations will be studied – those who are eligible for machine perfusion ("pump") of their kidneys and those who are not.

Each population will be considered separately. In the pump-eligible population, we will evaluate both hypothermia and pump. Only hypothermia will be studied in those donors not pump eligible. There will be interim analyses within each population to stop arms early if they are shown to be statistically inferior to the other arms in the population. This document is intended to provide details on the design, primary endpoint, primary analyses, interim analyses, and to present the operating characteristics of the trial for each organ donor population.

2.0 Populations

Donors will be enrolled into the trial and identified as being in one of two populations

Population A: Not Pump Eligible

Population B: Pump Eligible

3.0 Interventions and Randomization

Randomization will be performed separately within each of the two populations.

3.1 Population A: Not Pump Eligible

Donors will be randomized 1:1 to receive either normothermia or hypothermia.

3.2 Population B: Pump Eligible:

Kidneys will be randomized such that kidneys are allocated 1:1:1 to pump alone, hypothermia alone, or the combination of pump and hypothermia. Because hypothermia is performed at the donor level and pump is performed at the kidney level,

donors will be randomized to pump alone, to hypothermia with only the right kidney to be pumped, or to hypothermia with only the left kidney to be pumped.

4.0 Primary Objective

4.1 Population A: Not Pump Eligible

The primary objective is to determine if hypothermia is superior to normothermia in preventing DGF in kidney recipients.

4.2 Population B: Pump Eligible

The primary objective is to determine if hypothermia alone is non-inferior to pump alone in the prevention of DGF in kidney recipients. A key secondary objective is to determine if the combination of pump and hypothermia is superior to the individual therapies in the prevention of DGF in kidney recipients. This trial will ultimately elucidate the comparative-effectiveness of the three intervention arms.

5.0 Primary Endpoint

The primary endpoint in both populations will be delayed graft function (DGF).

6.0 Primary Analysis

The primary analysis method will be a GEE model in order to take into account the correlated nature of kidneys within a donor. The primary efficacy analysis will be based on the GEE model-estimated treatment effects and confidence intervals. The model will be fit for each population separately.

6.1 Population A: Not Pump Eligible

The primary analysis will be based on a GEE model fit including all kidneys with complete DGF information and a term for treatment groups categorized hypothermia versus normothermia. The full primary analysis model will include additional terms to adjust for covariates known to be associated with DGF. This includes terms for organ procurement agency, standard criteria donor vs expanded criteria donor, creatinine at enrollment, donor age, and kidney cold ischemic time. The GEE will use a compound symmetric correlation structure. Treatment effects will be reported in terms of the model estimated odds ratio comparing the two randomized treatment groups, and corresponding 95% confidence interval and two-sided p-value. A statement of superiority will be made based on a comparison of the observed p-value to the trial's nominal p-value required for success.

6.2 Population B: Pump Eligible

The primary analyses will be based on a GEE model fit including all concurrently enrolled kidneys with complete DGF information and a term for treatment group categorized as hypothermia versus normothermia versus the combination. The full

primary analysis model will include additional terms to adjust for covariates known to be associated with DGF. This includes terms for organ procurement agency, standard criteria donor vs expanded criteria donor, creatinine at enrollment, donor age, and kidney cold ischemic time. The GEE will use a compound symmetric correlation structure. Treatment effects will be reported in terms of the model estimated odds ratio comparing two randomized treatment groups, the corresponding 95% confidence interval and two-sided p-value.

We will report final model results for all pairwise comparisons

- Hypothermia versus Pump
- Hypothermia versus Combination
- Pump versus Combination

A statement of superiority will be made based on a comparison of the observed p-value to the trial's nominal p-value required for superiority. Holm's adjustment will be used to account for multiple statistical tests. If the pump and the hypothermia arms are not dropped, a statement of non-inferiority will be made based on a comparison of the final 95% confidence interval to the non-inferiority margin of 1.4.

7.0 Population for Analysis

The final analysis for each comparison of interest will only include data from concurrently randomized kidneys. This applies to only the pump eligible population. If an arm is dropped and additional kidneys are enrolled to the other arms, those additional kidneys will not be included in the analysis model for inference on the dropped arm.

The primary analysis for both populations will follow the intent-to-treat principle meaning that kidneys will be considered according to their randomized assignment regardless of the treatment received.

8.0 Interim Analyses

In each population, there will be 4 interim analyses to drop an arm for inferiority. If only one arm remains, the trial in that population will stop early.

8.1 Population A: Not Pump Eligible

There will be 4 interim analyses during the conduct of the trial to monitor for the inferiority of one treatment arm versus the other (i.e. two-sided monitoring). Interim analyses will be conducted according to an O'Brien-Fleming group sequential stopping boundary. If one of the arms is found to be statistically inferior to the other, enrollment in this population will stop.

A maximum of 1400 donors will be enrolled in this population. Interim analyses are planned beginning when 600 donors have complete DGF outcome information and each

subsequent look is planned after every additional 200 donors have complete DGF outcome information.

8.2 Population B: Pump Eligible

There will be 4 interim analyses during the conduct of the trial to monitor for the inferiority of one or two arms. Interim analyses will be conducted according to an O'Brien-Fleming group sequential stopping boundary. An arm may be dropped if it is found to be inferior to the other arms open and enrolling in the population. Enrollment would continue in the remaining arm(s). Therefore, if all arms are open and being enrolled to, an arm must be statistically inferior to both other arms to be dropped. If an arm has already been dropped, then if an arm is inferior to the only other arm remaining in the trial, it will be dropped. If two arms are dropped, enrollment in this population would end.

A maximum of 1400 donors will be enrolled in this population. Interim analyses are planned beginning when 600 donors have complete DGF outcome information and each subsequent look is planned after every additional 200 donors have complete DGF outcome information.

8.3 *Stopping Boundary*

Both populations have planned interim analyses occurring at the same information fraction. Therefore, both populations have the same O'Brien-Fleming boundary. The nominal two-sided p-values required to drop an arm for inferiority at each interim analysis are provided in the table below.

Table 1: O'Brien Fleming Stopping Boundary

Look<br>(# of Donors Complete)	Nominal two-sided p-value
600	0.0028
800	0.0087
1000	0.0174
1200	0.0276
Final Analysis	0.0386

8.4 Adjustment for Multiple Comparisons

In the pump eligible population, to account for the three pairwise comparisons between the three arms, dropping an arm for inferiority at each interim will be determined according to the following sequence:

1. Determine the global significance of treatment based on an ANOVA p-value between a full and reduced GEE model where the only difference between the full and reduced model is the inclusion or exclusion of the terms for treatment group.

2. Compare the ANOVA p-value to the O'Brien-Fleming nominal two-sided p-values required for stopping at each look (Table 1). If the boundary is crossed, then conduct the pairwise comparisons.

a. The p-values for each of the three pairwise comparisons will be adjusted according to Holm's procedure to account for the multiple statistical tests

b. Compare the Holm's adjusted p-values to the boundary.

c. If one arm crosses the boundary for inferiority versus both of the other arms open and enrolling in the trial, that arm will be dropped

Once an arm is dropped, there is only one comparison left in the population and so the above described approach reduces to a direct comparison between the unadjusted p-value for the single comparison left in the trial to the boundary.

9.0 Operating Characteristics

9.1 Population A: Not Pump Eligible

For the purpose of characterizing the trial, we assume that the DGF rate in the normothermia arm will be 30% and that the correlation between kidneys is 0.15. We assume 30% of all kidneys will not be transplanted and, therefore, will not be included in the analyses. A maximum of 1400 donors provides 90% power to detect an absolute improvement of 7.5% in DGF rates for hypothermia. Similarly, there is 80% power to detect an absolute improvement of 6%. The O'Brien-Fleming boundary controls the overall two-sided Type I error rate at 5%. Assuming an improvement of 7.5% in DGF rates for hypothermia, there is an 83% probability that enrollment in this population would stop early and the mean number of donors is 960. The absolute smallest difference between normothermia and hypothermia expected to be found to be statistical significant is approximately 4%.

9.2 Population B: Pump Eligible

In order to characterize the power and Type I error of the trial design in this population, we simulated the trial under varying scenarios for the delayed graft function (DGF) rate across the three arms; pump alone ("Pump"), hypothermia alone ("Hypo") and hypothermia in combination with pump ("Combo"). These scenarios allow us to assess the power and type I error for both the primary objective of non-inferiority between hypo and pump as well as the secondary objective of superiority between combo and the other arms.

We assume that the underlying DGF rate is 30% and that the correlation between the two kidneys from the same donor is 0.15. We assume that on average 30% of all kidneys will not be transplanted and, therefore, will not be included in the analyses.

We assess power for the non-inferiority comparison assuming that hypothermia and pump have the same underlying DGF rate of 30%. We assess Type I error for the non-

inferiority comparison assuming that one arm is at the non-inferiority margin. Given an underlying DGF rate of 30% and a NI margin on the odds ratio scale of 1.4, this translates to a DGF rate of 37.5%.

We assess power for the superiority comparison assuming a 7.5% improvement in DGF rate for the combination, from 30% to 22.5%. We assess Type I error for the superiority comparison in scenarios assuming the combination arm has the same DGF rate as another arm.

Results are presented for each simulation scenario. Table 2 shows the assumed true underlying DGF rates for each arm in each of the four scenarios. Table 3 shows the average number of donors, the average number of kidneys randomized to each of the three arms of the trial, and the probability of stopping an arm early due to inferiority. Table 4 shows the probability of declaring non-inferiority of hypothermia relative to pump, the probability of declaring superiority for each of the three comparisons, the probability of declaring the combination superior to both other arms, and the probability of declaring at least one of the three comparisons significant.

Table 2: **Simulation Scenarios**

Intervention Arm	Delayed Graft Function (DGF) Scenarios			
	All Same	Combo Best	Hypo Worst	Mixed
Pump	30%	30%	30%	30%
Hypo	30%	30%	37.5%	37.5%
Combo	30%	22.5%	30%	22.5%

Table 3: **Average Sample Size and Probability of Stopping Early for Inferiority**

	Scenario			
	All Same	Combo Best	Hypo Worst	Mixed
Number of Donors: Total	1400	1398.3	1398.9	1191.7
Average Number of Donors: Normothermia	468	466.9	495.9	430.8
Average Number of Donors: Hypothermia	932	931.4	903.0	760.9
Average Number Kidneys: Pump	936	933.9	991.8	861.7
Average Number Kidneys: Hypo	932	930.1	817.4	662.0
Average Number Kidneys: Combo	932.1	932.7	988.6	859.7
Prob. Of Stopping Early for Inferiority: Pump	0.001	0.013	0.002	0.621
Prob. Of Stopping Early for Inferiority: Hypo	0.001	0.013	0.440	0.823
Prob. Of Stopping Early for Inferiority: Combo	0.001	0	0.003	0

Table 4: Power and Type I Error

	Scenario			
	All Same	Combo Best	Hypo Worst	Mixed
Non-inferiority: Hypo vs Pump	0.863	0.862	0.049	0.013
Superiority: Combo vs Hypo	0.015	0.802	0.748	1
Superiority: Combo vs Pump	0.012	0.738	0.029	0.893
Superiority: Hypo vs Pump	0.013	0.034	0.683	0.912
Superiority: Combo vs Both Hypo and Pump	0.001	0.658	0.013	0.792
At Least One Arm Superior to Another	0.032	0.877	0.832	1

The maximum sample size of 1400 donors provides 86% power for the primary comparison of interest, the non-inferiority of pump and hypothermia. For the secondary objective of establishing the superiority of the combination, this trial has 73.8% to 100% power to detect an improvement relative to each of the other arms. There is 65.8% to 79.2% power to detect an improvement for the combination arm versus both the other arms in the trial. For both the non-inferiority and the superiority hypothesis, the overall two-sided Type I error rates are controlled at the 5% level.

We also explore the observed absolute differences between arms that result in either non-inferiority or superiority. Figures 1-3 show the simulation results, plotting the observed data and trial outcome. The plots pool results across the above simulation scenarios. Across all simulated trials, the smallest difference between combination and either pump alone or hypothermia alone expected to be found to be statistical significant is approximately 4.2%. The largest difference between hypothermia alone and pump alone expected to result in statistical non-inferiority is approximately 3.6%

Berry Consultants
Statistical Innovation

Figure 1: Observed Data Resulting in Non-inferiority

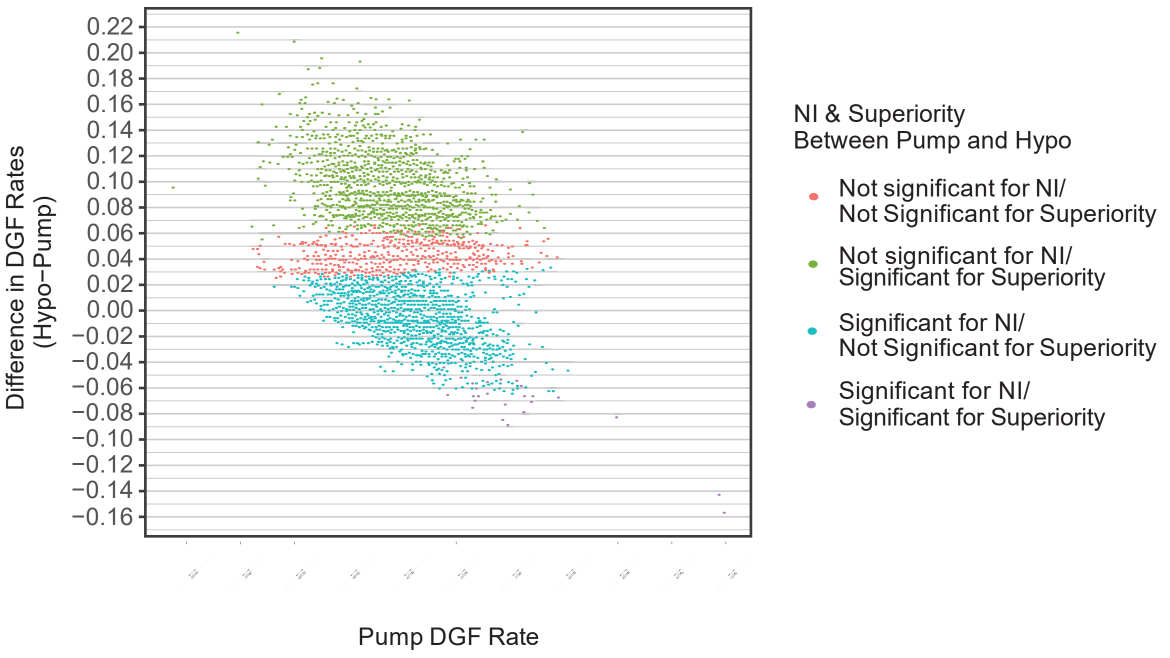

Figure 2: Observed Data Resulting in Superiority of Combo vs Hypo

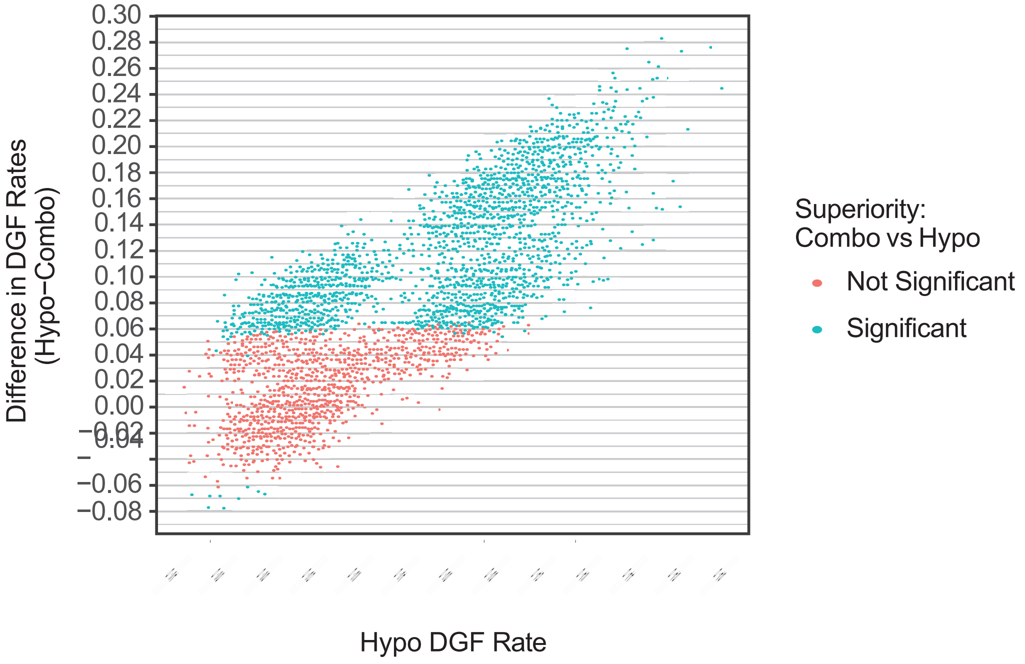

Figure 3: Observed Data Resulting in Superiority of Combo vs Pump

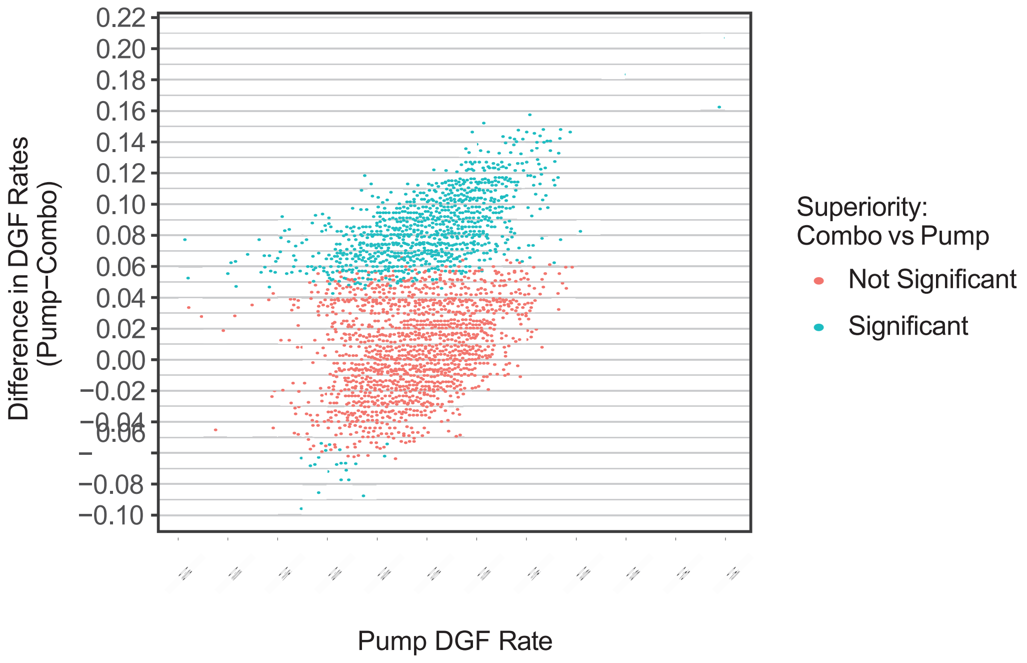

9.4 Additional Simulation Scenarios for Population B: Pump Eligible

To further characterize the design, results from additional simulation scenarios are provided. We investigate power for the non-inferiority comparison assuming that the underlying DGF rate for hypothermia is 31%, slightly worse than the pump DGF rate of 30%. We investigate power for the superiority comparison assuming a slightly larger difference, a 10% improvement in DGF rate for the combination, from 30% to 20%. Table 5 describes these additional scenarios.

Table 5: Additional Simulation Scenarios

Intervention Arm	Delayed Graft Function (DGF) Scenarios			
	Additional Non-Inferiority Scenarios		*Additional Superiority Scenarios*	
	All Same*	Combo Best*	Combo Best**	Mixed**
Pump	30%	30%	30%	30%
Hypo	31%	31%	30%	37.5%
Combo	30%	22.5%	20.0%	20.0%

Results are provided in Table 6. For the primary comparison of interest, when hypothermia is slightly worse than pump, there is 75% power to declare non-inferiority. For the secondary objective of establishing superiority of the combination,

when there is a 10% difference between combination and pump, there is 96.1 - 100% power to detect an improvement for the combination arm relative to each of the others.

Table 6: Results from Additional Simulation Scenarios

	Scenario			
	Non-Inferiority Scenarios		*Superiority Scenarios*	
	All Same*	Combo Best*	Combo Best**	Mixed**
Number of Donors: Total	1400	1397.1	1393.7	1166.3
Average Number of Donors: Normothermia	468.1	467.9	465.8	400.7
Average Number of Donors: Hypothermia	931.9	929.2	927.8	765.6
Average Number Kidneys: Pump	936.2	935.8	931.6	801.4
Average Number Kidneys: Hypo	931.7	925.7	926.8	732.3
Average Number Kidneys: Combo	932.2	932.7	928.9	798.9
Prob. Of Stopping Early for Inferiority: Pump	0	0.014	0.024	0.631
Prob. Of Stopping Early for Inferiority: Hypo	0.002	0.029	0.026	0.66
Prob. Of Stopping Early for Inferiority: Combo	0	0	0	0
Non-inferiority: Hypo vs Pump	0.759	0.750	0.856	0.047
Superiority: Combo vs Hypo	0.024	0.898	0.981	1
Superiority: Combo vs Pump	0.013	0.749	0.961	0.992
Superiority: Hypo vs Pump	0.020	0.050	0.045	0.785
Superiority: Combo vs Both Hypo and Pump	0.002	0.710	0.945	0.969
At Least One Arm Superior to Another	0.047	0.932	0.993	1

Finally, we show the power for the non-inferiority comparison across an increasing range of DGF rates for hypothermia while the DGF rates for both pump and combination are 30%. Table 7 shows that power for the non-inferiority comparison decreases as hypothermia has higher DGF rates than pump alone.

Table 7: Power for Non-Inferiority of Hypo Relative to Pump

DGF Rate for Hypo	Non-Inferiority of Hypo relative to Pump
0.30	0.863
0.31	0.759
0.32	0.632
0.33	0.490
0.34	0.348
0.35	0.228
0.36	0.139
0.37	0.075

Other General Statistical Considerations for the *Randomized Trial of Mild Hypothermia and Machine Perfusion in Deceased Organ Donors for Protection against Delayed Graft Function in Kidney Transplant Recipients*

July 20, 2017

Primary Endpoint Justification

DGF is defined as the need for dialysis in the kidney recipient during the first week after transplantation. The main reason to prevent DGF is to avoid dialysis. Dialysis is a choice of last resort and puts the graft at risk because of potential hypotension, risk of thrombosis, increase in hospitalization, and worse clinical outcome. There are also resultant increases in healthcare costs and consumption of resources. Being that DGF occurs in up to 50% of kidneys transplanted from brain dead organ donors, there is a genuine ability to demonstrate a treatment effect with a donor-based intervention.

While this study will not have the power to examine differences in long-term renal graft survival as a primary outcome measure, the one-year graft survival rates of all organs transplanted from enrolled donors will be evaluated as secondary outcome measures. These will also serve as safety measures to ensure that the benefits of preventing DGF are not outweighed by differences in long-term graft loss of any transplanted organ. In addition, the transplantation rates of each organ as well as the overall number of organs transplanted per donor will be compared between groups.

Missing Data

The primary analyses will be based on only kidneys with complete DGF data. Missing covariate values will be imputed as the mean value for the corresponding treatment arm. Sensitivity analyses to the primary efficacy analysis will be conducted for missing DGF outcomes. Sensitivity analyses will consider the extreme cases where all missing outcomes in one arm had DGF and all missing outcomes in another arm did not have DGF and all possible combinations in between.

Secondary Analyses of the Primary Endpoint

The primary endpoint will also be presented as a raw percentage unadjusted for the correlation between kidneys. The primary analysis model will be repeated without adjustment for other covariates. Additional model selection may be performed where non-significant covariates are removed from the model and other covariates that are found to be statistically significant are added.

Secondary Endpoints

Secondary endpoints include:

1. Graft survival of the kidney
2. Graft survival of other organs transplanted from enrolled donors (liver, lung, heart, and pancreas will be considered separately)
3. Survival of recipients who receive a kidney from an enrolled donor

4. Survival of recipients who receive other organs from an enrolled donor (liver, lung, heart, and pancreas will be considered separately)
5. Transplantation rates of each organ (kidney, liver, lung, heart, and pancreas will be considered separately)
6. Number of organs transplanted per donor

Secondary endpoints will be reported first by treatment group, unadjusted for other covariates as well as adjusted for the same set of covariates included in the primary analysis model. Model selection may be performed where non-significant covariates are removed from the model and other covariates that are found to be statistically significant are added. Creatinine will only be included for analyses related to the kidney.

Survival endpoints will be measured from the date of organ transplantation to the date of graft failure. The proportion surviving at one year from each treatment group along with the corresponding two-sided 95% confidence intervals will be presented.

The transplantation rates of all organs will be calculated as the number of organs transplanted among all organs available from all enrolled donors of the same type and will be presented by treatment group along with the corresponding two-sided 95% confidence interval. The number of organs transplanted per donor will be summarized by treatment group with descriptive statistics such as mean, median, and range.

Enrollment Feasibility

Based on assumptions related to each OPOs average number of brain dead organ donors per year with research authorization, the trial inclusion criteria, and the experience with the first Mild Hypothermia RCT, it is anticipated that 50-70% of each OPOs annual number of organ donors will be enrolled.

The annual average number of organ donors for each of the confirmed and anticipated sites, in likely order of participation is:

Dallas (TXSB) - 349
Houston (TXGC) - 328
Colorado (CORS) - 127
New England Donor Services (NEOB and CTOP) – 336
Arizona (DNAZ)– 183
Donor Network West (CADN, Northern Coastal California) – 299
North Carolina (NCNC) - 173
TOTAL – 1795 donors/year

Being that we intend to onboard each OPO on a rolling basis, after experience and comfort are gained at the prior site(s), reaching a total target enrollment of 2800 donors over three years is feasible.

OPO Education

Training modules are developed for each participating OPO outlining the study protocol and reporting requirements. The modules are aimed at front line staff, who are implementing hypothermia protocol in organ donors and placing kidneys on pulsatile perfusion devices. Every OPO staff member that is involved with the study at any level is required to review the module. Training is deployed from a central learning platform that will be accessible by each individual from the participating OPOs. Training is tracked electronically. Each OPO is responsible for ensuring the module link is provided for any new hires or to any employee who requires a remedial review.

Compliance with completion of the modules will be monitored by the Trial Manager and reported during weekly Trial Coordination team conference calls.

A Randomized Trial of Mild Hypothermia Machine Perfusion in Deceased Organ Donors for Protection Against Delayed Graft Function in Kidney Transplant Recipients

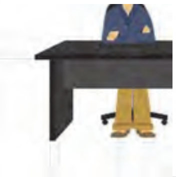

Training Module for Onsite Coordinators

Objective

- Overview of previous mild hypothermia trial
- Overview of current trial
  - Review of objectives of trial
  - Review of questions to be answered by the trial
  - Review of target enrollment and time frame
  - Review of inclusion criteria

What is the Study?

- Collaborative research effort w/UCSF, OHSU, UNOS, & your OPO
  - UCSF – IRB Institute of Record
- Funded by the Arnold Foundation
- Phase II
  - Hypothermia & machine perfusion better together?
- Prospective randomized trial across multi OPOs

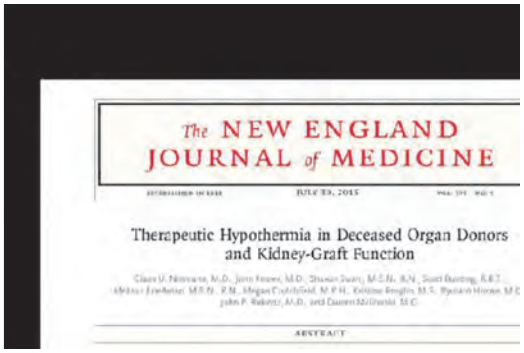
The NEW ENGLAND JOURNAL of MEDICINE

Therapeutic Hypothermia in Deceased Organ Donors and Kidney-Graft Function

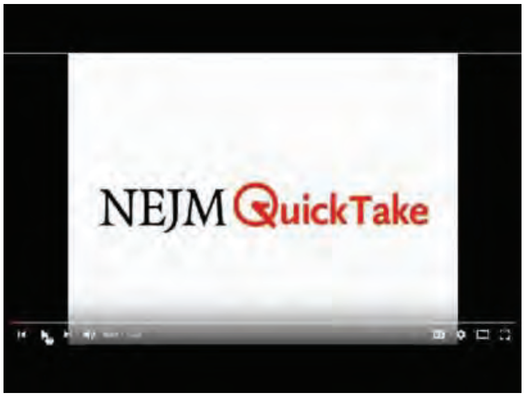

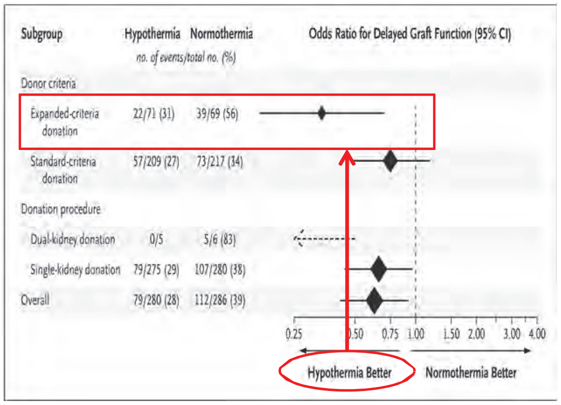

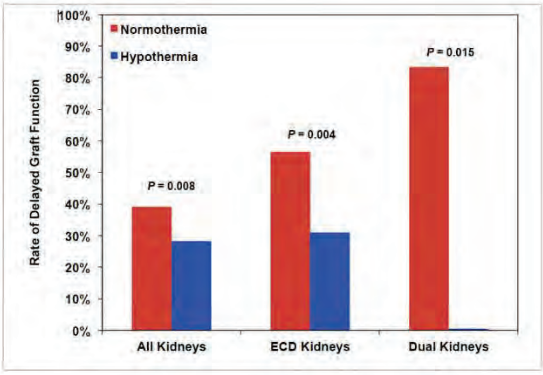

Main Objective of Current Trial

- Evaluate 2 strategies for improving function of kidneys for transplant:
  - Mild hypothermia applied to the donor before organ procurement
  - The use of machine perfusion of the kidney after procurement
    - Based on OPO pump criteria

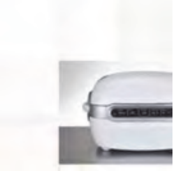

Questions to be Answered

- Is mild hypothermia alone **OR** machine perfusion alone better in protection of pump eligible kidneys?
- Is mild hypothermia **AND** machine perfusion better in protection of pump eligible kidneys?
- Is mild hypothermia superior to normothermia in the donors who are not pump eligible?
- Is mild hypothermia a safe intervention for extra renal organs?

Inclusion Criteria & Ta[illegible] Enrollment

- Brain dead donors
- 18 years or older
- No dialysis
  - ESRD
  - During current admission
- Research authorization
  - Via registry/FPA
  - From authorizing party

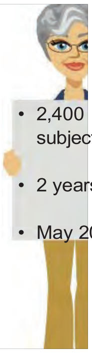

- 2,400 subjects
- 2 years
- May 2017

Objectives - Donor Managemer Coordinators

- Review enrollment criteria
- Discuss strategies for implementation & maintenance of mild hypothermia during do management
- Discuss management strategies in the recove room
- How to document data on the study algorith sheet

Enrollment Eligibility

- Determine suitability at ~12 hours after start of management
  - Vasopressor requirements = hemodynamic stability
    - Neosynephrine ≤ 60mcg/min
    - Levophed ≤ 10mcg/min
    - Dopamine ≤ 10mcg/kg/min
    - T4, solumedrol, vasopressin – any dose OK
    - Epinephrine at any dose excludes enrollment
  - Electrolytes
    - Potassium >3.5
    - Magnesium ≥2.0
  - Coagulation
    - INR < 2.5
    - PTT < 3x normal value
    - Platelet count > 50,000

Group Assignments

- Randomized by centralized computer software
  - Normothermia -- 36.5 – 37.5C (97.7 – 99.5F)
  - Hypothermia -- 34 - 35C (93.2 – 95F)
  - Pump determination will not impact donor management

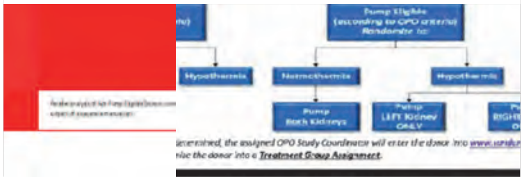

Mild Hypothermia (34-35C)

- Record starting temp (core)– rectal/foley/esophageal/PA
- Cooling/warming device
  - Water blanket (under donor)
    - Set at 30C until temp within 1-2° of target then set at target temp
      - Place bath blanket over top
  - Arctic Sun
    - Set at target temp
  - Bair Hugger
    - set at lowest setting until target temperature reached then to ambient setting

Mild Hypothermia 34-35°

- Ice packs
- Ambient

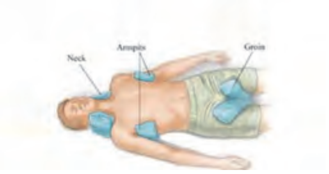

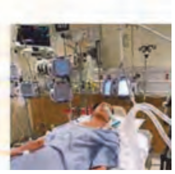

Mild Hypothermia (34-35C)

- Goal: 4 hours to reach target temp
  - ≥ 12 hours maintain at target temp
- Check tem[redacted]

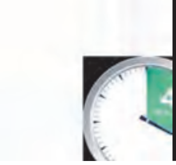
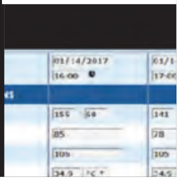

Transporting w/Mild Hypothermia

- Warm blanket over head
- Warm blanket over body
- Warming devices for prolonged procedures
- Increase room temperature
- Reduce exposure
- If Arctic Sun – pads insulate up to 45 minutes
- Goal – not allow temp to drop below 34C
  - Monitor temp if at all possible

Going to the OR

- Warm blanket over head & body during transport
- Warming blanket under body
  - Between sheet and table
  - Set to 36C
- Bair Hugger over legs
  - Medium to high setting
- Turn the devices off at the time heparin is g ven or w th n 5 m nutes of cross-c amp

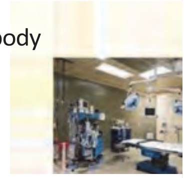

Electrolyte Management

- Monitor electrolytes closely & replace per protocol

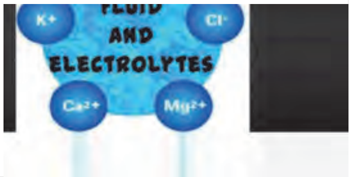

Normothermia (36.5-37.5C)

- Record starting temp (core)
- Check temp Q1H & record
- Should not require any intervention beyond normal donor management

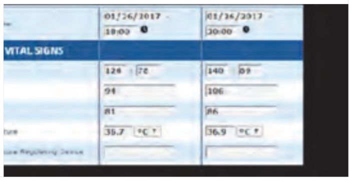

Potential Adverse Effects of Mil Hypothermia

- The degree of hypothermia is mild
- Adverse effects in this temperature range are not reported
  - In the literature adverse effects have been occasionally reported with moderate hypothermia (31-33 C)
- If the following occurs contact the Study Coordinator:
  - Hemodynamic instability
  - Increased urine output (not related to DI or glycosuria)
  - Coagulopathy (INR >2.5, PTT>3x nml, plt<50K)
  - Electrolyte depletion (K, Mg, Phos)
  - Arrhythmias

Recording & Monitoring Temperature

- Data collection on algorithm sheet by onsite coordinators/Study Coordinators
- Ensure core temp
  - Foley/rectal/esophageal/PA line
- Record temp Q1H in electronic donor record
- Ensure access to cooling/warming device/method

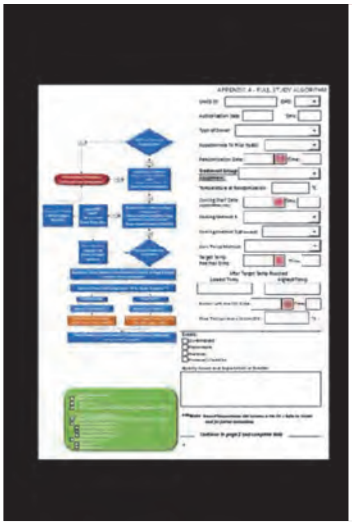

Please attach the completed document to the electronic donor record

Enter requested information here

This section is primarily for the study coordinator to complete

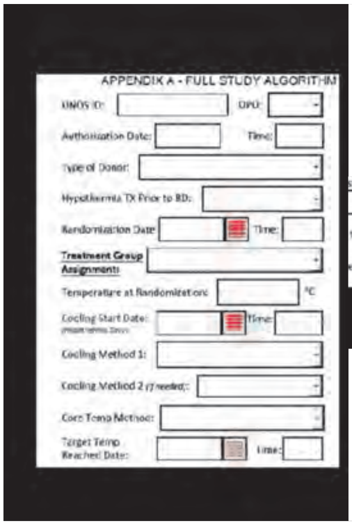

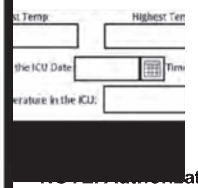

[illegible]ation date/time may be changed to Start of Donor Management date/time

Monitoring the Trial

- Reportable event
  - Clinical event that is self-limit
- Adverse event
  - Clinical event that requires in
- Protocol violation
  - Parameters that fall outside of range
- Dis-enrollment
  - Removed from the study altogether for any reason

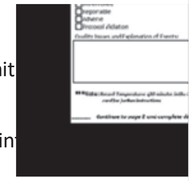

Communication

- How Transplant Centers Notified
  - General message to UNOS in eNewsletter
  - A study summary & UCSF IRB review will be attached to DonorNet for each subject enrolled
  - Scripted message regarding enrollment will be placed in donor highlights

NOS Transplant Pro

Objectives – Pump Criteria

- Overview of determination for pump eligibility
- Review randomization strategy
- Discuss data collection

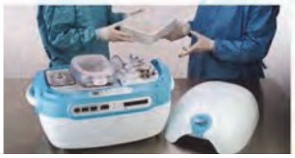

Pumping of Kidneys

- Randomization to pumping will be determined up front when eligibility met based on pre-defined OPO pump criteria

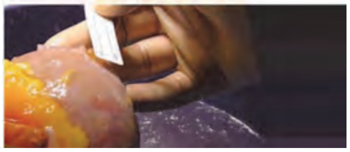

Current Pump Criteria

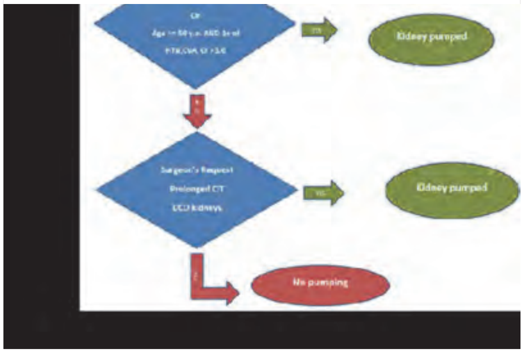

A Closer Look at Pump Randomization

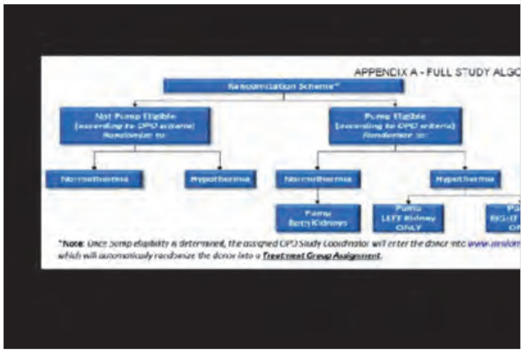

Recording the Data

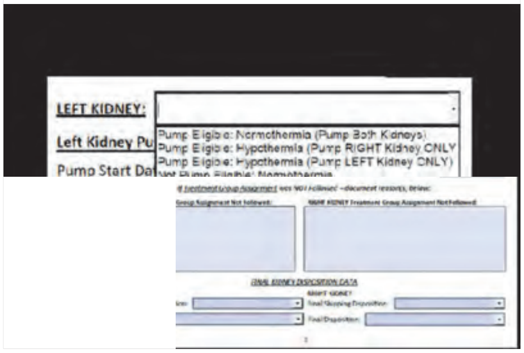

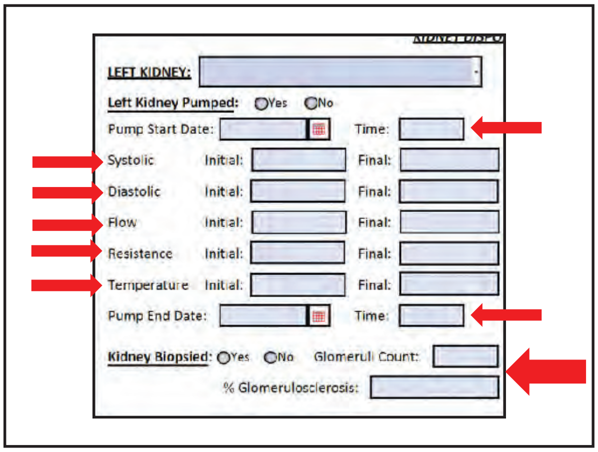

Deviation of Assignment

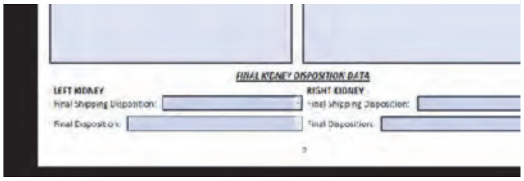

Final Disposition

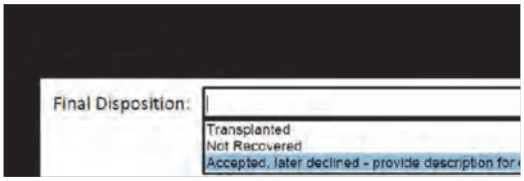

Who to Contact

- Study Coordinator for your OPO
- Local site PI

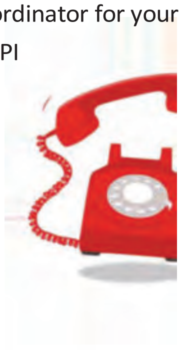

Principal Investigators

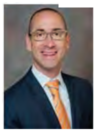

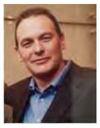

Study and Data Coordinating Center

- **UNOS Business Services**
  700 N. 4th St, Richmond, Virginia. 23219

- **Trial Manager**: Alex Garza
- **Data Analytics Manager**: Sarah Taranto
- **Data Quality**: Catherine Monstello, Brooke Chenault, Robert Carrico, PhD
- **Administrative Oversight**: Ryan Ehrensberger

Independent Data Safety and Monitoring Board

Roger J. Lewis, MD, PhD, Department of Emergency Medicine Harbor-UCLA Medical Center, Bldg D9, 1000 W Carson St, Torrance, CA 90509 Torrance, CA, USA

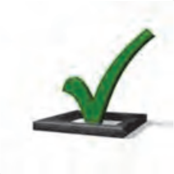

Thank You!

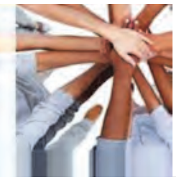

Communication

Participating Donor Service Areas:

Southwest Transplant Alliance – Greater Dallas area

LifeGift – Houston and Fort Worth areas

Other sites TBD

Transplant Community at Large:

The UNOS Region 5 Research Committee has already approved the protocol. Notification will be sent via the United Network for Organ Sharing (UNOS) eNewsletter to inform the transplant community at large about the upcoming study and to allow for a two-week public comment period, during which time questions can be asked. Study protocols will be made available on http://www.transplantpro.org and shared as requested.

Organ Offer to Transplant Centers:

With each subject enrollment, information about organs offered for transplantation from enrolled DNDDs will be posted on DonorNet®, (Richmond, Virginia, USA). In addition to standard data, it w il l contain information about the study protocol and potential treatment group assignments based on pump eligibility status. This allows any transplant center and surgeon to contact the PI or Co-PI at any time if there are questions concerning donor enrollment and the implications for organs offered for transplantation. The allocation of organs to specific recipients will occur based on standard OPTN guidelines and local transplant center acceptance criteria.

Data Collection

Document Templates

Documents listed below are provided in separate files.

21. Trial Summary
22. Study Algorithm
23. Enrollment Instructions
24. Event Form

Data Collection / Coordination

Data that will be specifically collected for this study are illustrated in **Appendix A**. These data sheets will be sent to UNOS Business Services, the study and data coordination site for the trial. UNOS will combine these unique trial data with other existing data sources in a de-identified, secure manner and send complete data sets to the DSMB for the trial. Using this approach, the study team members never have access to any identifiable information related to living individuals.

Donor-Specific Data

Donor demographic, medical history, and critical care data contained within the UNOS Donor Management Goals Registry will be combined with the unique trial data. Creatinine levels are used for calculation of GFR (Modification of Diet in Renal Disease Study, http://www.nephron.com/MDRD_GFR.cgi). Creatinine and GFR are measured at study enrollment and just prior to organ recovery (terminal creatinine). The Kidney Donor Profile Index (KDPI), a cumulative percentage scale that represents an overall estimate of the risk of graft failure for an individual donor, is contained within the DMG Registry and will be compared between groups. The KDPI includes age, weight, height, ethnicity/race, history of hypertension or diabetes, cause of death, serum creatinine, HCV status, and whether or not the organ donor meets DCD criteria.

Recipient-Specific Data

UNOS will combine the unique trial data elements with transplant recipient outcome and risk factor data contained within their administrative UNet database. Transplant centers are required to submit standardized clinical data elements on all organ recipients for the purposes of regulatory oversight. These data elements are also available to the general public for research purposes. We will only be utilizing these existing data elements and there will be no interaction with recipients and no data collection from recipients for the purpose of this study.

Recipient variables known to impact graft function and that are included in established Scientific Registry of Transplant Recipient (SRTR) risk-adjusted models will be combined with the donor data elements listed above and sent to the DSMB. These variables include recipient age, sex, ethnicity, warm ischemia time, and cold ischemia time, among other various data points. The full models are available at: http://www.srtr.org/csr/current/Centers/201412/modtabs/Risk/KIADC2G.pdf.

Trial Coordination Team

The Trial Coordination Team will consist of the PIs, Darren Malinoski, MD and Claus Niemann, MD; one representative from each OPO/DSA, Alex Garza, Sharon Swain, RN, MSN; and Jenna Graciano, MBA. The committee will discuss weekly progress of the study including rates of organ donor enrollment, protocol compliance, reportable or adverse events, and integrity of data collection.

Data Safety Monitoring Board (DSMB):

The DSMB will consist of Roger Lewis, MD, PhD and Berry Consultants.

Trial Timeline

**Quarters starting July 2017**	1	2	3	4	5	6	7	8	9	10	11	12
Orientation/Training first 3 OPOs	x											
Implementation of Protocols first 3 OPOs	x	x										
Database Training first 3 OPOs	x											
Enrollment of DNDD first 3 OPOs		x	x	x	x	x	x	x	x	x	x	x
Orientation/Training additional 3-5 OPOs		x										
Implementation of Protocols additional 3-5 OPOs		x	x									
Database Training Additional 3-5 OPOs		x										
Enrollment of DNDD Additional 3-5 OPOs			x	x	x	x	x	x	x	x	x	x
Collection DMG		x	x	x	x	x	x	x	x	x	x	x
Interim-Analysis*												
Final-Analysis												x
Manuscripts								x				x

* Exact time TBD and depending on enrollment progress. Each donor population will be monitored separately

RCT Fillable Enrollment Form

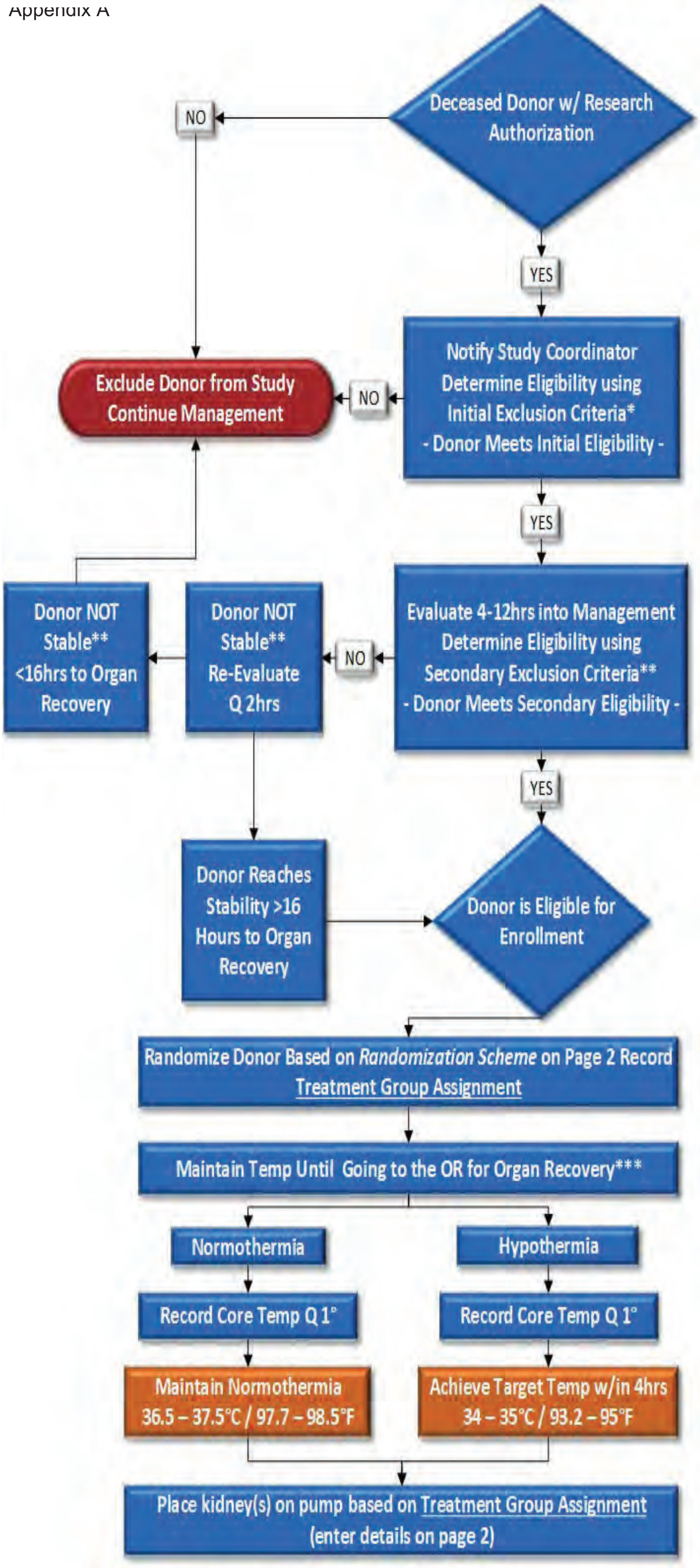

*Initial Exclusion Criteria

- ☐ 1. DCD Donor
- ☐ 2. Age <18 years
- ☐ 3. End Stage renal Disease (ESRD) or Dialysis during current admission

**Secondary Exclusion Criteria

- ☐ 1. Coagulopathy (INR>2.5, PTT >3x normal value, &/or PLT <50,000)
- ☐ 2. Hemodynamic Instability w/ either:
  - Dopamine >10mcg/kg/min
  - Norepinephrine >0.2mcg/kg/min
  - Neosynephrine >1mcg/kg/min
  - Epinephrine (any dose)

  If dose in EMR is mcg/min divide by dry weight to convert to mcg/kg/min

  WEIGHT ([illegible] ______
- 3. Electrolytes
  - ☐ Potassium < 3.5
  - ☐ Magnesium ≤ 2.0

UNOS ID: ______ OPO: ______

Authorization Date: ______ Time: ______

Type of Donor: ______ Pump Eligible: ______

Hypothermia TX Prior to BD: ______

Randomization Date: ______ Time: ______

Treatment Group Assignment: ______

Temperature at Randomization: ______ °C

Cooling Start Date: ______ Time: ______
(Hypothermia Only)

Cooling Method 1: ______

Cooling Method 2 *(if needed)*: ______

Core Temp Method: ______

Target Temp Reached Date: ______ Time: ______

After Target Temp Reached

Lowest Temp	Highest Temp

Donor Left the ICU Date: ______ Time: ______

Final Temperature in the ICU: ______ °C

Events:

- ☐ Dis-enrolled
- ☐ Reportable
- ☐ Adverse
- ☐ Protocol Violation

Quality Issues and Explanation of Events:

***Note: *Record Temperatures q30 minutes in the OR – Refer to Pocket card for further instructions*

Continue to page 2 and complete data

FULL STUDY ALGORITHM

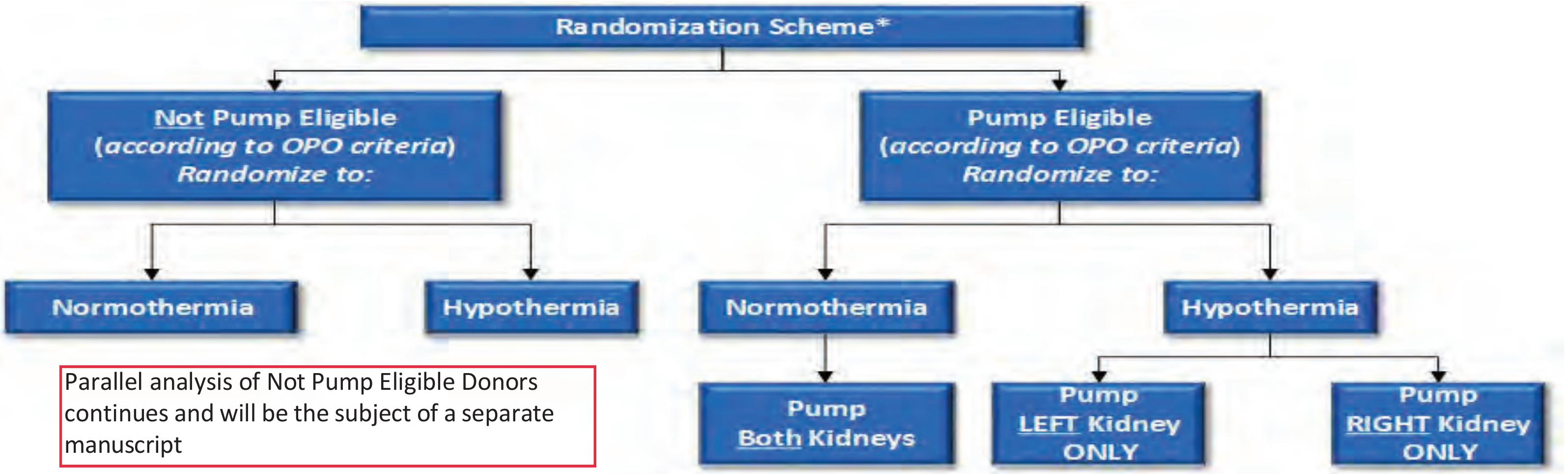

*__Note__: *Once pump eligibility is determined, the assigned OPO Study Coordinator will enter the donor into www.randomize.net which will automatically randomize the donor into a* ***Treatment Group Assignment***.

KIDNEY DISPOSITION DATA

LEFT KIDNEY:

Left Kidney Pumped: ○Yes No

Pump Start Date: ______ Time: ______

Systolic Initial: ______ Final: ______

Diastolic Initial: ______ Final: ______

Flow Initial: ______ Final: ______

Resistance Initial: ______ Final: ______

Temperature Initial: ______ Final: ______

Pump End Date: ______ Time: ______

Kidney Biopsied: ○Yes ○No Glomeruli Count: ______

% Glomerulosclerosis: ______

RIGHT KIDNEY:

Right Kidney Pumped: ○Yes ○No

Pump Start Date: ______ Time: ______

Systolic Initial: ______ Final: ______

Diastolic Initial: ______ Final: ______

Flow Initial: ______ Final: ______

Resistance Initial: ______ Final: ______

Temperature Initial: ______ Final: ______

Pump End Date: ______ Time: ______

Kidney Biopsied: ○Yes ○No Glomeruli Count: ______

% Glomerulosclerosis: ______

If Treatment Group Assignment was NOT Followed – document reason(s) below:

LEFT KIDNEY Treatment Group Assignment Not Followed:

______

RIGHT KIDNEY Treatment Group Assignment Not Followed:

______

FINAL KIDNEY DISPOSITION DATA

LEFT KIDNEY

Final Shipping Disposition: ______

Final Disposition: ______

Decline Reason: ______

RIGHT KIDNEY

Final Shipping Disposition: ______

Final Disposition: ______

Decline Reason: ______

Drop-down Options:

1. OPO
   - CADN
   - TXGC
   - TXSB
2. Type of Donor
   - ECD
   - SCD
3. Hypothermia TX Prior to BD
   - Yes
   - No
4. Treatment Group Assignment
   - Pump Eligible: Normothermia (Pump Both Kidneys)
   - Pump Eligible: Hypothermia (Pump RIGHT Kidney ONLY)
   - Pump Eligible: Hypothermia (Pump LEFT Kidney ONLY)
   - Not Pump Eligible: Normothermia
   - Not Pump Eligible: Hypothermia
5. Cooling Method 1
   - Cooling Blanket
   - Passive Cooling
   - Bair Hugger
   - Ice Packs
   - Artic Sun
   - Other
6. Cooling Method 2
   - Cooling Blanket
   - Passive Cooling
   - Bair Hugger
   - Ice Packs
   - Artic Sun
   - Other
7. Core Temp Method
   - Rectal
   - Bladder
   - Esophageal
   - PA Line
8. R/L Kidney Shipping Disposition
   - Shipped on Pump
   - Shipped in Box
9. R/L Kidney Final Disposition
   - Transplanted
   - Not Recovered
   - Accepted, later declined – provide description for decline

UCSF
University of California
San Francisco

Human Research Protection Program
Institutional Review Board (IRB)

Full Committee Approval

Principal Investigator
Dr. Claus Niemann, MD

Type of Submission: Submission Response for Initial Review Submission Packet
Study Title: A Randomized Trial of Mild Hypothermia and Machine Perfusion in Deceased Organ Donors for Protection against Delayed Graft Function in Kidney Transplant Recipients

IRB #: 17-21768
Reference #: 185808
Reviewing Committee: Parnassus Panel
Study Risk Assignment: Minimal

Approval Date: 04/17/2017 **Expiration Date:** 04/16/2018

Regulatory Determinations Pertaining to This Approval:

Note: This study involves deceased organ donors and their kidney recipients. The donors are not considered human subjects under Health and Human Services Code of Federal Regulations Title 45 Part 46. The following regulatory determinations were made regarding the recipients only.

This research is not subject to HIPAA rules.

A waiver of informed consent is acceptable because, as detailed in the application: (1) the research involves no more than minimal risk to the organ recipients; (2) the waiver will not adversely affect the rights and welfare of the organ recipients; (3) the research could not practicably be carried out without the waiver; and (4) whenever appropriate, the organ recipients will be provided with additional pertinent information after participation. The waiver of informed consent applies to all persons receiving a kidney managed by this protocol.

This research satisfies the following condition for the involvement of children: 45 CFR 46.404, 21 CFR 50.51: Research not involving greater than minimal risk.

The full board determined that the research poses no greater than minimal risk and is eligible for expedited review in the future under category #9 (continuing review of research, not conducted under an IND or IDE where categories 2 through 8 do not apply but the IRB has determined and documented at a convened meeting that the research involves no greater than minimal risk and no additional risks have been identified).

Comment:
Per the Office of Human Research Protections' definition of "engagement in research," the hospitals at which the recipients receive their transplants are *not* engaged in research because they are not performing any research procedures.

All changes to a study must receive UCSF IRB approval before they are implemented. Follow the modification request instructions. The only exception to the requirement for prior UCSF IRB review and approval is when the changes are necessary to eliminate apparent immediate hazards to the subject (45 CFR 46.103.b.4, 21 CFR 56.108.a). In such cases, report the actions taken by following these instructions.

Expiration Notice: The iRIS system will generate an email notification eight weeks prior to the expiration of this study's approval. However, it is your responsibility to ensure that an application for continuing review approval has been submitted by the required time. In addition, you are required to submit a study closeout report at the completion of the project.

Documents Reviewed with this Submission:

Study Document			
Title	Version #	Version Date	Outcome
Letter from UCSF Bioethicist Koenig	Version 1.0	02/20/2017	Approved
Letter from UPenn Bioethicist	Version 1.0	02/16/2017	Approved
Letter from Vanderbilt Bioethicist	Version 1.0	02/16/2017	Approved
Public Citizen complaint letter	Version 1.0	02/16/2017	Approved
UCSF Response re Public Citizen	Version 1.0	02/16/2017	Approved
Study Diagrams	Version 1.0	02/16/2017	Approved
Niemann NEJM 2015 Article on Previous Trial	Version 1.0	02/16/2017	Approved
Niemann Heldens Editorial on Donor Research	Version 1.0	02/16/2017	Approved
Watson_Editorial_NEJM_2015	Version 1.0	02/16/2017	Approved
Feng-2016-American_Journal_of_Transplantation	Version 1.0	02/16/2017	Approved
Abt_et_al-2016-American_Journal_of_Transplantation	Version 1.0	02/16/2017	Approved

For a list of all currently approved documents, follow these steps: Go to My Studies and open the study – Click on Informed Consent to obtain a list of approved consent documents and Other Study Documents for a list of other approved documents.

San Francisco Veterans Affairs Medical Center (SFVAMC): If the SFVAMC is engaged in this research, you must secure approval of the VA Research & Development Committee in addition to UCSF IRB approval and follow all applicable VA and other federal requirements. The IRB website has more information.

Acknowledgements

Institution of record for the trial:

University of California San Francisco

Local Institutional Advisory Panel:

Terri O'Lonergan, PhD, MA
Associate Vice Chancellor,
Chief Ethics and Compliance Officer
Institutional Official: Animal & Human Research
Research Integrity Officer
University of California San Francisco
415.502.3468
Theresa.OLonergan@ucsf.edu

Daniel Lowenstein, MD
Executive Vice Chancellor and Provost
University of California San Francisco
Lowenstein@ucsf.edu

Christopher M. Ryan, Ph.D.
Director, Human Research Protection Program
Director, CTSI Regulatory Knowledge and Support Program
University of California, San Francisco
Christopher.Ryan@ucsf.edu

Barbara Koening, PhD, RN
Director, Bioethics, University of California San Francisco
Medical Anthropology and Bioethics, University of California San Francisco
Barbara.Koenig@ucsf.edu

Katie Nolan, MPH, CIP
Regulatory Knowledge & Support (RKS)
Human Research Protection Program
University of California, San Francisco
Kate.Nolan@ucsf.edu

We also would like to acknowledge the continuous support and expertise of:

Melissa Greenwald, MD
Health Resources Services Administration (HRSA), Washington DC

David Klassen, MD
Chief Medical Officer for United Network for Organ Sharing (UNOS), Richmond, VA

Ryan Ehrensberger, Ph.D, FACHS
Director of Research, Director of UNOS Business Services, UNOS, Richmond, VA

A Randomized Trial of Mild Hypothermia and Machine Perfusion in Deceased Organ Donors for Protection against Delayed Graft Function in Kidney Transplant Recipients

Principal Investigators
Darren Malinoski, MD, Oregon Health & Science University, 3181 SW Sam Jackson Park Road, Portland, OR 97239, USA
malinosk@ohsu.edu

Claus Niemann, MD, University of California San Francisco, 500 Parnassus MUE 4th Floor Box 0648, San Francisco, Ca 94143-0648, USA
Claus.Niemann@ucsf.edu

Study and Data Coordinating Center
UNOS Solutions
700 N. 4th St, Richmond, Virginia. 23219

Trial Manager
Alex Garza

Transplant Analytics Service Line Leader
Sarah Taranto

Data Quality
Brooke Chenault

Administrative Oversight
Ryan Ehrensberger

Trials Design and Statistics

Kristine Broglio, M.S.
Berry Consultants, 4301 Westbank Dr #140B,
Austin,TX 78746 kristine@berryconsultants.net

Christina Saunders, PhD
Berry Consultants, 4301 Westbank Dr #140B,
Austin,TX 78746 christina@berryconsultants.net

Chair, Independent Data Safety and Monitoring Board

Roger J. Lewis, MD, PhD, Department of Emergency Medicine Harbor-UCLA Medical Center, Bldg D9, 1000 W Carson St, Torrance, CA 90509 Torrance, CA, USA

Education

Sharon Swain, RN, MSN, Clinical Nurse Educator, Donor Network West

Trial Coordination Team

Darren Malinoski, MD and Claus Niemann, MD, Alex Garza, BS, Sharon Swain, RN, MSN, Tahnee Groat, MPH, and one representative from each participating OPO

Organ Procurement Organization Sites

Southwest Transplant Alliance, 5489 Blair Rd, Dallas, TX 75231

Site PI: Jeffrey Reese, MD

Site Study Coordinator: Erin Vines

LifeGift, 2510 Westridge St, Houston, TX 77054

Site PI: R. Patrick Wood, MD

Site Study Coordinator: Monique Ferguson

Donor Alliance, 720 S Colorado Blvd #800n, Denver, CO 80246

Site PI: Katherine Fitting, MD

Site Study Coordinator: Lauren Abernathy and Grace Harcek, BSN, RN, CTBS

Donor Network of Arizona, 201 W Coolidge St, Phoenix, AZ 85013

Site PI: PJ Geraghty, MBA, CPTC

Site Study Coordinator: Kayla Gray, CCRP

Pacific Northwest Transplant Bank, 2611 SW 3rd Ave #320, Portland, OR 97201

Site PI: Craig Van De Walker, MS, CPTC

Site Study Coordinator: Rachel Nelson

LifeSource, 2225 West River Rd N, Minneapolis, MN 55411

Site PI: William Payne, MD

Site Study Coordinator: Kate Kishish, RN

Funding

This trial is supported by a grant from the Laura and John Arnold Foundation, Houston, Texas.

Abbreviations:

DGF – Delayed Graft Function
DMG – Donor Management Goals
DNDD – Donor after Neurologic Determination of Death
DSA – Donation Service Area
DSMB – Data Safety Monitoring Board
ECD – Expanded Criteria Donor
MP – Machine Perfusion
OPO – Organ Procurement Organization
RCT – Randomized Controlled Trial

Table of Contents

Section	Topic	Pages
I.	Cover Pages	46-47
II.	Abbreviations	48
III.	Table of Contents	49
IV.	Randomized Control Trial Summary	50-51
V.	Protocol	52-90
	a. Background	52-53
	b. Approach	54-56
	c. Targeted Temperature Management	57-58
	d. Target Population and Setting	59
	e. Adaptive Trial Design Report/Statistical Analysis	60-69
	f. Other General Statistical Considerations	70-71
	g. Education	72-79
	h. Communication	80
	i. Data Collection	81-82
	j. Trial Timeline	83
VI.	Appendix A. Full Study Algorithm- Data Collection Sheet	84-86
VII.	Appendix B	
	IRB Outcome Letter	87-90
	DSMB Recommendations	91-92
VIII.	Acknowledgments	93

Randomized Control Trial Summary - Page 1

- **"A Randomized Trial of Mild Hypothermia and Machine Perfusion in Deceased Organ Donors for Protection against Delayed Graft Function in Kidney Transplant Recipients"**
- **The Regents of University of California, San Francisco, and Oregon Health & Science University**

- **PI: Darren Malinoski, MD, FACS - malinosk@ohsu.edu, (503) 701-7628**
- **PI: Claus Niemann, MD - Claus.Niemann@ucsf.edu, (415) 502-2162**

BACKGROUND: In the initial Mild Hypothermia Randomized Control Trial (RCT), in collaboration with the UNOS Region 5 Donor Management Goals (DMG) Workgroup and Web Portal, the research team was able to conduct a multi-center RCT examining the benefits of mild hypothermia in donors after neurologic determination of death (DNDDs) on the outcomes of kidney transplantation. The trial was stopped early by the DSMB due to a significant positive benefit to kidney transplant recipients, including a 38% reduction in the odds of delayed graft function (DGF, the primary outcome measure of the trial). The results of this study have been published in the *New England Journal of Medicine* (July 2015). This research offers a zero-cost intervention that can substantially increase transplant success as well as the pool of potential donors.

To expand upon the success of the hypothermia study, the team is conducting a new RCT to test whether hypothermia is as effective as machine perfusion (MP) of kidneys from DNDDs. In an RCT conducted by the Eurotransplant International Foundation in 2009 (Moers et al. *NEJM*), the protective effect of MP (OR = 0.57) was similar to that found in our trial (OR = 0.62). However, the cost of MP can be very significant for organ procurement organizations (OPOs) and transplant centers.

MP of kidneys from deceased donors has been increasingly adopted by many centers even though clinical and cost effectiveness studies remain uncertain in the United States. Between 2012 and 2014, out of 31,798 kidneys available for transplant, 11,998 (38%) of them were machine perfused. Over the same three-year period, the number of kidneys pumped annually increased by over 20%.

This is an opportune time to investigate the effectiveness of MP compared to mild hypothermia, as there are enough OPOs currently using MP that if mild hypothermia was found to be a non-inferior intervention, there would be considerable cost savings. Similarly, over 60% of kidneys do not receive machine perfusion and findings that demonstrates a benefit of machine perfusion would likely lead to rapid increase in use. In addition, DGF still occurs in up to 56% of high-risk kidneys despite using one of these protective measures and their combined use may be the best approach moving forward. Either way, a new evidence-based standard will be created that will significantly affect the way kidney transplants are handled.

METHODS: This will be a pragmatic multi-site randomized controlled trial that bases enrollment on each OPO/Donation Service Area's current pumping criteria **(Figure 1**, page 2**).** There will be two main groups of DNDDs, (1) those that are "*pump eligible*" based on current practice (this group typically resembles traditional expanded criteria donors, but is increased in some areas) and (2) those that are lower risk and whose kidneys do not receive MP ("*not pump eligible*"). Kidneys from donors who are considered "pump eligible" currently receive MP based on their increased risk for failure. In this trial, "pump eligible" DNDDs will be randomized to one of three groups (**Figure 2**): (1) normothermia (36.5-37.5 C) plus MP of both kidneys (standard of practice control group), (2) mild hypothermia (34-35 C) plus MP of the left kidney only, and (3) mild hypothermia plus MP of the right kidney only. In this manner, the same number of kidneys will be randomized to each of the three treatment strategies (MP alone, mild hypothermia alone, or MP+hypothermia). **It is important to note that kidneys from "pump eligible"/higher risk DNDDs will still receive one form of protection and possibly two.**

In contrast, "not pump eligible" DNDDs will only be randomized to one of two groups: (1) therapeutic mild hypothermia or (2) normothermia. Being that our first trial was stopped early for efficacy in the overall DNDD population, there was insufficient statistical power to confirm a benefit in standard criteria donors (p=0.1 at stoppage). The purpose of this arm of the trial is to validate the protective effect of hypothermia in a larger sample size of lower-risk / "not pump eligible" donors.

The following objectives will be addressed by the trial:

- Determine the non-inferiority of a hypothermia-only strategy to a standard pump-only strategy in high risk DNDDs
- Evaluate the superiority of a combined hypothermia+MP strategy to both hypothermia or MP alone in high risk DNDDs
- Evaluate the superiority of mild hypothermia versus standard of care normothermia in lower risk, "not pump eligible" DNDDs
- Determine the safety of the hypothermia strategy with respect to the function of "bystander" organs (e.g., heart, lung)

This protocol has been approved by the UNOS Region 5 Research Committee. In addition, the following steps have been or will be taken:

- A National communication was sent via TransplantPro to allow for a two-week period for public comment.
- Only donors whose families and/or advanced directives (donor registry) have authorized research will be included in the study.
- All organ offers from DNDDs enrolled in the study will include a message in the "Donor Highlights" section of DonorNet, a copy of the study summary will be attached to the record, and allocation/transplantation will occur based on standard practice.
- There will not be any interaction between the study team and the transplant recipients and no additional data will be collected.
- Recipient graft function data will be derived from standard UNet forms and obtained from the OPTN in a de-identified format.

<u>**Randomized Control Trial Summary Page 2**</u>

- **"A Randomized Trial of Mild Hypothermia and Machine Perfusion in Deceased Organ Donors for Protection against Delayed Graft Function in Kidney Transplant Recipients"**
- **The Regents of University of California, San Francisco, and Oregon Health & Science University**
- **PI: Darren Malinoski, MD, FACS - malinosk@ohsu.edu, (503) 701-7628**
- **PI: Claus Niemann, MD - Claus.Niemann@ucsf.edu, (415) 250-1222**

Figure 1 – Participating OPO "Pump Eligible" Criteria

Sample OPO A

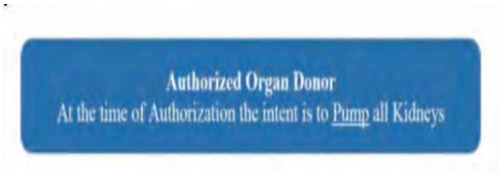

Sample OPO B

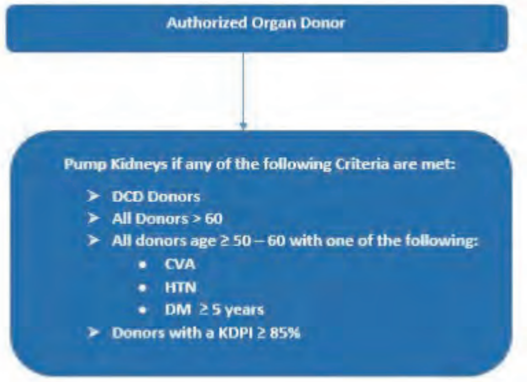

Figure 2 – Randomization Scheme

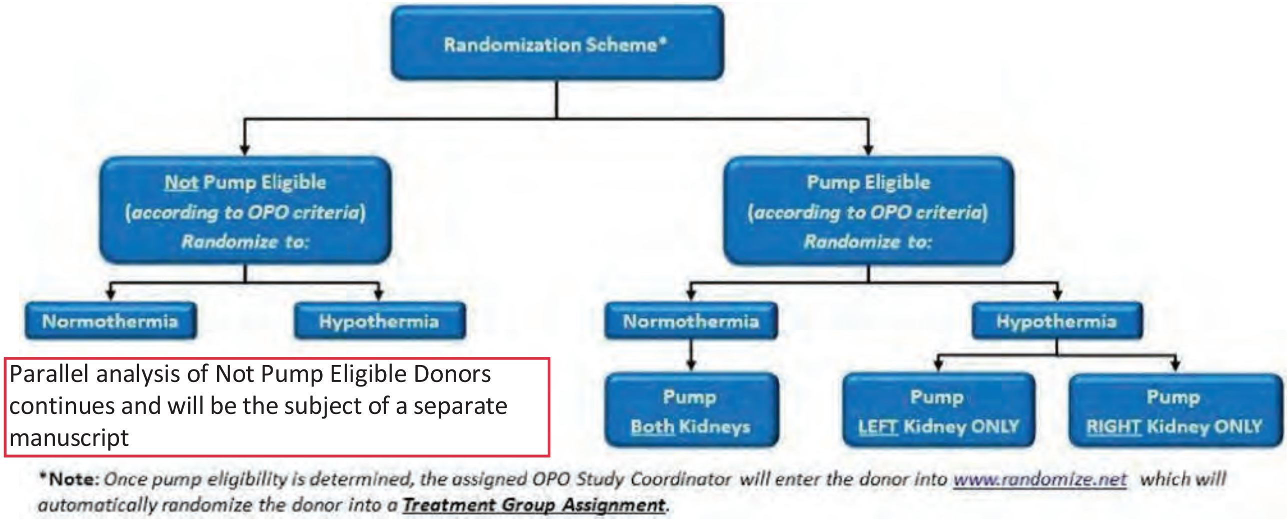

Background

Initial Hypothermia RCT

Utilizing the Donor Management Goal (DMG) Working Group and Web Portal, the research team was able to conduct a multi-center Randomized Control Trial (RCT) examining the benefits of therapeutic mild hypothermia (34° – 35° C) in organ donors after neurologic determination of death (DNDDs) on the outcomes of kidney transplantation. The trial was stopped early due to a significant positive benefit to kidney transplant recipients, including a 38% reduction in the odds of delayed graft function (DGF, the primary outcome measure). The results of this trial have been published in the *New England Journal of Medicine* (N Engl J Med. 2015 Jul 30;373(5):405-14) and are likely to significantly change the standard of care for deceased donor management around the world. In fact, the Executive Director of the Association of Organ Procurement Organizations has already hosted a national webinar to foster adoption of mild hypothermia as a part of standard deceased donor management protocols. This research offers a zero-cost intervention that can substantially increase transplant success and the pool of potential donors for decades to come.

This initial, successful study demonstrates the vast amount of unrealized potential that rigorously conducted donor intervention experiments can have on saving and enhancing lives through improved, evidence-based standards of donor management. In addition to being a scientifically successful study, it was an even bigger logistical accomplishment in that it covered a large geographic area, included all three phases of care through which an organ donor passes (donor hospital, organ procurement organization [OPO], and transplant center), and coordinated communications and oversight at the local, regional, and national levels. The unique collaboration and infrastructure developed to complete this study is poised to expand to additional regions and conduct the rigorous trials needed to advance the science in this field.

New Proposed Study: Hypothermia vs. Machine Perfusion of Kidneys

To expand upon the success of the hypothermia study, the team is prepared to conduct a new RCT to test whether hypothermia is as effective as machine perfusion of kidneys in preventing DGF. Machine perfusion is often used as an alternative to cold storage of kidneys during the period between organ removal and transplantation. With machine perfusion, the kidney is connected to a perfusion device, and a solution is pumped continuously through the vasculature. The cost of machine perfusion can be significant. The capital cost per machine can range from $25,000-$30,000 and each use can cost an additional $4,000-$6,000 in disposables (e.g. reagent solutions and single-use disposable cartridges) per kidney (not including labor costs).

Machine perfusion of kidneys from deceased donors has been rapidly adopted in the United States, despite the fact that clinical and cost effectiveness studies remain uncertain. Between 2012 and 2014, out of 31,798 kidneys available for transplant, 11,998 (38%) of them were machine perfused. Over the same three-year time period, the number of kidneys pumped annually increased by over 20%.

This is a key time to investigate the effectiveness of machine perfusion compared to mild hypothermia, as there are enough OPOs currently using machine perfusion that if mild hypothermia was found to be a non-inferior intervention, there would be considerable cost savings. Similarly, over 60% of kidneys do not receive machine perfusion and findings that demonstrate a benefit of machine perfusion would likely lead to rapid increase in use. Either way, a new evidence-based standard will be created that will significantly affect the way kidney transplants are handled.

Approach

Trial Overview – Randomization Scheme

This will be a pragmatic multi-site randomized controlled trial that bases enrollment on each OPO/Donation Service Area's (DSA) current pumping criteria (**Figure 1**). There will be two main groups of DNDDs, (1) those that are "*pump eligible*" based on current practice (this group typically resembles traditional expanded criteria donors [ECDs], but varies across each DSA) and (2) those that are lower risk and whose kidneys do not currently receive MP ("*not pump eligible*").

Figure 1 – Participating OPO "Pump Eligible" Criteria

Sample OPO A | **Sample OPO B** | **Sample OPO C**

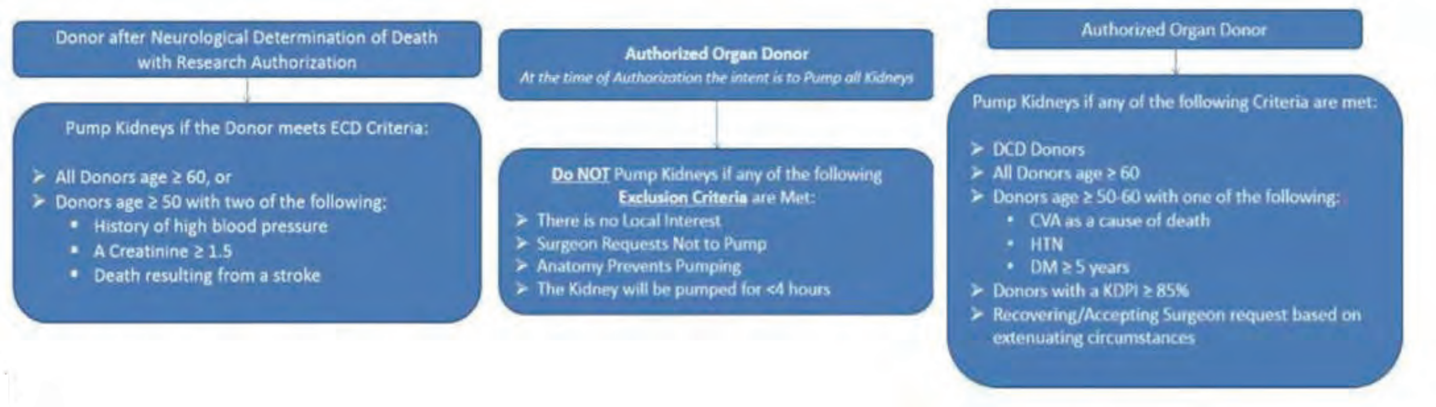

Kidneys from donors who are considered "pump eligible" currently receive MP based on their increased risk for failure. In this trial, "pump eligible" DNDDs will be randomized to one of three groups: (1) normothermia (36.5°-37.5° C) plus MP of both kidneys (standard of practice control group), (2) mild hypothermia (34°-35° C) plus MP of the left kidney only, and (3) mild hypothermia plus MP of the right kidney only (**Figure 2**). In this manner, the same number of kidneys will be randomized to each of the three treatment strategies (MP alone, mild hypothermia alone, or MP + hypothermia). It is important to note that kidneys from "pump eligible"/higher risk DNDDs will still receive one form of protection and possibly two.

In contrast, "not pump eligible" DNDDs will only be randomized to one of two groups: (1) therapeutic mild hypothermia or (2) normothermia. Being that our first trial was stopped early for efficacy in the overall DNDD population, there was insufficient statistical power to confirm a benefit in standard criteria donors (p=0.1 at the time of trial stoppage). The purpose of this arm of the trial is to validate the protective effect of hypothermia in a larger sample size of lower-risk / “not pump eligible” donors.

Figure 2 – Randomization Scheme

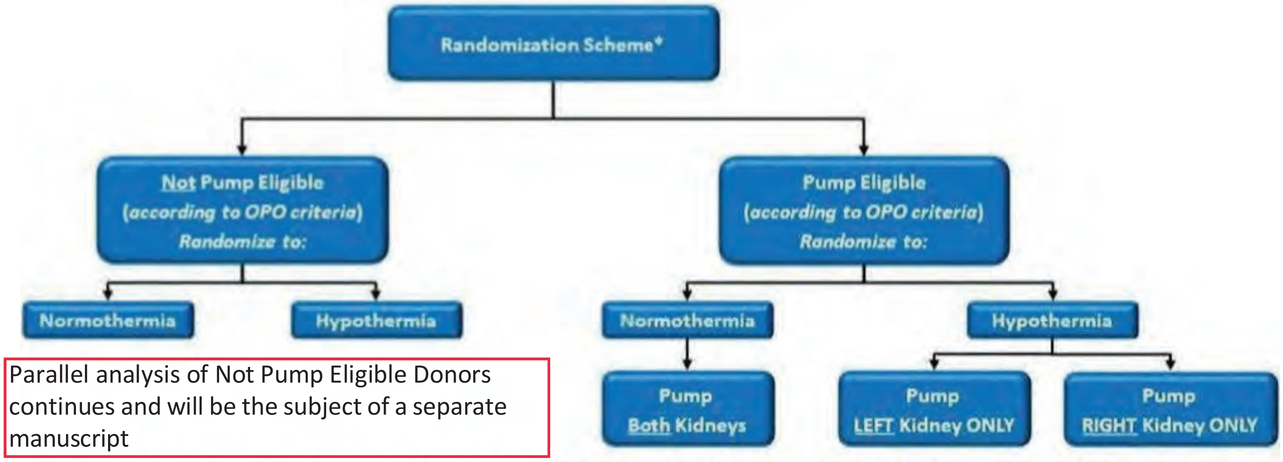

Note:** *Once pump eligibility is determined, the assigned OPO Study Coordinator will enter the donor into www.randomize.net which will automatically randomize the donor into a* ***Treatment Group Assignment.

Objective

This randomized clinical trial will evaluate two strategies for improving the post-transplant function of kidneys procured from deceased donors, mild hypothermia applied to the donor before organ procurement and the use of machine perfusion of the kidney after removal from the donor.

The following objectives will be addressed by the trial:

- Determine the non-inferiority of a hypothermia-only strategy to a standard, machine perfusion (MP)-only strategy in high risk, “pump-eligible” DNDDs
- Evaluate the superiority of a combined hypothermia+MP strategy to either hypothermia or MP alone in high risk DNDDs
- Evaluate the superiority of mild hypothermia versus standard of care normothermia in lower risk, "not pump eligible" DNDDs
- Determine the safety of the hypothermia strategy with respect to the function of “bystander” organs (e.g., heart, lung)

Targeted Temperature Management

Mild Hypothermia

Mild hypothermia will be achieved based on the successful protocol used in the previous trial. A detailed description of the protocol is provided below. Importantly, the described protocol is entirely non-invasive, associated with minimal to no cost, and safe when adhered to the protocol. The protocol will only be implemented after DNDDs are deemed stable after a 12-hour initial assessment period. The full study algorithm is depicted in **Appendix A**.

Criteria for initiation of mild hypothermia:

- Organ donors after neurologic determination of death (DNDDs) with research authorization
- There will be no gender or ethnic restrictions
- 18 years of age or older
- Hemodynamic stability – low dose vasopressors and MAP >60 mmHg for more than one hour without an increase in vasopressor dosages. Low-dose is defined as ≤ 1 vasopressor and below the following dosages:
    - Dopamine ≤ 10mcg/kg/min
    - Norepinephrine ≤ 0.2 mcg/kg/min
    - Neosynephrine ≤ 1 mcg/kg/min
    - Epinephrine – any dose is considered out of range
    - Vasopressin – not considered in pressor range
- Corrected coagulopathy
    - INR ≤ 2.5
    - PTT ≤ 3 x normal value
    - Platelet ≥ 50,000
- Corrected electrolytes & maintained per donor management protocol
    - K+ > 3.5 mmol/L
    - Mg++ ≥2.0 mg/dL

Absolute exclusion criteria

- DCD donors
- Dual Kidney Allocation
- Under 18 years of age
- Donors with ESRD (end stage renal disease)
- Dialysis during the terminal hospitalization
- Chronic medical condition precluding general acceptance for transplantation (i.e. cancer, diabetic or hypertensive nephropathy with unacceptable kidney biopsy findings)

Target range 34°-35°C for 12 hours minimum prior to leaving the ICU for organ recovery

1. Attempt to reach target temperature within 4 hours of initiation
2. Non-invasive devices only (invasive methods were not evaluated and should not be utilized) - If an invasive device has already been inserted by the donor hospital before OPO involvement, do not remove. It is OK to use such an existing device in this situation.
3. Cooling (water) blanket
4. Place under the donor for best effect. When target temperature reached place a blanket or sheet over the top while maintaining the cooling blanket
5. Precise temperature management system (non-invasive such as Arctic Sun or other similar device)
6. Bair Hugger or other air device on ambient setting
7. Ice packs
8. Ambient

Monitor and document core temperature Q1H by following methods:

9. Bladder temperature probe
10. Rectal temperature probe
11. Pulmonary artery catheter sensor
12. Esophageal temperature probe

Management protocols if procedures or organ recovery requiring travel are initiated (CT scan, angiography, recovery, etc.)

13. If already at target temperature place warm blankets over body and head for transport and attempt to keep covered during procedure
14. Monitor temperature if possible
15. If angiography or other prolonged procedures consider a warming device under the donor, place Bair Hugger (or other air warming device) over lower extremities and warm blanket over head in attempt to maintain target temperature

Specific intra-operative management goals in the hypothermia group
(Goal is to keep temp above 34°C prior to cross-clamp to prevent complications such as arrhythmias)

16. Monitor temp in OR
17. Warming blanket under donor as exposure can lead to more heat loss than desired(turned up to ~37ºC)
18. Bair Hugger on lower extremities (turned up to highest setting)
19. Warm blanket covering head
20. Increase room temperature

Target Population and Setting

Target Population

All DNDDs who have research authorization and meet the inclusion criteria are eligible. Authorization for research will be obtained by a procurement coordinator from each OPO if it is not already present within an advanced directive / state donor registry.

Setting

The proposal and the intervention will be implemented in all organ donor hospitals that are covered by participating OPOs. After declaration of death according to neurologic criteria and obtaining authorization for organ donation, care is transferred to the procurement coordinator. After a 12-hour assessment period, the intervention will be activated based on the inclusion criteria and randomization scheme. Point persons from each OPO are responsible for identifying eligible DNDDs, compliance with the authorization process, as well as implementation and execution of the protocol. In addition, the OPO point person will be responsible for being available for protocol questions that may arise from other OPO Coordinators within their own organization. This approach was highly successful in the previous trial.

Adaptive Design Report for the *Randomized Trial of Mild Hypothermia and Machine Perfusion in Deceased Organ Donors for Protection against Delayed Graft Function in Kidney Transplant Recipients*

Submitted in support of the Donor Management Research Initiative
July 20, 2017

1.0 Introduction

This document describes the statistical clinical trial designs for the evaluation of interventions for the prevention of delayed graft function (DGF) in kidney transplant recipients. Two deceased organ donor populations will be studied – those who are eligible for machine perfusion ("pump") of their kidneys and those who are not.

Each population will be considered separately. In the pump-eligible population, we will evaluate both hypothermia and pump. Only hypothermia will be studied in those donors not pump eligible. There will be interim analyses within each population to stop arms early if they are shown to be statistically inferior to the other arms in the population. This document is intended to provide details on the design, primary endpoint, primary analyses, interim analyses, and to present the operating characteristics of the trial for each organ donor population.

2.0 Populations

Donors will be enrolled into the trial and identified as being in one of two populations

Population A: Not Pump Eligible

Population B: Pump Eligible

3.0 Interventions and Randomization

Randomization will be performed separately within each of the two populations.

3.1 Population A: Not Pump Eligible

Donors will be randomized 1:1 to receive either normothermia or hypothermia.

3.2 Population B: Pump Eligible:

Kidneys will be randomized such that kidneys are allocated 1:1:1 to pump alone, hypothermia alone, or the combination of pump and hypothermia. Because hypothermia is performed at the donor level and pump is performed at the kidney level,

donors will be randomized to pump alone, to hypothermia with only the right kidney to be pumped, or to hypothermia with only the left kidney to be pumped.

4.0 Primary Objective

4.1 Population A: Not Pump Eligible

The primary objective is to determine if hypothermia is superior to normothermia in preventing DGF in kidney recipients.

4.2 Population B: Pump Eligible

The primary objective is to determine if hypothermia alone is non-inferior to pump alone in the prevention of DGF in kidney recipients. A key secondary objective is to determine if the combination of pump and hypothermia is superior to the individual therapies in the prevention of DGF in kidney recipients. This trial will ultimately elucidate the comparative-effectiveness of the three intervention arms.

5.0 Primary Endpoint

The primary endpoint in both populations will be delayed graft function (DGF).

6.0 Primary Analysis

The primary analysis method will be a GEE model in order to take into account the correlated nature of kidneys within a donor. The primary efficacy analysis will be based on the GEE model-estimated treatment effects and confidence intervals. The model will be fit for each population separately.

6.1 Population A: Not Pump Eligible

The primary analysis will be based on a GEE model fit including all kidneys with complete DGF information and a term for treatment groups categorized hypothermia versus normothermia. The full primary analysis model will include additional terms to adjust for covariates known to be associated with DGF. This includes terms for organ procurement agency, standard criteria donor vs expanded criteria donor, creatinine at enrollment, donor age, and kidney cold ischemic time. The GEE will use a compound symmetric correlation structure. Treatment effects will be reported in terms of the model estimated odds ratio comparing the two randomized treatment groups, and corresponding 95% confidence interval and two-sided p-value. A statement of superiority will be made based on a comparison of the observed p-value to the trial's nominal p-value required for success.

6.2 Population B: Pump Eligible

The primary analyses will be based on a GEE model fit including all concurrently enrolled kidneys with complete DGF information and a term for treatment group categorized as hypothermia versus normothermia versus the combination. The full

primary analysis model will include additional terms to adjust for covariates known to be associated with DGF. This includes terms for organ procurement agency, standard criteria donor vs expanded criteria donor, creatinine at enrollment, donor age, and kidney cold ischemic time. The GEE will use a compound symmetric correlation structure. Treatment effects will be reported in terms of the model estimated odds ratio comparing two randomized treatment groups, the corresponding 95% confidence interval and two-sided p-value.

We will report final model results for all pairwise comparisons

- Hypothermia versus Pump
- Hypothermia versus Combination
- Pump versus Combination

A statement of superiority will be made based on a comparison of the observed p-value to the trial's nominal p-value required for superiority. Holm's adjustment will be used to account for multiple statistical tests. If the pump and the hypothermia arms are not dropped, a statement of non-inferiority will be made based on a comparison of the final 95% confidence interval to the non-inferiority margin of 1.4.

7.0 Population for Analysis

The final analysis for each comparison of interest will only include data from concurrently randomized kidneys. This applies to only the pump eligible population. If an arm is dropped and additional kidneys are enrolled to the other arms, those additional kidneys will not be included in the analysis model for inference on the dropped arm.

The primary analysis for both populations will follow the intent-to-treat principle meaning that kidneys will be considered according to their randomized assignment regardless of the treatment received.

8.0 Interim Analyses

In each population, there will be 4 interim analyses to drop an arm for inferiority. If only one arm remains, the trial in that population will stop early.

8.1 Population A: Not Pump Eligible

There will be 4 interim analyses during the conduct of the trial to monitor for the inferiority of one treatment arm versus the other (i.e. two-sided monitoring). Interim analyses will be conducted according to an O'Brien-Fleming group sequential stopping boundary. If one of the arms is found to be statistically inferior to the other, enrollment in this population will stop.

A maximum of 1400 donors will be enrolled in this population. Interim analyses are planned beginning when 600 donors have complete DGF outcome information and each

subsequent look is planned after every additional 200 donors have complete DGF outcome information.

8.2 Population B: Pump Eligible

There will be 4 interim analyses during the conduct of the trial to monitor for the inferiority of one or two arms. Interim analyses will be conducted according to an O'Brien-Fleming group sequential stopping boundary. An arm may be dropped if it is found to be inferior to the other arms open and enrolling in the population. Enrollment would continue in the remaining arm(s). Therefore, if all arms are open and being enrolled to, an arm must be statistically inferior to both other arms to be dropped. If an arm has already been dropped, then if an arm is inferior to the only other arm remaining in the trial, it will be dropped. If two arms are dropped, enrollment in this population would end.

A maximum of 1400 donors will be enrolled in this population. Interim analyses are planned beginning when 600 donors have complete DGF outcome information and each subsequent look is planned after every additional 200 donors have complete DGF outcome information.

8.3 *Stopping Boundary*

Both populations have planned interim analyses occurring at the same information fraction. Therefore, both populations have the same O'Brien-Fleming boundary. The nominal two-sided p-values required to drop an arm for inferiority at each interim analysis are provided in the table below.

Table 1: O'Brien Fleming Stopping Boundary

Look<br>(# of Donors Complete)	Nominal two-sided p-value
600	0.0028
800	0.0087
1000	0.0174
1200	0.0276
Final Analysis	0.0386

8.4 Adjustmentfor Multiple Comparisons

In the pump eligible population, to account for the three pairwise comparisons between the three arms, dropping an arm for inferiority at each interim will be determined according to the following sequence:

1. Determine the global significance of treatment based on an ANOVA p-value between a full and reduced GEE model where the only difference between the full and reduced model is the inclusion or exclusion of the terms for treatment group.

Berry Consultants
Statistical Innovation

2. Compare the ANOVA p-value to the O'Brien-Fleming nominal two-sided p-values required for stopping at each look (Table 1). If the boundary is crossed, then conduct the pairwise comparisons.

   a. The p-values for each of the three pairwise comparisons will be adjusted according to Holm's procedure to account for the multiple statistical tests

   b. Compare the Holm's adjusted p-values to the boundary.

   c. If one arm crosses the boundary for inferiority versus both of the other arms open and enrolling in the trial, that arm will be dropped

Once an arm is dropped, there is only one comparison left in the population and so the above described approach reduces to a direct comparison between the unadjusted p-value for the single comparison left in the trial to the boundary.

9.0 Operating Characteristics

9.1 Population A: Not Pump Eligible

For the purpose of characterizing the trial, we assume that the DGF rate in the normothermia arm will be 30% and that the correlation between kidneys is 0.15. We assume 30% of all kidneys will not be transplanted and, therefore, will not be included in the analyses. A maximum of 1400 donors provides 90% power to detect an absolute improvement of 7.5% in DGF rates for hypothermia. Similarly, there is 80% power to detect an absolute improvement of 6%. The O'Brien-Fleming boundary controls the overall two-sided Type I error rate at 5%. Assuming an improvement of 7.5% in DGF rates for hypothermia, there is an 83% probability that enrollment in this population would stop early and the mean number of donors is 960. The absolute smallest difference between normothermia and hypothermia expected to be found to be statistical significant is approximately 4%.

9.2 Population B: Pump Eligible

In order to characterize the power and Type I error of the trial design in this population, we simulated the trial under varying scenarios for the delayed graft function (DGF) rate across the three arms; pump alone ("Pump"), hypothermia alone ("Hypo") and hypothermia in combination with pump ("Combo"). These scenarios allow us to assess the power and type I error for both the primary objective of non-inferiority between hypo and pump as well as the secondary objective of superiority between combo and the other arms.

We assume that the underlying DGF rate is 30% and that the correlation between the two kidneys from the same donor is 0.15. We assume that on average 30% of all kidneys will not be transplanted and, therefore, will not be included in the analyses.

We assess power for the non-inferiority comparison assuming that hypothermia and pump have the same underlying DGF rate of 30%. We assess Type I error for the non-

inferiority comparison assuming that one arm is at the non-inferiority margin. Given an underlying DGF rate of 30% and a NI margin on the odds ratio scale of 1.4, this translates to a DGF rate of 37.5%.

We assess power for the superiority comparison assuming a 7.5% improvement in DGF rate for the combination, from 30% to 22.5%. We assess Type I error for the superiority comparison in scenarios assuming the combination arm has the same DGF rate as another arm.

Results are presented for each simulation scenario. Table 2 shows the assumed true underlying DGF rates for each arm in each of the four scenarios. Table 3 shows the average number of donors, the average number of kidneys randomized to each of the three arms of the trial, and the probability of stopping an arm early due to inferiority. Table 4 shows the probability of declaring non-inferiority of hypothermia relative to pump, the probability of declaring superiority for each of the three comparisons, the probability of declaring the combination superior to both other arms, and the probability of declaring at least one of the three comparisons significant.

Table 2: **Simulation Scenarios**

Intervention Arm	Delayed Graft Function (DGF) Scenarios			
	All Same	Combo Best	Hypo Worst	Mixed
Pump	30%	30%	30%	30%
Hypo	30%	30%	37.5%	37.5%
Combo	30%	22.5%	30%	22.5%

Table 3: **Average Sample Size and Probability of Stopping Early for Inferiority**

	Scenario			
	All Same	Combo Best	Hypo Worst	Mixed
Number of Donors: Total	1400	1398.3	1398.9	1191.7
Average Number of Donors: Normothermia	468	466.9	495.9	430.8
Average Number of Donors: Hypothermia	932	931.4	903.0	760.9
Average Number Kidneys: Pump	936	933.9	991.8	861.7
Average Number Kidneys: Hypo	932	930.1	817.4	662.0
Average Number Kidneys: Combo	932.1	932.7	988.6	859.7
Prob. Of Stopping Early for Inferiority: Pump	0.001	0.013	0.002	0.621
Prob. Of Stopping Early for Inferiority: Hypo	0.001	0.013	0.440	0.823
Prob. Of Stopping Early for Inferiority: Combo	0.001	0	0.003	0

Table 4: Power and Type I Error

	Scenario			
	All Same	Combo Best	Hypo Worst	Mixed
Non-inferiority: Hypo vs Pump	0.863	0.862	0.049	0.013
Superiority: Combo vs Hypo	0.015	0.802	0.748	1
Superiority: Combo vs Pump	0.012	0.738	0.029	0.893
Superiority: Hypo vs Pump	0.013	0.034	0.683	0.912
Superiority: Combo vs Both Hypo and Pump	0.001	0.658	0.013	0.792
At Least One Arm Superior to Another	0.032	0.877	0.832	1

The maximum sample size of 1400 donors provides 86% power for the primary comparison of interest, the non-inferiority of pump and hypothermia. For the secondary objective of establishing the superiority of the combination, this trial has 73.8% to 100% power to detect an improvement relative to each of the other arms. There is 65.8% to 79.2% power to detect an improvement for the combination arm versus both the other arms in the trial. For both the non-inferiority and the superiority hypothesis, the overall two-sided Type I error rates are controlled at the 5% level.

We also explore the observed absolute differences between arms that result in either non-inferiority or superiority. Figures 1-3 show the simulation results, plotting the observed data and trial outcome. The plots pool results across the above simulation scenarios. Across all simulated trials, the smallest difference between combination and either pump alone or hypothermia alone expected to be found to be statistical significant is approximately 4.2%. The largest difference between hypothermia alone and pump alone expected to result in statistical non-inferiority is approximately 3.6%

Figure 1: Observed Data Resulting in Non-inferiority

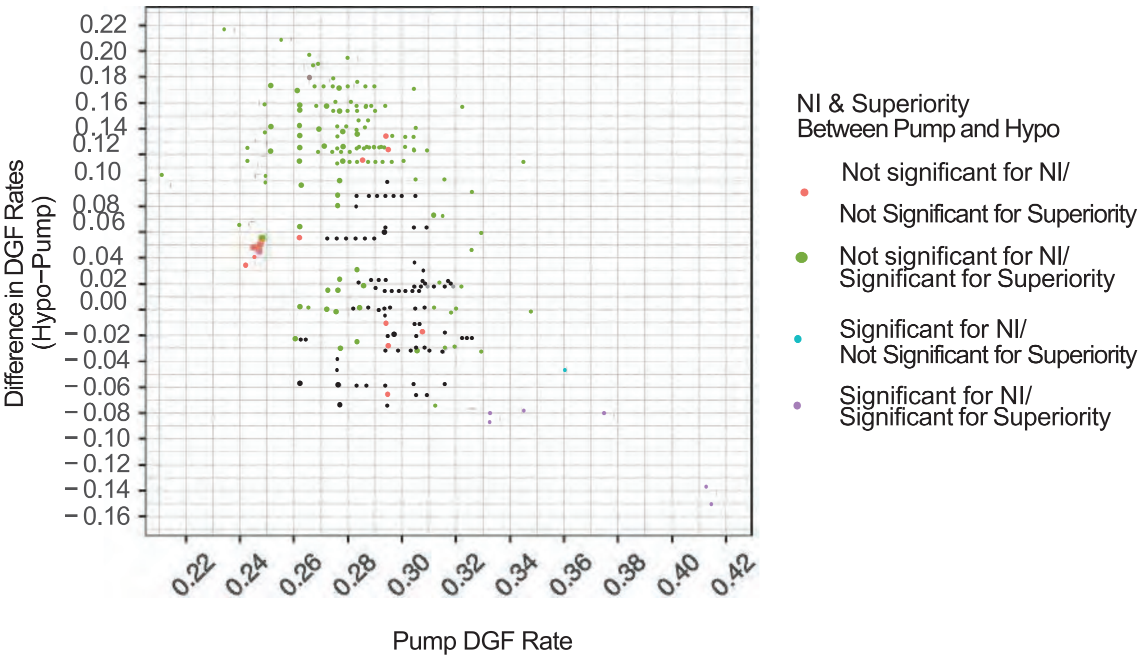

Figure 2: Observed Data Resulting in Superiority of Combo vs Hypo

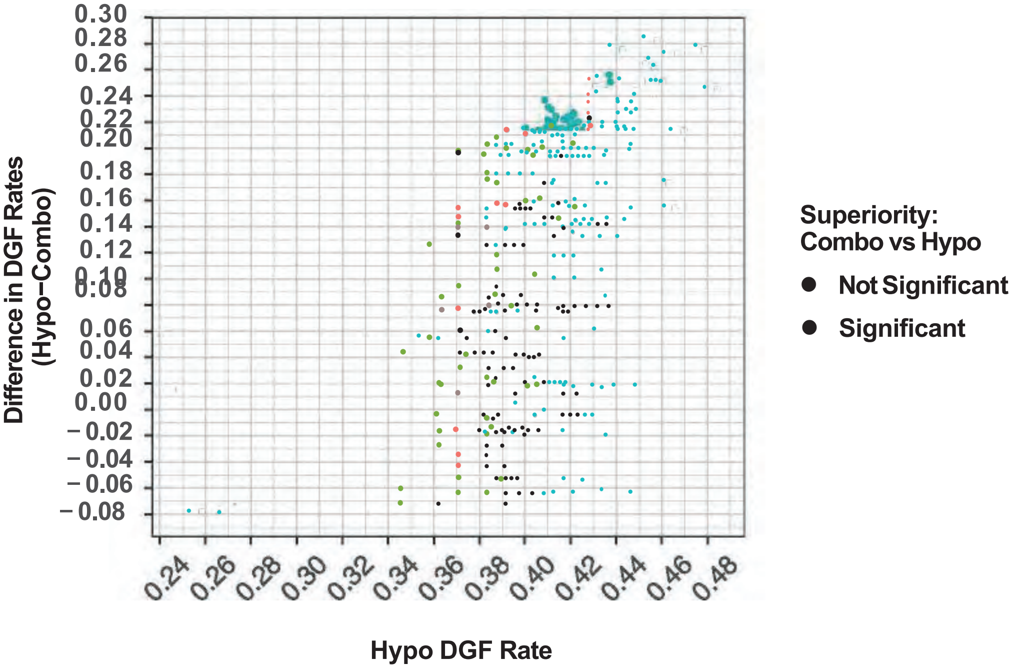

Figure 3: Observed Data Resulting in Superiority of Combo vs Pump

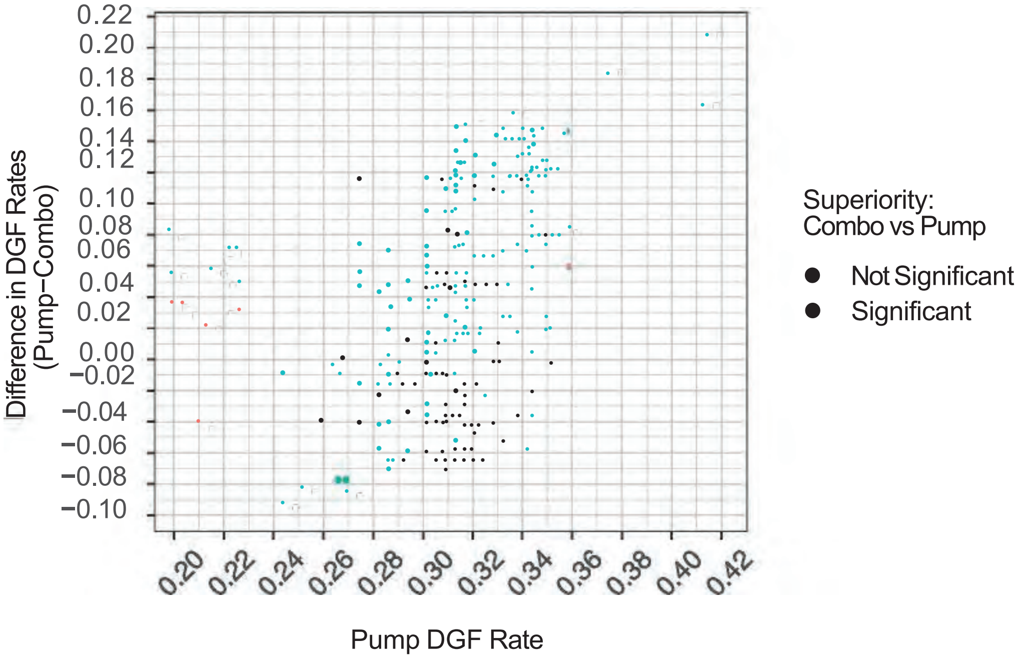

9.4 Additional Simulation Scenarios for Population B: Pump Eligible

To further characterize the design, results from additional simulation scenarios are provided. We investigate power for the non-inferiority comparison assuming that the underlying DGF rate for hypothermia is 31%, slightly worse than the pump DGF rate of 30%. We investigate power for the superiority comparison assuming a slightly larger difference, a 10% improvement in DGF rate for the combination, from 30% to 20%. Table 5 describes these additional scenarios.

Table 5: Additional Simulation Scenarios

Intervention Arm	Delayed Graft Function (DGF) Scenarios			
	Additional Non-Inferiority Scenarios		*Additional Superiority Scenarios*	
	All Same*	Combo Best*	Combo Best**	Mixed**
Pump	30%	30%	30%	30%
Hypo	31%	31%	30%	37.5%
Combo	30%	22.5%	20.0%	20.0%

Results are provided in Table 6. For the primary comparison of interest, when hypothermia is slightly worse than pump, there is 75% power to declare non-inferiority. For the secondary objective of establishing superiority of the combination,

when there is a 10% difference between combination and pump, there is 96.1 - 100% power to detect an improvement for the combination arm relative to each of the others.

Table 6: **Results from Additional Simulation Scenarios**

	Scenario			
	Non-Inferiority Scenarios		*Superiority Scenarios*	
	All Same*	Combo Best*	Combo Best**	Mixed**
Number of Donors: Total	1400	1397.1	1393.7	1166.3
Average Number of Donors: Normothermia	468.1	467.9	465.8	400.7
Average Number of Donors: Hypothermia	931.9	929.2	927.8	765.6
Average Number Kidneys: Pump	936.2	935.8	931.6	801.4
Average Number Kidneys: Hypo	931.7	925.7	926.8	732.3
Average Number Kidneys: Combo	932.2	932.7	928.9	798.9
Prob. Of Stopping Early for Inferiority: Pump	0	0.014	0.024	0.631
Prob. Of Stopping Early for Inferiority: Hypo	0.002	0.029	0.026	0.66
Prob. Of Stopping Early for Inferiority: Combo	0	0	0	0
Non-inferiority: Hypo vs Pump	0.759	0.750	0.856	0.047
Superiority: Combo vs Hypo	0.024	0.898	0.981	1
Superiority: Combo vs Pump	0.013	0.749	0.961	0.992
Superiority: Hypo vs Pump	0.020	0.050	0.045	0.785
Superiority: Combo vs Both Hypo and Pump	0.002	0.710	0.945	0.969
At Least One Arm Superior to Another	0.047	0.932	0.993	1

Finally, we show the power for the non-inferiority comparison across an increasing range of DGF rates for hypothermia while the DGF rates for both pump and combination are 30%. Table 7 shows that power for the non-inferiority comparison decreases as hypothermia has higher DGF rates than pump alone.

Table 7: Power for Non-Inferiority of Hypo Relative to Pump

DGF Rate for Hypo	Non-Inferiority of Hypo relative to Pump
0.30	0.863
0.31	0.759
0.32	0.632
0.33	0.490
0.34	0.348
0.35	0.228
0.36	0.139
0.37	0.075

Other General Statistical Considerations for the *Randomized Trial of Mild Hypothermia and Machine Perfusion in Deceased Organ Donors for Protection against Delayed Graft Function in Kidney Transplant Recipients*

Primary Endpoint Justification

DGF is defined as the need for dialysis in the kidney recipient during the first week after transplantation. The main reason to prevent DGF is to avoid dialysis. Dialysis is a choice of last resort and puts the graft at risk because of potential hypotension, risk of thrombosis, increase in hospitalization, and worse clinical outcome. There are also resultant increases in healthcare costs and consumption of resources. Being that DGF occurs in up to 50% of kidneys transplanted from brain dead organ donors, there is a genuine ability to demonstrate a treatment effect with a donor-based intervention.

While this study will not have the power to examine differences in long-term renal graft survival as a primary outcome measure, the one-year graft survival rates of all organs transplanted from enrolled donors will be evaluated as secondary outcome measures. These will also serve as safety measures to ensure that the benefits of preventing DGF are not outweighed by differences in long-term graft loss of any transplanted organ. In addition, the transplantation rates of each organ as well as the overall number of organs transplanted per donor will be compared between groups.

Missing Data

The primary analyses will be based on only kidneys with complete DGF data. Missing covariate values will be imputed as the mean value for the corresponding treatment arm. Sensitivity analyses to the primary efficacy analysis will be conducted for missing DGF outcomes. Sensitivity analyses will consider the extreme cases where all missing outcomes in one arm had DGF and all missing outcomes in another arm did not have DGF and all possible combinations in between.

Secondary Analyses of the Primary Endpoint

The primary endpoint will also be presented as a raw percentage unadjusted for the correlation between kidneys. The primary analysis model will be repeated without adjustment for other covariates. Additional model selection may be performed where non-significant covariates are removed from the model and other covariates that are found to be statistically significant are added.

Secondary Endpoints

Secondary endpoints include:
1. Graft survival of the kidney
2. Graft survival of other organs transplanted from enrolled donors (liver, lung, heart, and pancreas will be considered separately)
3. Survival of recipients who receive a kidney from an enrolled donor

4. Survival of recipients who receive other organs from an enrolled donor (liver, lung, heart, and pancreas will be considered separately)
5. Transplantation rates of each organ (kidney, liver, lung, heart, and pancreas will be considered separately)
6. Number of organs transplanted per donor

Secondary endpoints will be reported first by treatment group, unadjusted for other covariates as well as adjusted for the same set of covariates included in the primary analysis model. Model selection may be performed where non-significant covariates are removed from the model and other covariates that are found to be statistically significant are added. Creatinine will only be included for analyses related to the kidney.

Survival endpoints will be measured from the date of organ transplantation to the date of graft failure. The proportion surviving at one year from each treatment group along with the corresponding two-sided 95% confidence intervals will be presented.

The transplantation rates of all organs will be calculated as the number of organs transplanted among all organs available from all enrolled donors of the same type and will be presented by treatment group along with the corresponding two-sided 95% confidence interval. The number of organs transplanted per donor will be summarized by treatment group with descriptive statistics such as mean, median, and range.

Enrollment Feasibility

Based on assumptions related to each OPOs average number of brain dead organ donors per year with research authorization, the trial inclusion criteria, and the experience with the first Mild Hypothermia RCT, it is anticipated that 50-70% of each OPOs annual number of organ donors will be enrolled.

The annual average number of organ donors for each of the confirmed and anticipated sites, in likely order of participation is:

Dallas (TXSB) - 349
Houston (TXGC) - 328
Colorado (CORS) - 127
Arizona (DNAZ)– 183
Donor Network West (CADN, Northern Coastal California) – 299
North Carolina (NCNC) - 173
TOTAL – 1459 donors/year

Being that we intend to onboard each OPO on a rolling basis, after experience and comfort are gained at the prior site(s), reaching a total target enrollment of 2800 donors over three years is feasible.

OPO Education

Training modules are developed for each participating OPO outlining the study protocol and reporting requirements. The modules are aimed at front line staff, who are implementing hypothermia protocol in organ donors and placing kidneys on pulsatile perfusion devices. Every OPO staff member that is involved with the study at any level is required to review the module. Training is deployed from a central learning platform that will be accessible by each individual from the participating OPOs. Training is tracked electronically. Each OPO is responsible for ensuring the module link is provided for any new hires or to any employee who requires a remedial review.

Compliance with completion of the modules will be monitored by the Trial Manager and reported during weekly Trial Coordination team conference calls.

A Randomized Trial of Mild Hypothermia and Machine Perfusion in Deceased Organ Donors for Protection Against Delayed Graft Function in Kidney Transplant Recipients

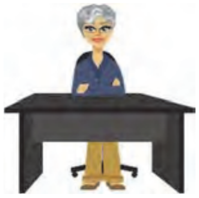

Training Module for Onsite Coordinators

Objective

- Overview of previous mild hypothermia trial
- Overview of current trial
  - Review of objectives of trial
  - Review of questions to be answered by the trial
  - Review of target enrollment and time frame
  - Review of inclusion criteria

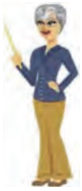

What is the Study?

- Collaborative research effort w/UCSF, OHSU, UNOS, & your OPO
  - UCSF – IRB Institute of Record
- Funded by the Arnold Foundation
- Phase II
  - Hypothermia & machine perfusion better together?
- Prospective randomized trial across multi ple OPOs

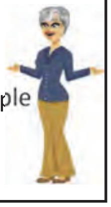

Background/Previous Trial

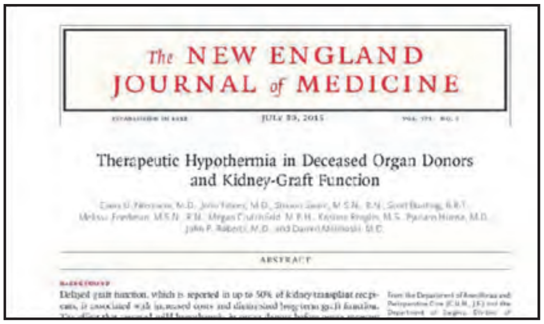
The NEW ENGLAND JOURNAL of MEDICINE

Therapeutic Hypothermia in Deceased Organ Donors and Kidney-Graft Function

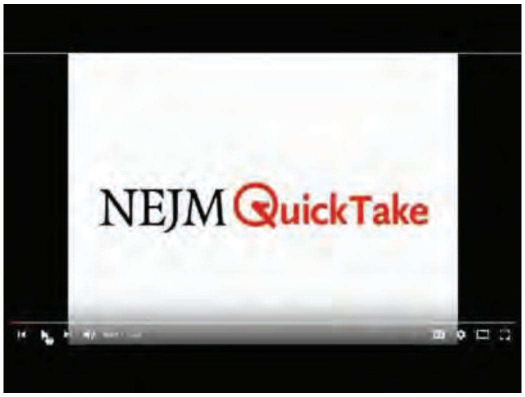

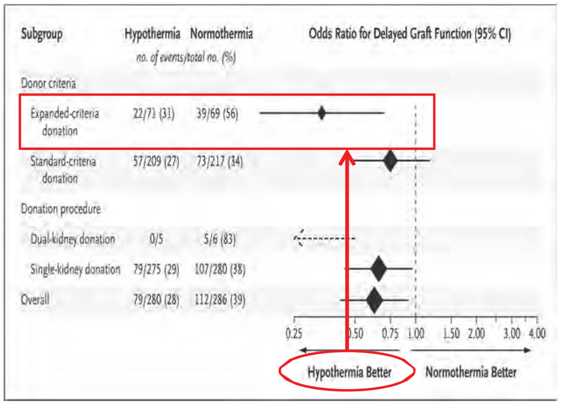

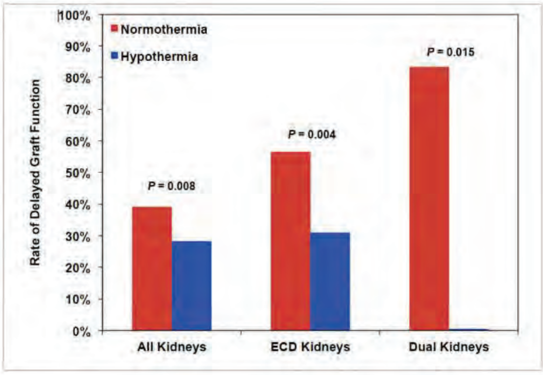

Main Objective of Current Trial

- Evaluate 2 strategies for improving function of kidneys for transplant:
  - Mild hypothermia applied to the donor before organ procurement
  - The use of machine perfusion of the kidney after procurement
    - Based on OPO pump criteria

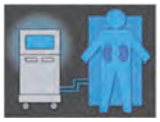

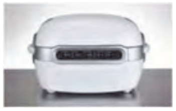

Questions to be Answered

- Is mild hypothermia alone **OR** machine perfusion alone better in protection of pump eligible kidneys?
- Is mild hypothermia **AND** machine perfusion better n protect on of pump e g b e k dneys?
- Is mild hypothermia superior to normothermia in the donors who are not pump e g b e?
- Is mild hypothermia a safe intervention for extra renal organs?

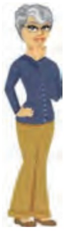

Inclusion Criteria & Target Enrollment

- Brain dead donors
- 18 years or older
- No dialysis
  - ESRD
  - During current admission
- Research authorization
  - Via registry/FPA
  - From authorizing party

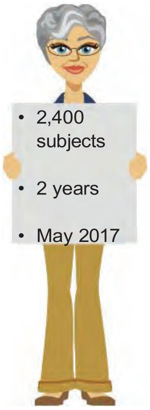

- 2,400 subjects
- 2 years
- May 2017

Objectives - Donor Management Coordinators

- Review enrollment criteria
- Discuss strategies for implementation & maintenance of mild hypothermia during donor management
- Discuss management strategies in the recovery room
- How to document data on the study algorithm sheet

Enrollment Eligibility

- Determine suitability at ~12 hours after start of management
  - Vasopressor requirements = hemodynamic stability
    - Neosynephrine ≤ 60mcg/min
    - Levophed ≤ 10mcg/min
    - Dopamine ≤ 10mcg/kg/min
    - T4, solumedrol, vasopressin – any dose OK
    - Epinephrine at any dose excludes enrollment
  - Electrolytes
    - Potassium >3.5
    - Magnesium ≥2.0
  - Coagulation
    - INR < 2.5
    - PTT < 3x normal value
    - Platelet count > 50,000

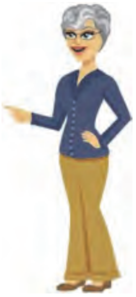

Group Assignments

- Randomized by centralized computer software
  - Normothermia -- 36.5 – 37.5C (97.7 – 99.5F)
  - Hypothermia -- 34 - 35C (93.2 – 95F)
  - Pump determination will not impact donor management

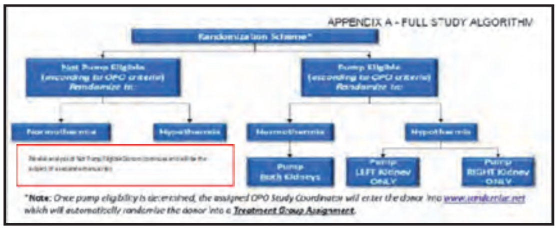

Mild Hypothermia (34-35C)

- Record starting temp (core)– rectal/foley/esophageal/PA
- Cooling/warming device
  - Water blanket (under donor)
    - Set at 30C until temp within 1-2° of target then set at target temp
      - Place bath blanket over top
  - Arctic Sun
    - Set at target temp
  - Bair Hugger
    - set at lowest setting until target temperature reached then to ambient setting

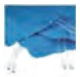
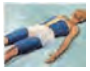
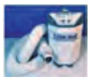

Mild Hypothermia 34-35°

- Ice packs
- Ambient

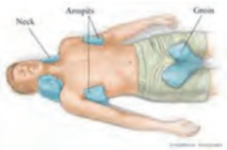

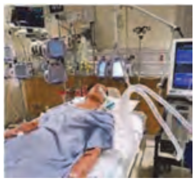

Mild Hypothermia (34-35C)

- Goal: 4 hours to reach target temp
  - ≥ 12 hours maintain at target temp
- Check temp Q1H & record

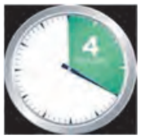

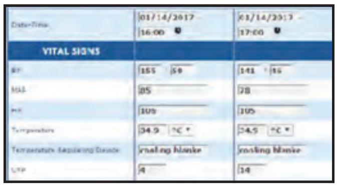

Transporting w/Mild Hypothermia

- Warm blanket over head
- Warm blanket over body
- Warming devices for prolonged procedures
- Increase room temperature
- Reduce exposure
- If Arctic Sun – pads insulate up to 45 minutes
- Goal – not allow temp to drop below 34C
  - Monitor temp if at all possible

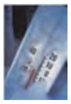

Going to the OR

- Warm blanket over head & body during transport
- Warming blanket under body
  - Between sheet and table
  - Set to 36C
- Bair Hugger over legs
  - Medium to high setting
- Turn the devices off at the time heparin is given or within 5 minutes of cross-clamp

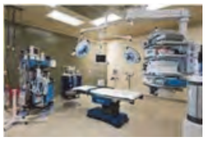

Electrolyte Management

- Monitor electrolytes closely & replace per protocol

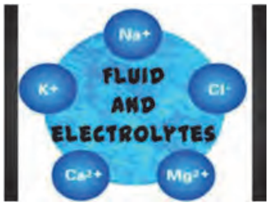

Normothermia (36.5-37.5C)

- Record starting temp (core)
- Check temp Q1H & record
- Should not require any intervention beyond normal donor management

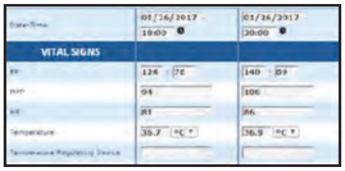

Potential Adverse Effects of Mild Hypothermia

- The degree of hypothermia is mild
- Adverse effects in this temperature range are not reported
  - In the literature adverse effects have been occasionally reported with moderate hypothermia (31-33 C)
- If the following occurs contact the Study Coordinator:
  - Hemodynamic instability
  - Increased urine output (not related to DI or glycosuria)
  - Coagulopathy (INR >2.5, PTT>3x nml, plt<50K)
  - Electrolyte depletion (K, Mg, Phos)
  - Arrhythmias

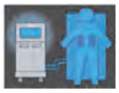

Recording & Monitoring Temperature

- Data collection on algorithm sheet by onsite coordinators/Study Coordinators
- Ensure core temp
  - Foley/rectal/esophageal/PA line
- Record temp Q1H in electronic donor record
- Ensure access to cooling/warming device/method

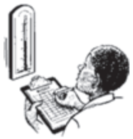

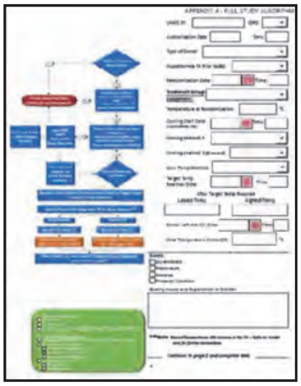

Please attach the completed document to the electronic donor record

Enter requested information here

This section is primarily for the study coordinator to complete

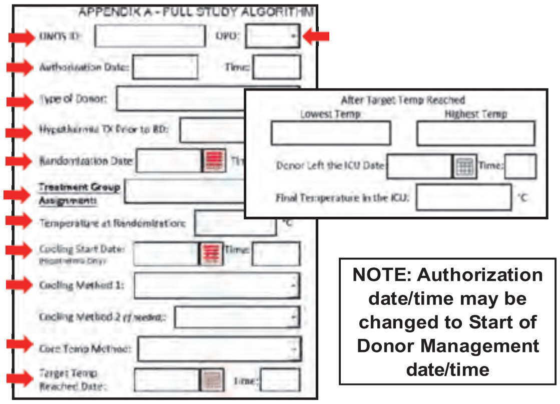

NOTE: Authorization date/time may be changed to Start of Donor Management date/time

Monitoring the Trial

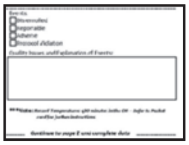

- Reportable event
  - Clinical event that is self-limiting
- Adverse event
  - Clinical event that requires intervention
- Protocol violation
  - Parameters that fall outside of range
- Dis-enrollment
  - Removed from the study altogether for any reason

Communication

- How Transplant Centers Notified
  - General message to UNOS in eNewsletter
  - A study summary & UCSF IRB review will be attached to DonorNet for each subject enrolled
  - Scripted message regarding enrollment will be placed in donor highlights

UNOS Transplant Pro

Objectives – Pump Criteria

- Overview of determination for pump eligibility
- Review randomization strategy
- Discuss data collection

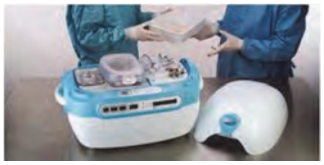

Pumping of Kidneys

- Randomization to pumping will be determined up front when eligibility met based on pre-defined OPO pump criteria

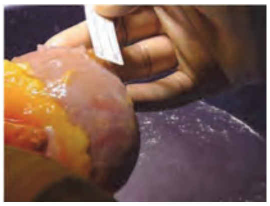

Current Pump Criteria

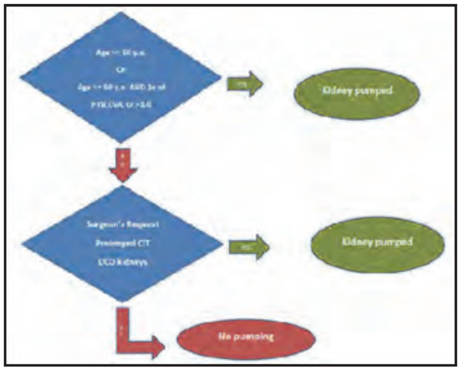

A Closer Look at Pump Randomization

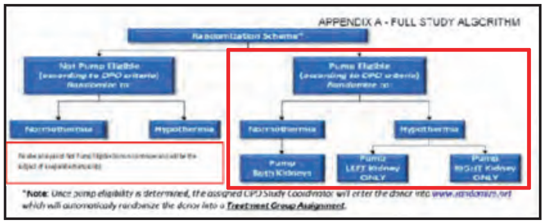

Recording the Data

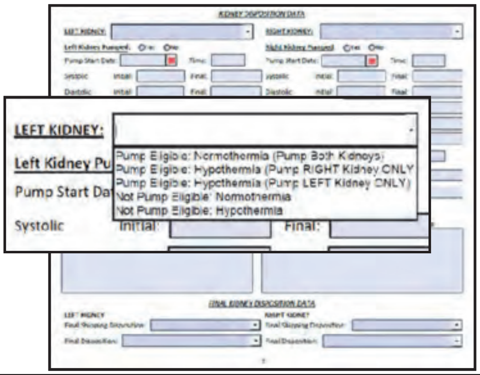

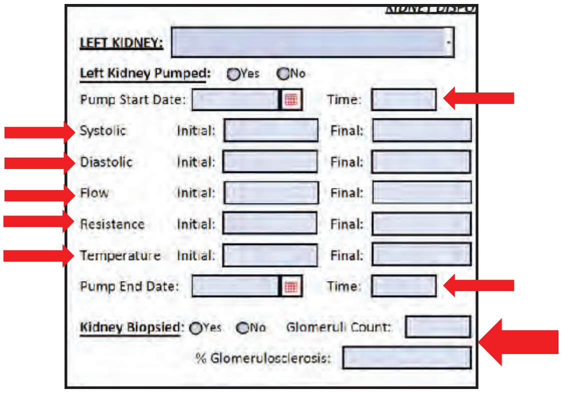

Deviation of Assignment

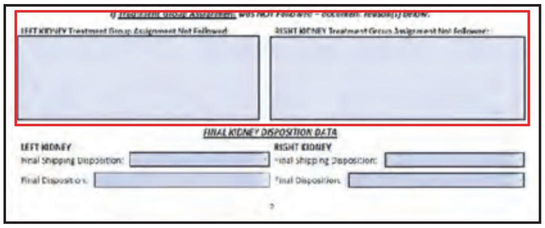

Final Disposition

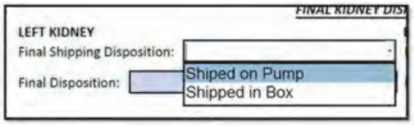

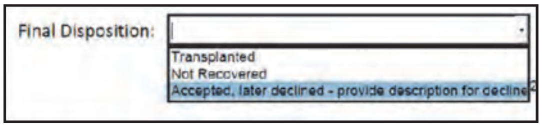

Who to Contact

- Study Coordinator for your OPO
- Local site PI

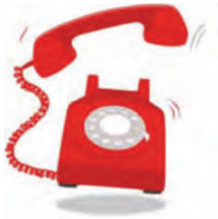

Principal Investigators

Darren Malinoski, MD

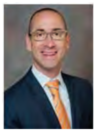

Portland VA Medical Center
PO Box 1034/P3SURG
Portland, OR 97207
& Oregon Health & Science University
malinosk@ohsu.edu

Claus Niemann, MD

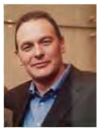

University of California San Francisco,
500 Parnassus MUE 4th Floor
Box 0648, San Francisco, Ca 94143-0648
Claus.Niemann@ucsf.edu, 415-502-2162

Study and Data Coordinating Center

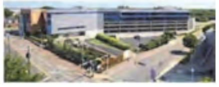

- **UNOS Business Services**

  700 N. 4th St, Richmond, Virginia. 23219

- **Trial Manager**: Alex Garza
- **Data Analytics Manager**: Sarah Taranto
- **Data Quality**: Catherine Monstello, Brooke Chenault, Robert Carrico, PhD
- **Administrative Oversight**: Ryan Ehrensberger

Independent Data Safety and Monitoring Board

Roger J. Lewis, MD, PhD, Department of Emergency Medicine Harbor-UCLA Medical Center, Bldg D9, 1000 W Carson St, Torrance, CA 90509 Torrance, CA, USA

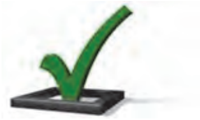

Thank You!

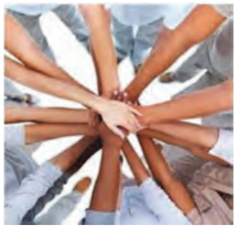

Communication

Participating Donor Service Areas:

Southwest Transplant Alliance – Greater Dallas area
LifeGift – Houston and Fort Worth
areas Other sites TBD

Transplant Community at Large:

The UNOS Region 5 Research Committee has already approved the protocol. Notification will be sent via the United Network for Organ Sharing (UNOS) eNewsletter to inform the transplant community at large about the upcoming study and to allow for a two-week public comment period, during which time questions can be asked. Study protocols will be made available on http://www.transplantpro.org and shared as requested.

Organ Offer to Transplant Centers:

With each subject enrollment, information about organs offered for transplantation from enrolled DNDDs will be posted on DonorNet®, (Richmond, Virginia, USA). In addition to standard data, it will contain information about the study protocol and potential treatment group assignments based on pump eligibility status. This allows any transplant center and surgeon to contact the PI or Co-PI at any time if there are questions concerning donor enrollment and the implications for organs offered for transplantation. The allocation of organs to specific recipients will occur based on standard OPTN guidelines and local transplant center acceptance criteria.

Data Collection

Document Templates

Documents listed below are provided in separate files.

21. Trial Summary
22. Study Algorithm
23. Enrollment Instructions
24. Event Form

Data Collection / Coordination

Data that will be specifically collected for this study are illustrated in **Appendix A**. These data sheets will be sent to UNOS Business Services, the study and data coordination site for the trial. UNOS will combine these unique trial data with other existing data sources in a de-identified, secure manner and send complete data sets to the DSMB for the trial. Using this approach, the study team members never have access to any identifiable information related to living individuals.

Donor-Specific Data

Donor demographic, medical history, and critical care data contained within the UNOS Donor Management Goals Registry will be combined with the unique trial data. Creatinine levels are used for calculation of GFR (Modification of Diet in Renal Disease Study, http://www.nephron.com/MDRD_GFR.cgi). Creatinine and GFR are measured at study enrollment and just prior to organ recovery (terminal creatinine). The Kidney Donor Profile Index (KDPI), a cumulative percentage scale that represents an overall estimate of the risk of graft failure for an individual donor, is contained within the DMG Registry and will be compared between groups. The KDPI includes age, weight, height, ethnicity/race, history of hypertension or diabetes, cause of death, serum creatinine, HCV status, and whether or not the organ donor meets DCD criteria.

Recipient-Specific Data

UNOS will combine the unique trial data elements with transplant recipient outcome and risk factor data contained within their administrative UNet database. Transplant centers are required to submit standardized clinical data elements on all organ recipients for the purposes of regulatory oversight. These data elements are also available to the general public for research purposes. We will only be utilizing these existing data elements and there will be no interaction with recipients and no data collection from recipients for the purpose of this study.

Recipient variables known to impact graft function and that are included in established Scientific Registry of Transplant Recipient (SRTR) risk-adjusted models will be combined with the donor data elements listed above and sent to the DSMB. These variables include recipient age, sex, ethnicity, warm ischemia time, and cold ischemia time, among other various data points. The full models are available at: http://www.srtr.org/csr/current/Centers/201412/modtabs/Risk/KIADC2G.pdf.

Trial Coordination Team

The Trial Coordination Team will consist of the PIs, Darren Malinoski, MD and Claus Niemann, MD; one representative from each OPO/DSA, Alex Garza, Sharon Swain, RN, MSN; and Jenna Graciano, MBA. The committee will discuss weekly progress of the study including rates of organ donor enrollment, protocol compliance, reportable or adverse events, and integrity of data collection.

Data Safety Monitoring Board (DSMB):

The DSMB will consist of Roger Lewis, MD, PhD and Berry Consultants.

Trial Timeline

**Quarters starting July 2018**	1	2	3	4	5	6	7	8	9	10	11	12
Orientation/Training first 3 OPOs	x											
Implementation of Protocols first 3 OPOs	x	x										
Database Training first 3 OPOs	x											
Enrollment of DNDD first 3 OPOs		x	x	x	x	x	x	x	x	x	x	x
Orientation/Training additional 3-5 OPOs		x										
Implementation of Protocols additional 3-5 OPOs		x	x									
Database Training Additional 3-5 OPOs		x										
Enrollment of DNDD Additional 3-5 OPOs			x	x	x	x	x	x	x	x	x	x
Collection DMG		x	x	x	x	x	x	x	x	x	x	x
Interim-Analysis*												
Final-Analysis												x
Manuscripts								x				x

* Exact time TBD and depending on enrollment progress. Each donor population will be monitored separately

RCT Fillable Enrollment Form

May 19, 2020

UNOS ID: | OPO:

Authorization Date: | Time:

Type of Donor: | Pump Eligible:

Hypothermia TX Prior to BD:

Randomization Date: | Time:

Treatment Group

°C

(Should be above 36.5 prior to randomization)

Start Date: | Time:

Temperature Regulation Method 1:

Temperature Regulation Method 2:
(if needed)

Time:

After Target Temp Reached

Lowest Temp | Highest Temp

Donor Left the ICU Date: | Time:

Final Temperature in the ICU: °C

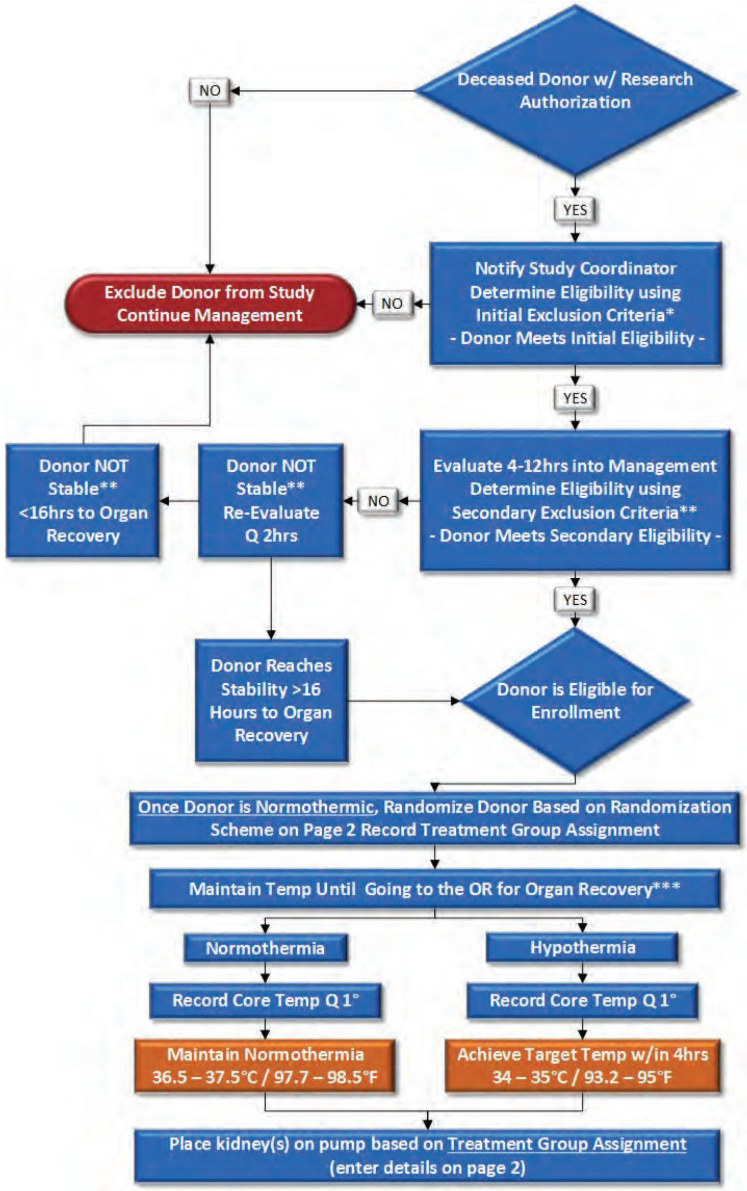

Events:

- ☐ Reportable
- ☐ Adverse
- ☐ Protocol Violation
- ☐ Intended Pumping Assignment Could not be Followed
  - ☐ Kidney assigned to pump but not pumped
  - ☐ Kidney assigned to no pump but pumped
- ☐ Dis-enrolled - Use the text box below to describe reason(s) for dis-enrollment, such as confirmed dual kidney allocation, family withdraws authorization, or exclusion criteria met after enrollment but prior to initiation of cooling.

Quality Issues and Explanation of Events:

*Initial Exclusion Criteria
1. DCD Donor
2. Age <18 years
3. End Stage renal Disease (ESRD) or Dialysis during current admission

**Secondary Exclusion Criteria
1. Coagulopathy (INR>2.5, PTT >3x normal value, &/or PLT <50,000)
2. Hemodynamic Instability w/ either:
   - Dopamine >10mcg/kg/min
   - Norepinephrine >0.2mcg/kg/min
   - Neosynephrine >1mcg/kg/min
   - Epinephrine (any dose)

   If dose in EMR is mcg/min divide by dry weight to convert to mcg/kg/min

   WEIGHT (kg)
3. Electrolytes
   - Potassium < 3.5
   - Magnesium < 2.0
4. Other – Approved by PI

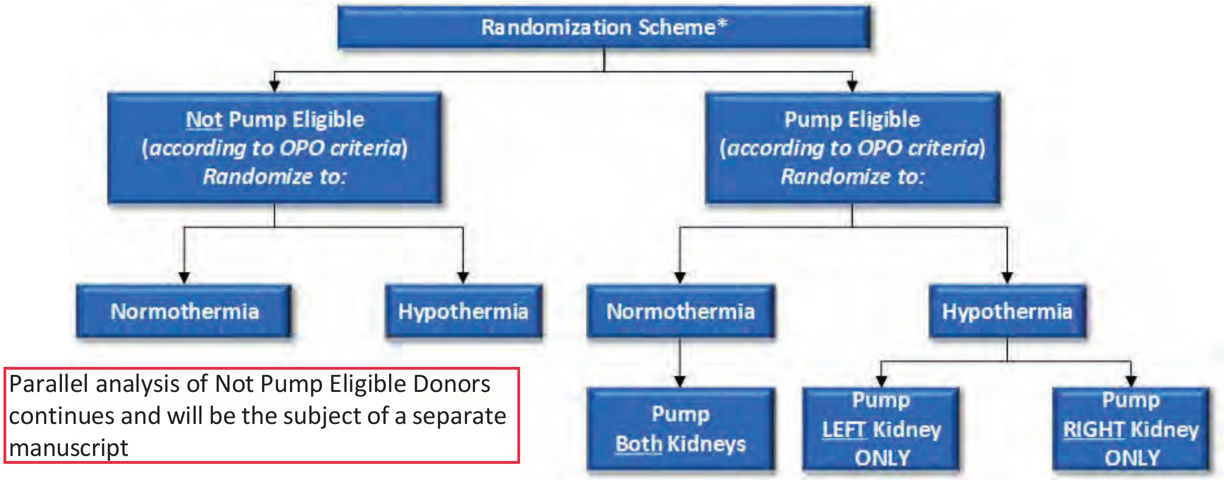

*__Note__: *Once pump eligibility is determined, the assigned OPO Study Coordinator will enter the donor into* www.randomize.net *which will automatically randomize the donor into a* ***Treatment Group Assignment***.

KIDNEY DISPOSITION DATA

LEFT KIDNEY:

Accompanied by: ☐ Heart ☐ Intestine ☐ Liver ☐ Lung

Left Kidney Pumped: ○ Yes ○ No

Pump Start Date: ______ Time: ______

	Initial:	Final:
Systolic		
Diastolic		
Flow (cc/min)		
Resistance		
Temperature ˚C		

Pump End Date: ______ Time: ______

Kidney Biopsied: ○ Yes ○ No Glomeruli Count: ______

% Glomerulosclerosis: ______

RIGHT KIDNEY:

Accompanied by: ☐ Heart ☐ Intestine ☐ Liver ☐ Lung

Right Kidney Pumped: ○ Yes ○ No

Pump Start Date: ______ Time: ______

	Initial:	Final:
Systolic		
Diastolic		
Flow (cc/min)		
Resistance		
Temperature ˚C		

Pump End Date: ______ Time: ______

Kidney Biopsied: ○ Yes ○ No Glomeruli Count: ______

% Glomerulosclerosis: ______

If Treatment Group Assignment was NOT Followed – document reason(s) below:

LEFT KIDNEY Treatment Group Assignment Not Followed:

RIGHT KIDNEY Treatment Group Assignment Not Followed:

FINAL KIDNEY DISPOSITION DATA

LEFT KIDNEY

Final Shipping Disposition: ______

Final Disposition: ______

Decline Reason: ______

RIGHT KIDNEY

Final Shipping Disposition: ______

Final Disposition: ______

Decline Reason: ______

Drop-down Options:

1. **OPO**
   - TXGC
   - TXSB

2. **Type of Donor**
   - ECD
   - SCD

3. **Hypothermia TX Prior to BD**
   - Yes
   - No

4. **Treatment Group Assignment**
   - Pump Eligible: Normothermia (Pump Both Kidneys)
   - Pump Eligible: Hypothermia (Pump RIGHT Kidney ONLY)
   - Pump Eligible: Hypothermia (Pump LEFT Kidney ONLY)
   - Not Pump Eligible: Normothermia
   - Not Pump Eligible: Hypothermia

5. **Cooling Method 1**
   - Cooling Blanket
   - Passive Cooling
   - Bair Hugger
   - Ice Packs
   - Artic Sun
   - Other

6. **Cooling Method 2**
   - Cooling Blanket
   - Passive Cooling
   - Bair Hugger
   - Ice Packs
   - Artic Sun
   - Other

7. **Core Temp Method**
   - Rectal
   - Bladder
   - Esophageal
   - PA Line

8. **R/L Kidney Shipping Disposition**
   - Shipped on Pump
   - Shipped in Box

9. **R/L Kidney Final Disposition**
   - Transplanted
   - Not Recovered
   - Accepted, later declined – provide description for decline

UCSF
University of California
San Francisco

Human Research Protection Program
Institutional Review Board (IRB)

Full Committee Approval

Principal Investigator
Dr. Claus Niemann, MD

Type of Submission: Submission Response for Initial Review Submission Packet
Study Title: A Randomized Trial of Mild Hypothermia and Machine Perfusion in Deceased Organ Donors for Protection against Delayed Graft Function in Kidney Transplant Recipients

IRB #: 17-21768
Reference #: 185808
Reviewing Committee: Parnassus Panel
Study Risk Assignment: Minimal

Approval Date: 04/17/2017 **Expiration Date:** 04/16/2018

Regulatory Determinations Pertaining to This Approval:

Note: This study involves deceased organ donors and their kidney recipients. The donors are not considered human subjects under Health and Human Services Code of Federal Regulations Title 45 Part 46. The following regulatory determinations were made regarding the recipients only.

This research is not subject to HIPAA rules.

A waiver of informed consent is acceptable because, as detailed in the application: (1) the research involves no more than minimal risk to the organ recipients; (2) the waiver will not adversely affect the rights and welfare of the organ recipients; (3) the research could not practicably be carried out without the waiver; and (4) whenever appropriate, the organ recipients will be provided with additional pertinent information after participation. The waiver of informed consent applies to all persons receiving a kidney managed by this protocol.

This research satisfies the following condition for the involvement of children: 45 CFR 46.404, 21 CFR 50.51: Research not involving greater than minimal risk.

The full board determined that the research poses no greater than minimal risk and is eligible for expedited review in the future under category #9 (continuing review of research, not conducted under an IND or IDE where categories 2 through 8 do not apply but the IRB has determined and documented at a convened meeting that the research involves no greater than minimal risk and no additional risks have been identified).

Comment:
Per the Office of Human Research Protections' definition of "engagement in research," the hospitals at which the recipients receive their transplants are *not* engaged in research because they are not performing any research procedures.

All changes to a study must receive UCSF IRB approval before they are implemented. Follow the modification request instructions. The only exception to the requirement for prior UCSF IRB review and approval is when the changes are necessary to eliminate apparent immediate hazards to the subject (45 CFR 46.103.b.4, 21 CFR 56.108.a). In such cases, report the actions taken by following these instructions.

Expiration Notice: The iRIS system will generate an email notification eight weeks prior to the expiration of this study's approval. However, it is your responsibility to ensure that an application for continuing review approval has been submitted by the required time. In addition, you are required to submit a study closeout report at the completion of the project.

Documents Reviewed with this Submission:

Study Document			
Title	Version #	Version Date	Outcome
Letter from UCSF Bioethicist Koenig	Version 1.0	02/20/2017	Approved
Letter from UPenn Bioethicist	Version 1.0	02/16/2017	Approved
Letter from Vanderbilt Bioethicist	Version 1.0	02/16/2017	Approved
Public Citizen complaint letter	Version 1.0	02/16/2017	Approved
UCSF Response re Public Citizen	Version 1.0	02/16/2017	Approved
Study Diagrams	Version 1.0	02/16/2017	Approved
Niemann NEJM 2015 Article on Previous Trial	Version 1.0	02/16/2017	Approved
Niemann Heldens Editorial on Donor Research	Version 1.0	02/16/2017	Approved
Watson_Editorial_NEJM_2015	Version 1.0	02/16/2017	Approved
Feng-2016-American_Journal_of_Transplantation	Version 1.0	02/16/2017	Approved
Abt_et_al-2016-American_Journal_of_Transplantation	Version 1.0	02/16/2017	Approved

For a list of all currently approved documents, follow these steps: Go to My Studies and open the study – Click on Informed Consent to obtain a list of approved consent documents and Other Study Documents for a list of other approved documents.

San Francisco Veterans Affairs Medical Center (SFVAMC): If the SFVAMC is engaged in this research, you must secure approval of the VA Research & Development Committee in addition to UCSF IRB approval and follow all applicable VA and other federal requirements. The IRB website has more information.

UCSF
University of California
San Francisco

Human Research Protection Program
Institutional Review Board (IRB)

Expedited Review Approval

Principal Investigator
Dr. Claus Niemann MD

Type of Submission: Continuing Review Submission Form
Study Title: A Randomized Trial of Mild Hypothermia and Machine Perfusion in Deceased Organ Donors for Protection against Delayed Graft Function in Kidney Transplant Recipients

IRB #: 17-21768
Reference #: 267104
Committee of Record: Parnassus Panel
Study Risk Assignment: Minimal

Approval Date: 12/02/2019 **Expiration Date:** 12/01/2020

Regulatory Determinations Pertaining to this Approval:
This research satisfies the following condition(s) for the involvement of children:
45 CFR 46.404, 21 CFR 50.51: Research not involving greater than minimal risk.

This research is not subject to HIPAA rules.

A waiver or alteration of informed consent is acceptable because, as detailed in the application: (1) the research involves no more than minimal risk to the subjects; (2) the waiver or alteration will not adversely affect the rights and welfare of the subjects; (3) the research could not practicably be carried out without the waiver or alteration; and (4) whenever appropriate, the subjects will be provided with additional pertinent information after participation. The waiver of informed consent applies to all persons receiving a kidney managed by this protocol.

This submission was eligible for expedited review as:
Category 9: Renewal of other minimal risk research protocols: Continuing review of research, not conducted under an IND or IDE where categories 2 through 8 do not apply but the IRB has determined and documented at a convened meeting that the research involves no greater than minimal risk and no additional risks have been identified

All changes to a study must receive UCSF IRB approval before they are implemented. Follow the modification request instructions. The only exception to the requirement for prior UCSF IRB review and approval is when the changes are necessary to eliminate apparent immediate hazards to the subject (45 CFR 46.103.b.4, 21 CFR 56.108.a). In such cases, report the actions taken by following these instructions.

Expiration Notice: The iRIS system will generate an email notification eight weeks prior to the expiration of this study's approval. However, it is your responsibility to ensure that an application for continuing review approval has been submitted by the required time. In addition, you are required to submit a study

closeout report at the completion of the project.

For a list of all currently approved documents, follow these steps: Go to My Studies and open the study – Click on Informed Consent to obtain a list of approved consent documents and Other Study Documents for a list of other approved documents.

San Francisco Veterans Affairs Medical Center (SFVAMC): If the SFVAMC is engaged in this research, you must secure approval of the VA Research & Development Committee in addition to UCSF IRB approval and follow all applicable VA and other federal requirements. The UCSF IRB website has more information.

DMRI DONOR MANAGEMENT RESEARCH INITIATIVE

January 18, 2020

DSMB Interim Analysis Recommendation:

The DSMB of the randomized trial comparing donor hypothermia, machine perfusion, or both for the prevention of delayed graft function (DFG) in transplanted kidneys met by teleconference on **January 17, 2020** to evaluate DGF outcome results from the first 600 pump-eligible donors, as required by the pre-specified interim analysis plan. Based on a review of the available data, congruent with the prespecified study design, the DSMB recommends termination of randomization into the hypothermia-only arm and continuation of the trial in pump-eligible donors with 1:1 randomization to machine perfusion alone or machine perfusion combined with donor hypothermia. The next planned interim analysis for this population will occur when DGF outcomes are available from 800 donors.

Roger J. Lewis, MD, PhD
Chair, DSMB and Senior Medical Scientist, Berry Consultants, LLC

Figure 1 – Randomization scheme after DSMB recommendation

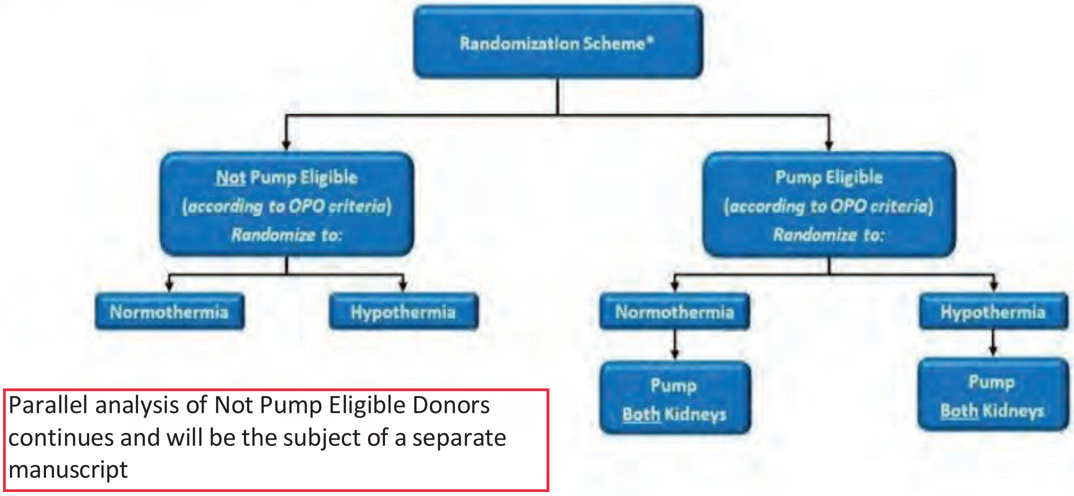

Parallel analysis of Not Pump Eligible Donors continues and will be the subject of a separate manuscript

DONOR MANAGEMENT RESEARCH INITIATIVE

June 8, 2020

DSMB Interim Analysis Recommendation:

The DSMB for your randomized trial comparing donor hypothermia, machine perfusion, or both for the prevention of delayed graft function (DGF) in transplanted kidneys met by teleconference on **May 19, 2020** to evaluate DGF outcome results from the first 800 pump eligible donors, as required by the pre-specified interim analysis plan. Based on a review of the available data, congruent with the pre-specified study design, the DSMB recommends termination of randomization for the trial in pump eligible donors because this population met the trial pre-specified criteria for futility. The not pump eligible donor population is to proceed according to the protocol with the first planned interim analysis for the not pump eligible population to occur when DGF outcomes are available from 600 donors.

Roger J. Lewis, MD, PhD
Chair, DSMB and Senior Medical Scientist, Berry Consultants, LLC

Figure 1 - Randomization scheme after DSMB recommendation:

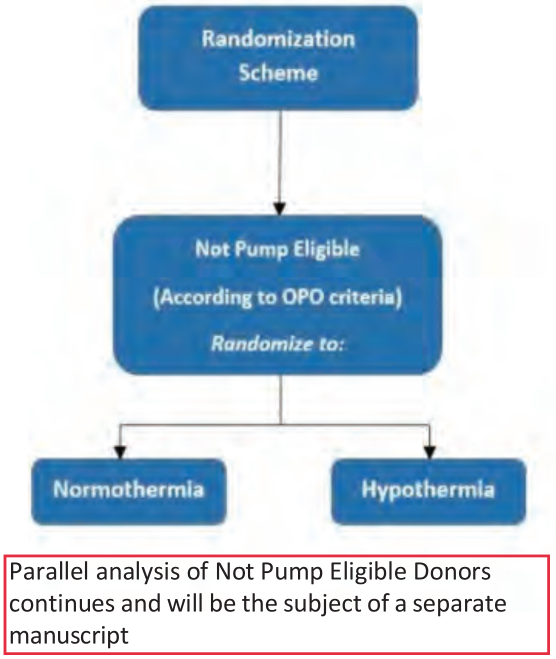

Acknowledgements

Institution of record for the trial:

University of California San Francisco

Local Institutional Advisory Panel:

Daniel Lowenstein, MD
Executive Vice Chancellor and Provost
University of California San Francisco
Lowenstein@ucsf.edu

Edward Kuczynksi, MA
Director, Human Research Protection Program
University of California, San Francisco
edward.kuczynski@ucsf.edu

Barbara Koening, PhD, RN
Director, Bioethics, University of California San Francisco
Medical Anthropology and Bioethics, University of California San Francisco
Barbara.Koenig@ucsf.edu

Katie Nolan, MPH, CIP
Regulatory Knowledge & Support (RKS)
Human Research Protection Program
University of California, San Francisco
Kate.Nolan@ucsf.edu

We also would like to acknowledge the continuous support and expertise of:

David Klassen, MD
Chief Medical Officer for United Network for Organ Sharing (UNOS), Richmond, VA

Ryan Ehrensberger, PhD, FACHE
Chief Growth Officer, United Network for Organ Sharing (UNOS), Richmond, VA

Summary of Protocol Modifications

Protocol Modification	Date
Southwest Transplant Alliance	July 26, 2017 (Trial Launch)
Organ Procurement Organization Site Added - LifeGift	January 8, 2018
Organ Procurement Organization Site Added - Donor Alliance	June 4, 2018
Organ Procurement Organization Site Added - Donor Network of Arizona	January 1, 2019
Organ Procurement Organization Site Added - Pacific Northwest Transplant Bank	May 22, 2019
Organ Procurement Organization Site Added - LifeSource	July 24, 2019
DSMB recommends termination of randomization into the hypothermia-only arm (updated hypothermia treatment arm below)	January 17, 2020
Randomization Scheme updated in Randomize.net	January 20, 2020
Statistical Design Revision	February 25, 2020
DSMB recommends termination of randomization for the trial in pump eligible donors	May 19, 2020
Pump Eligible Arm removed in Randomize.net	May 22, 2020
**Administrative Modifications:**<br><br>Additions:<br><br>1. Trials Design and Statistics<br>Christina Saunders, PhD, Berry Consultants<br><br>2. Southwest Transplant Alliance<br>Site Study Coordinator: Erin Vines<br><br>3. Edward Kuczynksi, MA<br>Director, Human Research Protection Program<br>University of California, San Francisco<br><br>Modifications:<br><br>1. Study and Data Coordinating Center<br>"Data Analytics Manager Role" changed to "Transplant Analytics Service Line Leader"<br><br>2. Acknowledgements<br>Ryan Ehrensberger, Ph.D, ~~FACHS~~ FACHE<br>~~Director of Research, Director of UNOS Business Services~~, ~~UNOS, Richmond, CA~~ Chief Growth Officer, United Network for Organ Sharing (UNOS), Richmond, VA	Modifications performed periodically throughout the study

Deletions:<br><br>1. Study and Data Coordinating Center<br>Research Scientist, Robert Carrico, PhD<br><br>2. Consultant<br>Jenna Graciano, MBA, Transplant Quality Manager, St. Vincent Medical Center<br><br>3. Organ Procurement Organization Site<br>Southwest Transplant Alliance<br>Site Study Coordinator: Amen Oyegun<br><br>4. Local Institutional Advisory Panel:<br>Terri O'Lonergan, PhD, MA, Associate Vice Chancellor, Chief Ethics and Compliance Officer<br>Institutional Official: Animal & Human Research, Research Integrity Officer<br><br>5. Local Institutional Advisory Panel:<br>Christopher M. Ryan, Ph.D.<br>Director, Human Research Protection Program<br>Director, CTSI Regulatory Knowledge and Support Program<br><br>6. Acknowledgements<br>Melissa Greenwald, MD<br>Health Resources Services Administration (HRSA)	Modifications performed periodically throughout the study

Design Revision for the Randomized Trial of Mild Hypothermia and Machine Perfusion in Deceased Organ Donors for Protection against Delayed Graft Function (DGF): The Pump Eligible Population

Berry Consultants LLC

February 25, 2020

This document describes an update to the statistical design for the pump eligible population only enrolled in the Randomized Trial of Mild Hypothermia and Machine Perfusion in Deceased Organ Donors for Protection against Delayed Graft Function (DGF). In the pump eligible population of this trial donors and their kidneys were randomized to receive machine perfusion (pump) only, hypothermia only, or both machine perfusion and hypothermia (combination). The maximum planned sample size was 1400 donors. Interim analyses to drop inferior arms were planned, beginning when 600 donors had complete DGF outcomes and after every additional 200 donors.

On January 17, 2020 the trial's Data Safety Monitoring Board (DSMB) met to review the results of the first interim analysis with complete DGF outcomes from 600 donors. In agreement with the pre-specified statistical design, the DSMB recommended enrollment to the hypothermia alone arm be stopped for inferiority against the other two arms in the trial. Currently the trial continues enrollment and randomization with donors being randomized equally between the pump and combination arms.

Beginning at the next planned interim analysis with complete DGF outcomes for 800 total donors, three revisions in the statistical design will be implemented. These design revisions are being made by study personnel that remain blinded to the trial data in light of the clinical questions of interest and the feasibility of continued accrual to the trial. These revisions include

1. The comparison between the pump alone and combination arms will become a one-sided hypothesis test considering only the superiority of the combination over pump alone.
2. A futility stopping boundary will be added for the comparison between the pump alone and the combination arms
3. The maximum planned sample size will be reduced to 1200 donors

Stopping Boundaries

The table below provides the two-sided nominal p-values for both the success and the futility boundaries.

Total Donors	Nominal Two-Sided P-Value for Success	Nominal Two-Sided P-value for Futility
600	0.0028	0.7936
800	0.0174	0.4901
1000	0.0269	0.2337
1200	0.0391	--

Discussion of the Success Boundary

At the time of this update, the interim analysis with 600 donors has already occurred with a total two-sided alpha spend of 0.0028 out of the total two-sided alpha of 0.05. In order to calculate the revised success boundary, taking into account the lower maximum sample size, we maintain the alpha spend at the first interim analysis and calculate the remaining alpha spend at each successive look according to the revised information fraction with the Lan DeMets approximation to the O'Brien-Fleming spending function. We allow a total two-sided alpha spend for the successive looks of 0.05-0.0028 = 0.0472. With the alpha spend at each look determined in this manner, we use a piecewise linear spending function to determine the nominal p-values required at each look to stop early for success.

Discussion of the Futility Boundary

The futility boundary is based on the Hwang-Shih-Decani spending function with a gamma parameter of -2. The futility boundary in this trial is considered non-binding. Because the futility boundary is non-binding, and the first interim analysis has already been conducted at the time these revisions are to be implemented, futility at the 600 donor interim will not be evaluated. The futility boundary will be implemented as specified above beginning at the 800 donor interim analysis.

Implementation Note

While the hypotheses in this population were originally specified, statistically, as two-sided, there is currently only interest in the one-sided hypothesis test of whether the combination would be superior to pump alone. The p-values provided from the primary analysis are two-sided and the nominal p-values required for early stopping are specified as two-sided. However, the implementation is to be one-sided. This implementation is straightforward. To stop early for success, the two-sided p-value must be less than the specified nominal p-value and directionality of the effect must favor the combination arm. To stop early for futility, either the directionality of the effect must favor the pump alone arm, or if the directionality of the effect favors the combination arm, the two-sided p-value must be greater than the specified nominal p-value.

Consistent with the original trial design, the interim analysis milestones and final analysis are inclusive of all randomized donors. Therefore, the planned maximum sample size of

1200 is inclusive of the donors and kidneys randomized to the hypothermia alone arm even though donors are no longer being randomized to that arm.

Operating Characteristics

We simulate the design revisions for the two remaining arms of the trial to understand the overall Type I error rate, power, and probabilities for early success and early futility. The simulations make two simplifying assumptions. First, we assume that 200 donors were enrolled to the hypothermia alone arm and therefore, at each interim, 200 donors are not included in the comparison of interest. For example, at the final sample size of 1200, we assume that 1000 donors will be included in the comparison of pump alone to the combination arm. The second simplifying assumption is that we assume that donors on the two arms of interest were equally randomized between them from the beginning of the trial. This assumption likely overestimates the correlation between kidneys on the arms and therefore may slightly underestimate the available power.

The table below demonstrates the operating characteristics for the comparison between the pump alone and combination arms of the trial given the design changes. As the first interim analysis with 600 donors has passed, no early stopping was allowed at that first look in the simulations. Simulations assumed a 30% DGF rate on the pump alone arm and a correlation between kidneys of 0.15, consistent with the original trial design simulations. Operating characteristics are based on 2500 simulations per scenario.

Absolute Improvement in DGF	Total Pr(Success)	Pr (Early Success)	Pr (Early Futility)	Mean Number of Donors in the Comparison
0% (Null)	0.025	0.0172	0.881	670
5%	0.497	0.354	0.314	787
7.5%	0.830	0.70	0.079	739
10%	0.982	0.942	0.008	650

The plot below shows all simulated trials. The y-axis is the absolute difference in DGF rates between the arms where a lower difference is a better outcome for the combination arm. The x-axis shows the different looks of the trial where look 1 is the interim analysis with 600 donors and look 4 is the final analysis with 1200 donors. Each simulated trial is a dot where red dots are futile trials and green dots are successful trials. Trials that appear at looks 2 or 3 stopped early and trials that appear at look 4 ran to the maximum sample size of 1200 donors. Therefore, this plot demonstrates the observed differences between the arms that would result in success or futility at each look. The smallest absolute difference between the combination arm and the pump alone arm that was observed to result in a successful trial across all simulated trials was a 4.6% benefit for the combination arm.

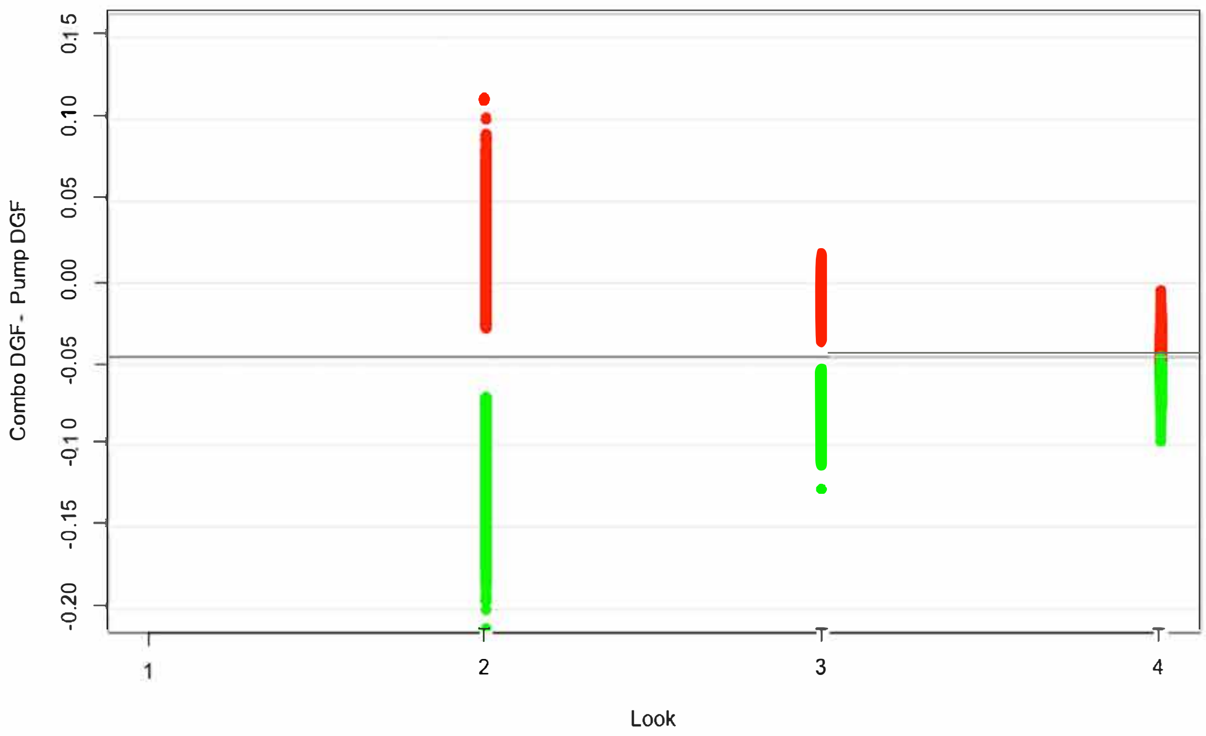

Combo DGF - Pump DGF
0.15
0.10
0.05
0.00
-0.05
-0.10
-0.15
-0.20
1
2
3
4
Look

Summary of Statistical Analysis Changes:

After the DSMB recommended enrollment to the hypothermia alone arm be stopped for inferiority against the other two arms in the trial, the trial proceeded with equal randomization between the pump and combination arms. In light of this, the design revision specified the following three changes: reduced the maximum planned sample size from 1400 to 1200 donors; the comparison between the pump alone and combination arms at future interim analyses became a one-sided hypothesis test, considering only the superiority of the combination over pump alone; and a futility stopping boundary was added for the comparison between the pump alone and the combination arms.
