# Supplementary material for: Therapeutic Hypothermia in Low-Risk Nonpumped Brain-Dead Kidney Donors: A Randomized Clinical Trial
Source: JAMA Netw Open. 2024 Feb 28;7(2):e2353785. doi: 10.1001/jamanetworkopen.2023.53785 (PMC10902731; doi:10.1001/jamanetworkopen.2023.53785)
Supplement: Supplement 2. — Data Sharing Statement [file jamanetwopen-e2353785-s002.pdf]

Data Sharing Statement

Patel. Therapeutic Hypothermia in Low-Risk Nonpumped Brain-Dead Kidney Donors. *JAMA Netw Open*. Published February 28, 2024. doi:10.1001/jamanetworkopen.2023.53785

Data
Data available: No
